# Integrative modelling of innate immune response dynamics during virus infection

**DOI:** 10.1371/journal.pcbi.1014395

**Published:** 2026-06-22

**Authors:** Ramya Boddepalli, Harsh Chhajer, Rahul Roy

**Affiliations:** 1 Department of Chemical Engineering, Indian Institute of Science, Bangalore, India; 2 Department of Bioengineering, Indian Institute of Science, Bangalore, India; Los Alamos National Laboratory, UNITED STATES OF AMERICA

## Abstract

Positive-sense RNA viruses that constitute a large class of human pathogens employ various strategies to suppress and evade host immune defenses. Understanding the dynamic interaction between the viral life cycle and immune signaling is crucial to designing effective antiviral strategies. Although significant progress has been made, quantitative models that can accurately capture the intricate interactions and the intertwined dynamics during viral infection of cells remain missing. In this study, we develop a comprehensive mathematical model that integrates the intracellular viral life cycle with key cellular innate immune pathways, including RIG-I-mediated detection and JAK-STAT signaling. The model provides mechanistic insights into long-standing observations, capturing both virus-specific dynamics and innate immune response, and the key components driving their coupled dynamics. For example, a comparison of viruses shows how the Japanese Encephalitis virus undergoes a dramatic reduction in viral load in cells, due to its rapid replication that robustly activates the RIG-I pathway, in contrast to the poor immune control of Hepatitis C virus. More importantly, our model demonstrates how virus-host interactions exhibit a sharp transition boundary behavior, where minor differences in immune strength or viral suppression capacity can determine whether infections resolve or persist. We propose that ISG mRNA translation and viral replication predominantly dictate these bimodal infection outcomes. Additionally, the model not only recapitulates IFN desensitization but also identifies the molecular players involved. We demonstrate how our model’s ability to capture IFN dynamics allows us to predict optimal timing and dosing strategies for interferon-based prophylactic therapies. Together, our approach reveals fundamental features that govern the delicate balance between the establishment of infection and immune control in RNA virus infections.

## Author summary

Viruses responsible for diseases like hepatitis and dengue rapidly proliferate by invading host cells, which in turn activate immune responses to counteract the infection. Viruses have also developed a variety of mechanisms to interfere with this immune response, thereby complicating the process of effectively treating infections. Here, we simulate the comprehensive sequence of molecular-level actions and the corresponding counteractions that occur between the virus and the host cell components as the infection proceeds. We find that the battle between the virus and the immune system behaves like a seesaw, and small changes can dramatically tip the balance. Sometimes, a tiny boost to our immune response or a small weakness in the virus can mean the difference between cells succumbing to infection or recovering completely. Similarly, it is now possible to recognize why specific viruses are more effectively controlled by the immune system compared to others. We can now explain the decline in the immune response to interferon therapy with prolonged use and suggest the optimal timing and dosing for such treatments. For example, we found that interferon treatment administered before infection, even in small doses, can block the virus more effectively than treatment after infection begins. In addition, we propose better ways to inhibit the virus by effectively combining complementary approaches, such as blocking its replication or its ability to hide from the immune system, which can boost the effectiveness of interferon treatment. This work provides a roadmap for developing quantitative and efficient antiviral approaches by understanding the precise biological mechanisms that determine how hosts can overcome virus infection.

## Introduction

Positive-sense RNA viruses infect hundreds of millions of people annually, leading to widespread morbidity and mortality, and constitute a significant global health burden. Since 2019, COVID-19 has been responsible for ∼700 million cases and 7 million deaths [1]. Similarly, dengue virus accounted for ∼6.5 million cases and ∼7,000 deaths in 2023 [2], while Hepatitis C virus continues to cause over 1 million new infections and ∼242,000 deaths each year [3]. This immense global impact stems from a lack of potent antiviral therapies and poor efficacy of vaccines. Positive-sense RNA ((+)RNA) viruses leverage their ability to hijack host cells, where they manipulate cellular machinery to propagate and evade immune defenses, driving a complex interplay between the virus and the host cell that underpins their pathogenicity. Although experimental approaches have identified many key host and viral molecules and their roles in defining the extent of infection in cells, our understanding of how viruses can overcome host cell immune responses remains limited and fragmented. Developing a deep and quantitative understanding of this cellular-level host-virus dynamics could be key to crafting effective antiviral strategies in the future.

The (+)RNA viral life cycle within the immune cell encompasses several coordinated, interconnected, and counteracting steps. Virus growth proceeds through cell receptor-mediated entry, host cell-assisted viral RNA translation, compartmentalized replication, coordinated assembly, and processing and release of new virions [4–6]. The intricate interplay between viral and host molecular processes is central to the outcome of infection. For instance, viral RNA translation by host cell ribosomes generates structural proteins for viral assembly and non-structural proteins that drive replication and manipulate host cellular processes to create a conducive environment for viral growth. Viral replication synthesizes new viral genomes through a double-stranded RNA (dsRNA) intermediate, which is essential for the further translation and assembly of new virions [4,5,7]. Beyond its role as a replication intermediate, viral dsRNA acts as a potent pathogen-associated molecular pattern (PAMP) that alerts the host to infection once exposed to the cytoplasm. The resulting innate immune response—mediated largely by type I interferon (IFN) and NFκB signaling —constitutes a rapid, ubiquitous defense mechanism capable of restricting a diverse range of viral families. For RNA viruses, this process is initiated when RIG-I detects dsRNA and activates the mitochondrial adaptor MAVS [8–11]. MAVS serves as a signaling hub for the kinases TBK1 and IKKs (IKKα, β,γ, ϵ), which trigger the nuclear translocation of IRF3/7 and NFκB. This transcriptional cascade ultimately induces type I IFNs and a robust antiviral program designed to limit the spread of the pathogen [8,9,12].

As the virus assembles and releases new virions, the host immune system mounts a broader antiviral response via cytokines like IFNs [8,10,11]. Secreted IFNs engage their cognate receptor, IFNAR, on the surface of both the producing cell and adjacent cells, thereby triggering activation of the JAK–STAT signaling pathway. Activated STAT1 and STAT2, together with IRF9, form the ISGF3 complex, which translocates to the nucleus to drive the expression of hundreds of interferon-stimulated genes (ISGs). These ISGs encode a diverse set of antiviral proteins that inhibit various stages of the viral life cycle, including entry, translation, replication, and assembly [9,12]. Given their crucial role in immune signaling, interferons are also employed therapeutically to enhance immune responses. However, sustained or repeated exposure to IFN can result in diminished cellular sensitivity (IFN desensitization), and its clinical application is further limited by the well-established, dose-dependent toxicity linked to IFN therapy [13,14]. The diminished efficacy of IFN’s antiviral function is largely attributed to negative regulators acting at the IFN receptor level [9,15].

The complex interactions between viral mechanisms and host immune defenses result in dynamic and often unpredictable infection outcomes. The reciprocal actions between the virus and immune host cells establish intricate feedback loops, producing duality in the infection trajectories. For instance, ISGs such as Mx (myxovirus resistance) and IFITM proteins inhibit viral entry, while ISG20, 2’,5’-oligoadenylate synthetase (OAS)-regulated ribonuclease L (RNaseL), IFIT1, and viperin facilitate RNA degradation, inhibit translation, and disrupt egress steps [9,12]. On the other hand, (+)RNA viruses have also evolved multiple immune evasion strategies to counteract host defenses. These include direct cleavage or degradation of key signaling proteins such as MAVS, TBK1, or STATs by viral proteases, interference with PRR activation or localization, inhibition of ISGF3 formation, and modulation of host gene expression to limit ISG output [11,16–18]. In addition, minor alterations in the cellular environments or virus–host interactions can influence the balance between viral proliferation and host immune responses. A similar trade-off between the host and the virus has been suggested to explain the markedly different clinical outcomes observed in individuals with minor variations in their immune responses to the same virus [19,20]. Therefore, understanding the molecular-level dynamical interactions that shape infections is essential [21,22]. Leveraging the knowledge of critical stages in feedback networks that define host-virus interactions can help us devise therapeutic interventions with improved efficacy.

Given the complexity of these networks, mathematical models trying to capture the underlying behavior have focused on specific aspects of host-virus interactions with varying levels of detail. Several of these models on the viral life cycle [23–27] and the immune signaling pathways [15,28–30] reproduce experimental observations and recapitulate the dynamics of viral and cellular processes accurately. For example, Dahari *et al.* [24] predicted replicase binding bias for the double-stranded RNA intermediate for the HCV life cycle, whereas more recent virus life cycle models [23,25,26] have identified bottlenecks that can be exploited for antiviral treatments. Similarly, mathematical models of innate immune signaling have elucidated key features of the JAK-STAT and RIG-I pathways that modulate IFN responses and demonstrate signatures of IFN desensitisation [15,28,29]. However, most current models often isolate the viral life cycle or immune response, and the development of models that capture the dynamic interplay of host and viral factors in immune-competent cells has been challenging. While there is growing interest in understanding the viral dynamics within the context of innate immune signaling, most studies employ a simplified and coarse-grained approach [31–38]. Alternative systems biology approaches rely on protein–protein interaction (PPI) networks to reconstruct molecular interactions and gene regulatory circuits. By mapping the regulatory interactions, signaling PRRs, downstream effectors, and interferon-stimulated genes (ISGs), key regulatory hubs and the broad architecture of innate immune signaling during viral infection have been elucidated [39–41]. However, these approaches often treat viral life cycle or innate immune signaling as a static or external parameter, rather than dynamically interacting and co-evolving processes. Consequently, the disruptive action of viral factors on specific immune nodes, such as cleavage of PRR or their adaptor proteins [16,18] or ISG degradation [42], is underestimated.

In this study, we present a mechanistic model that integrates detailed mathematical representations of the (+)RNA viral life cycle and host innate immune signaling pathways, along with their dynamic coupling via specific molecular-level interactions. Here, we build on our previous generalized (+)RNA virus model that can accurately recapitulate the dynamics across different (+)RNA virus families [26]. We incorporate a comprehensive immune response network, which includes the key components of the RIG-I/IFN axis [43], enabling us to include the inductor and effector functions of the immune network explicitly. We show that cells respond to IFN in a dose-dependent manner and undergo desensitization under excessive exposure driven by the TYK and IFN receptors. Notably, viral life cycle dynamics are attenuated by the innate immune response, and virus counteractions on the host cell can negate this control. Similarly, distinguishing characteristics of the virus life cycle can determine the impact of the immune response in controlling virus growth. Clustering host factors based on their temporal expression suggests a stage-wise progression of infection through cell-state intermediates. Our integrated model also demonstrates a clear dichotomy in infection outcomes, a phenomenon that we hypothesize could be an underlying cause of cellular heterogeneity in viral infections [32,44–48]. Counteracting effects defined by the efficiency of virus-induced immune suppression and the strength of antiviral activity of the innate immune system determine this ‘all-or-none’ infection behavior. We identify that the kinetics of viral replication and translation, along with the expression of ISG, are key modulators of the transition between these two completely opposite infection states. Building on our understanding of the IFN dynamics, we propose the prophylactic potential of IFN in suppressing infection and demonstrate how co-targeting viral replication or immune suppression mechanisms can enhance its efficacy. Overall, this work underscores how quantitative systems-level modeling can assist in dissecting virus–host dynamics and offer predictive insights for antiviral strategy.

## Results

### A cellular model of virus-host innate immune interactions

**Fig 1:**
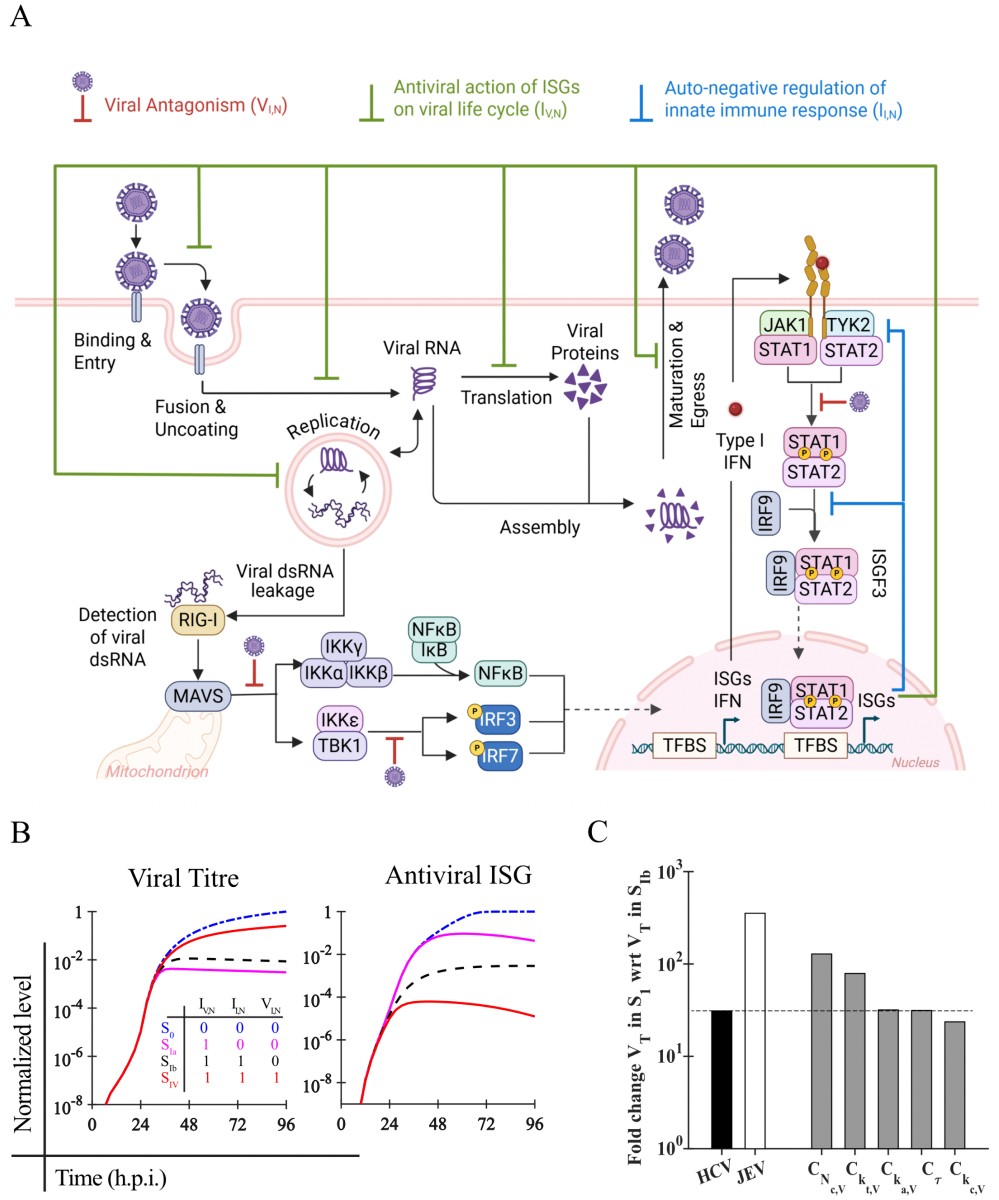
Integrated mathematical model of the viral life cycle with innate immune pathways. **A** Schematic representation of the intracellular model coupling the (+)RNA virus life cycle with host innate immune signaling. Viral infection begins with the extracellular virions (*V*_0_) binding and entering the host cell, followed by uncoating and release of the viral RNA. This RNA undergoes translation to produce structural ($P_{S}$) and non-structural proteins ($P_{NS}$), and replication via a double-stranded RNA intermediate ($RC_{CM}$), often sequestered within membrane compartments to evade immune detection. Leakage of replication intermediates into the cytoplasm can trigger recognition by pattern recognition receptors like RIG-I, activating the MAVS and downstream transcription factors such as IRF3/7 and NFκB. This results in the production and extracellular release of type I interferons. Extracellular IFN-I binds to its receptor, activating the JAK-STAT signaling cascade via STAT phosphorylation, which induces the transcription of interferon-stimulated genes (ISGs). ISGs can either suppress the viral life cycle (*ISGav*, strength given by $I_{V,N}$; green repression T-shaped arrows) or auto-regulate the innate immune pathway (*ISGn*, strength parameterized by $I_{I,N}$; blue repression T-shaped arrows). Viral antagonism, particularly via non-structural proteins, can suppress the activity of antiviral ISGs (suppression strength given by $V_{I,N}$; red repression T-shaped arrows). Created in BioRender. K, R. (2026) https://BioRender.com/9v006fh **B** Viral (left) and ISG (right) dynamics under the influence of various virus-immune interactions, normalized to 96 h-point levels without virus-immune interactions (*S*_0_: $I_{V,N}=I_{I,N}=V_{I,N}=0$, blue). The red line depicts dynamics under the action of viral antagonism and immune suppression along with negative auto-regulation ($S_{IV}$: $I_{V,N}=I_{I,N}=V_{I,N}=1$). Dynamics with immune activation with antiviral response alone ($S_{Ia}$: $I_{V,N}=1$, $I_{I,N}=V_{I,N}=0$, magenta) and host immune activation with negative autoregulation but no virus mediated immune suppression ($S_{Ib}$: $I_{V,N}=I_{I,N}=1$ and $V_{I,N}=0$, black) is shown for comparison. **C** Comparison of fold reduction in total viral load ($V_{T}$) between the $S_{Ib}$ case and various viruses with the entire virus-immune interaction network ($S_{IV}$) is shown; HCV (black bar), JEV (white bar), and a few hypothetical viruses (grey bars). All the model parameter values of the hypothetical virus, $C_{\theta }$, are based on HCV, except for θ, which is representative of JEV. θ virus variants were limited to $N_{C,V},k_{t,V},k_{a,V},\tau \text{ and }k_{c,V}$ since HCV and JEV primarily differ in these parameters.

We present a comprehensive mathematical model that provides an integrated framework for studying virus-innate immune interactions at the single-cell level. Building on two foundational models that individually describe the viral dynamics and immune response, [26,43] respectively, our model describes the combined dynamics of the virus and host immune response in an infected immune cell (Fig 1A). Previously, we had developed a detailed mathematical representation of (+)RNA virus life cycle that describes the intracellular dynamics of (+)RNA viral constituents, including the effects of vesicular compartment formation, viral mutations, and host cell variability [26]. Although this original viral dynamics model provided a quantitative framework for examining viral replication and growth, it lacked the cellular innate immune response. To address this, we have now incorporated the major immune cascade dynamics (inspired from Burkart *et al.* [43]), which models the RIG-I mediated detection of viral dsRNA and downstream signaling, and the JAK-STAT pathways. Our model, consisting of 77 equations, captures the essential nonlinear interactions between viral replication and innate immune signaling while remaining computationally tractable. To achieve this, we introduce a set of strategic simplifications that retain the principal regulatory motifs without attempting to reconstruct the full biochemical intricacy of the system. To maintain model tractability, we consolidated upstream sensing of RNA viruses into a unified dsRNA–RIG-I module, modeling immune detection exclusively through this primary cytosolic axis [49,50]. Negative regulation was similarly abstracted into a uniform feedback term. While omitting cell-type-specific modulators, these simplifications preserve the essential feedforward activation and delayed negative regulation inherent to innate immune signaling, enabling a precise characterization of the kinetic interplay between viral replication and RIG-I-mediated induction.

The integration of viral and immune dynamics was achieved by identifying the mechanisms that links them - (a) through viral dsRNA detection that facilitates RIG-I activation of the innate immune cascade, and (b) by detailing the reciprocal interactions between immune response effectors and the viral elements (Fig 1A). During infection, viral double-stranded RNA (dsRNA, the replication intermediate) accumulates in the cytoplasm as it leaks from the replication compartments [17,51,52] (equation 4). This cytoplasmic dsRNA becomes accessible to RIG-I, enabling its detection and subsequent activation of the innate immune response. In parallel, ISGs mount a robust and multifaceted defense by targeting viral entry, replication, translation, assembly, and release [9,12,53]. To account for these immune responses, we employ a Hill-function-based repression model to represent the ISG-mediated inhibition of these viral processes (Fig 1A, equations 1, 2, 3, 4; also S1 Text equations- S3, S4, S5, S6, S7, S9, S10, and S11). Apart from the effect of innate immune response on viral dynamics, we also account for how viral proteins actively interfere with the innate immune response by targeting key components of the signaling pathways. To model this interference, we introduce viral protein-mediated repression of signal propagation via MAVS, IKKϵ-TBK1, and the STAT1/STAT2 receptor complexes [54–56] (Fig 1A, S1 Text equations- S14, S21, S23, S44, and S46). Finally, we incorporate the regulation of IRF7 (equations 5 and 6), a master transcriptional regulator of type I interferons (esp. IFNα) previously overlooked in earlier models. IRF7 plays a critical role in amplifying IFNα responses through a positive feedback loop mediated by JAK-STAT signaling, where initial IFNβ production (mediated by IRF3) induces IRF7 expression (details provided in Methods). To precisely capture the dynamics of the cellular immune response, innate immune parameters are adapted from prior studies that estimate them using experiments with synthetic 5’ppp-dsRNA or IFN to stimulate cells [15,43]. Virus-specific parameters for HCV and JEV are obtained from our earlier work that reconciles experimental data of viral protein and RNA dynamics with viral life cycle dynamics [26].

To investigate the broad impact of the bidirectional virus-host interactions on infection progression, we first employ an aggregated parameter approach to examine the viral-immune dynamics in our comprehensive integrated model. For this purpose, we introduced two lumped tuning parameters: $I_{V,N}$, representing the strength of immune-mediated suppression of the viral processes (equations 1, 2, 3, 4; also S1 Text equations- S3, S4, S5, S6, S7, S9, S10, and S11; see Methods), and $V_{I,N}$, capturing the extent of viral protein-mediated suppression of the immune response (S1 Text equations- S14, S21, S23, S44, and S46; see Methods). Additionally, $I_{I,N}$ serves as a tuning parameter to modulate the negative feedback effect of anti-inflammatory ISGs on the JAK-STAT pathway (S1 Text equations- S37, S39, S41, S42, S43, and S44; see Methods). At the molecular level, these interactions influence different processes such as immune sensor activation, signaling, viral replication, and protein translation to varying extents; however, for analytical simplicity, we will assume a uniform regulatory effect throughout for each of the immune-mediated suppression and the viral antagonism processes. This approach ensures that the model remains interpretable while allowing us to explore the broader implications of these interactions on the infection outcomes. We begin by defining a baseline scenario (*S*_0_) devoid of counteracting interactions between the immune or viral components ($I_{V,N}=V_{I,N}=I_{I,N}=0$, Fig 1B). Under this condition, the model successfully reproduces the dynamics of Hepatitis C virus (HCV) proteins, consistent with experimental observations (S1 Fig) [25,26]. The JEV-specific parameters were likewise estimated previously in [26] against the data of Uchil and Satchidanandam (2003) [57], and are used here without modification. In this scenario, immune pathways are gradually activated, as evidenced by the increase in levels of interferon-stimulated genes (ISG). Next, we consider the scenario where the antiviral effects of the innate immune response are included ($S_{Ia}$: $I_{V,N}=1,I_{I,N}=V_{I,N}=0$). Here, the viral infection is substantially disrupted, and the ensuing reduction in immune activation reflects a resolution phase driven by the absence of sustained viral stimulus. In the subsequent scenario, we incorporate the negative self-regulation of the innate immune pathway ($S_{Ib}$: $I_{V,N}=I_{I,N}=1;V_{I,N}=0$). This results in a dampened immune response, leading to a less pronounced reduction in viral load compared to $S_{Ia}$. Finally, we examine the complete system by including the virus-mediated suppression of the innate immune pathways with the combined action of the immune pathways ($S_{IV}$: $I_{V,N}=I_{I,N}=V_{I,N}=1$). In this scenario, the immune response is further suppressed, as indicated by a significant reduction in ISG levels. In fact, 96 h-point ISG expression in $S_{IV}$ is approximately five orders of magnitude lower than in the baseline scenario (*S*_0_), suggesting strong repression of the immune response due to virus interference. However, there is only a modest change in the level of virus growth across the two scenarios (Fig 1B). Notably, compared to $S_{Ia}$, the complete system $S_{IV}$ exhibits a substantially higher viral load alongside a marked further suppression of ISG levels, underscoring the dominant role of virus-mediated immune antagonism in tipping the balance from viral clearance toward persistent infection. To further assess the robustness of the model, we examined how the qualitative dynamics of viral titre change across a range of multiplicities of infection MOI = 1.2 and MOI = 4.9 (S2 Fig). Across both conditions, the four scenarios (*S*_0_, $S_{Ia}$, $S_{Ib}$, $S_{IV}$) reproduce qualitatively similar ordering and trends in normalized viral titre trajectories, confirming that the interplay between viral replication and immune suppression is robust to the choice of initial inoculum.

By adjusting the model parameter values (S3 Table) to reflect the dynamics of Japanese Encephalitis Virus (JEV) [26], the system predicts kinetics that are qualitatively similar to those observed for HCV (S3 Fig). However, virus-specific differences emerge when comparing the values at the end of the simulation window across different scenarios and in the temporal dynamics of the immune response, particularly ISG induction, where $S_{Ib}$ displays distinct kinetics between HCV and JEV, with JEV showing a more rapid rise and sustained high ISG levels, in contrast to the attenuated ISG response observed in HCV. The immune-mediated suppression of viral infection, as indicated by the 96 h-point viral load ratio $S_{IV}/S_{Ib}$, is markedly higher for JEV (≈350) compared to HCV (≈30), highlighting a stronger suppression effect on JEV than HCV (Fig 1C). We hypothesize that this disparity arises from key differences in the viral life cycle parameters of JEV. Notably, JEV exhibits a faster viral translation rate (higher $k_{t,V}$) and larger number of replication complexes (higher $N_{C,V}$) compared to HCV. These factors drive a more rapid and substantial accumulation of replication intermediates, which in turn facilitates earlier and stronger activation of the immune response. Consequently, when the antiviral effects of the immune response are incorporated ($S_{IV}$), the reduction in viral load is significantly greater for JEV than for HCV. This highlights the critical role of viral replication dynamics in shaping the interplay between viral persistence and immune suppression.

To validate this hypothesis, we constructed a panel of hypothetical viral strains, each retaining the characteristics of HCV except for a single substituted feature that is derived from JEV (Fig 1C). For example, the parameter τ, parameterizing the timescale of replication-compartment formation, is 2-fold smaller in JEV than in HCV. Reducing τ to the JEV-like value in HCV yields a chimeric virus with faster compartment formation and earlier exposure of cytoplasmic dsRNA. In scenario ($S_{IV}$), the chimeric strain $C_{N_{C,V}}$, incorporating JEV-like carrying capacity ($N_{C,V}$), exhibits the strongest suppression of viral load relative to the baseline scenario ($S_{Ib}$), with reductions ≈150-fold and closely recapitulating JEV-like behavior. The strain $C_{k_{t,V}}$, incorporating a JEV-like translation rate ($k_{t,V}$), also shows enhanced suppression, albeit to a lesser extent, indicating a secondary contribution of translation dynamics to immune-mediated control. In contrast, chimeras modifying other parameters, including the replication-compartment formation timescale ($C_{\tau }$), compartmentalized replication intermediates formation rate ($C_{k_{c,V}}$), and rate of egress ($C_{k_{a,V}}$), exhibit comparatively modest changes in viral suppression and largely retain HCV-like behavior, with reductions typically ≈30-fold. Together, these results indicate that increasing the number of replication complexes is the dominant factor driving enhanced immune-mediated suppression, with faster viral translation providing an additional, but secondary, contribution. Mechanistically, this likely reflects more rapid accumulation of viral products, leading to earlier immune activation and more effective antiviral responses, consistent with observations in other systems [58].

### Reciprocal negative feedback between viral and immune processes shapes the viral-immune transition boundary

Experimental studies have demonstrated that early and robust innate immune responses, particularly those mediated by interferon-stimulated genes (ISGs), are essential for limiting viral replication and achieving viral clearance [59,60]. In contrast, delayed or dysregulated immune responses, often resulting from viral immune evasion strategies, can lead to immune escape and prolonged infection [61–64]. We argue that infection outcomes are governed by the dynamic titration of viral antagonism against host immune strength, both of which fluctuate along a biological continuum shaped by viral load, replication kinetics, and host-specific cellular or genetic contexts [65–68]. For example, different viral strains can differ in their ability to produce immune antagonists or stimulate ISG expression, while host immune activity is modulated by polymorphisms in interferon signaling components, ISG expression profiles, and metabolic or stress conditions. Motivated by these biological complexities, we investigated whether our virus-immune model is capable of capturing the dichotomy in infection outcomes and identifying the key cellular and viral features that govern the balance between viral clearance and immune escape.

**Fig 2:**
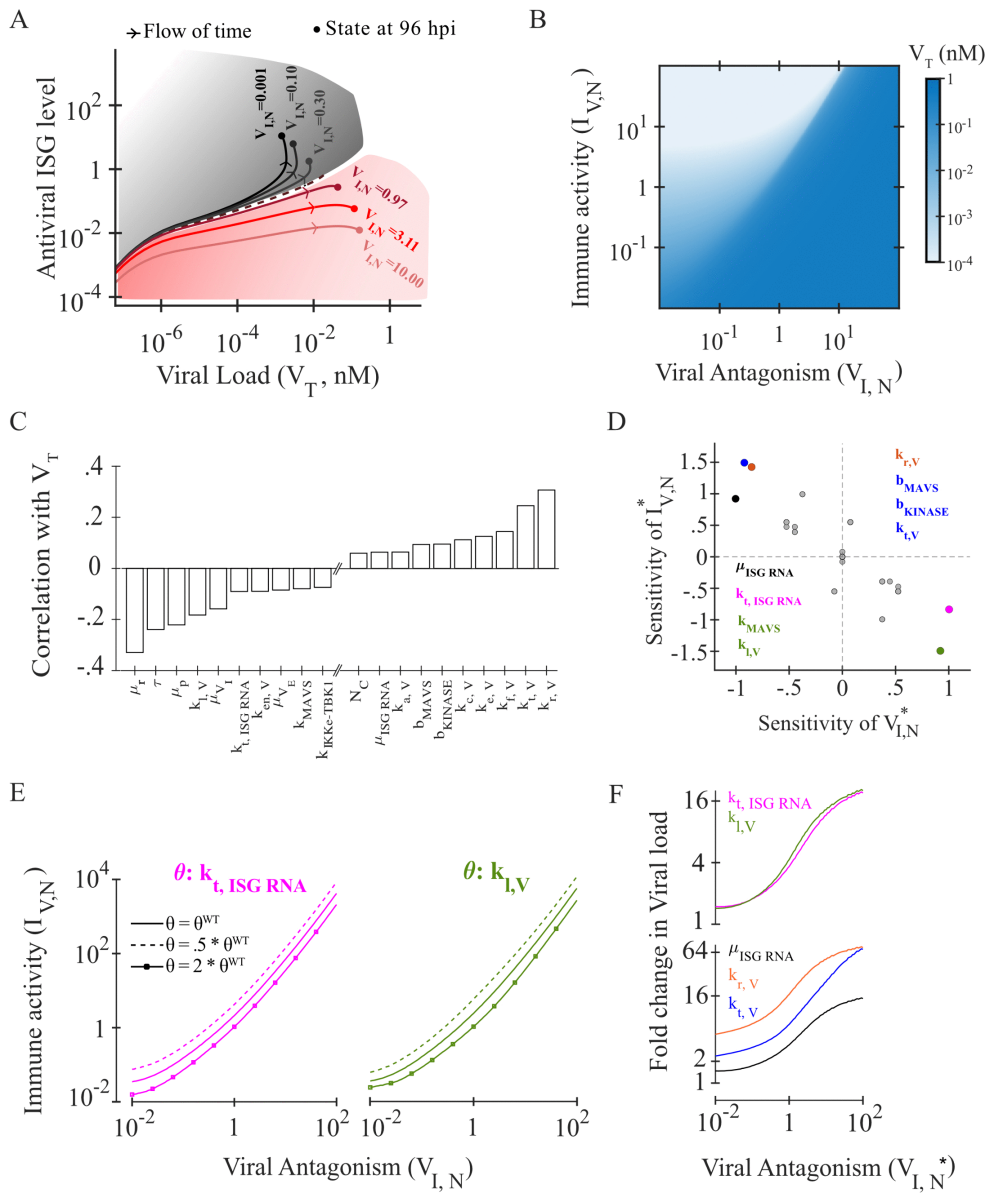
Virus-immune transition boundary and sensitivity analysis reveal critical regimes in infection. **A** The phase plot of viral load and antiviral ISG levels during hepatitis C virus infection is shown for various strengths of viral antagonism ($V_{I,N}$). Each curve shows the trajectory of the system in the $\left(V_{T},\;ISG\right)$ state space, with arrows indicating the direction of time from the initial state at 0 h.p.i to the state reached by 96 h.p.i (circles). The system shifts from a ‘virus-high, ISG-low’ state (pink region) to a ‘virus-low, ISG-high’ state (grey region) as $V_{I,N}$ increases, with the clear transition at $V_{I,N}$ = $V_{I,N}^{*}$ = 0.58 represented by the dashed line. **B** The viral load ($V_{T}$ at 96 h.p.i) HCV is shown as a function of $V_{I,N}$ and $I_{V,N}$. **C** Correlation coefficients between viral load ($V_{T}$) and individual parameters near the *VITB* boundary show how a parameter influences the viral load at the end of the simulation window, thus identifying antiviral and proviral parameters. **D** Parameter sensitivity indices for $I_{V,N}^{*}$ and $V_{I,N}^{*}$ evaluated at the infection threshold point: ($I_{V,N}^{*}$, $V_{I,N}^{*}$) = (1, 0.58) are negatively correlated. Colored dots denote model parameters with threshold sensitivity score > 1 (see Methods). **E** The *VITB* boundary for HCV infection systems is shown for different values of $k_{t,ISGRNA}$ (left) and $k_{l,V}$ (right). The solid line, dashed line, and the solid line with square symbols show the *VITB* boundary when the parameter is unperturbed, reduced by half, and doubled, respectively. **F** Fold change in the HCV viral load at 96 h-point when the corresponding parameter values were (top panel) halved (magenta-$k_{t,ISGRNA}$, green-$k_{l,V}$, or (bottom panel) doubled (black-$\mu_{ISGRNA}$, blue-$k_{t,V}$, and orange-$k_{r,V}$) along the *VITB* boundary.

The strength of viral antagonism ($V_{I,N}$) determines distinct immune response dynamics. At low $V_{I,N}$ values, ISG levels remain high and the viral loads remain low, indicating robust immune activation and effective viral clearance. However, as $V_{I,N}$ increases, ISG levels are progressively suppressed, particularly at higher viral loads, reflecting the dominance of viral antagonism and the transition to immune escape (Fig 2A). For HCV, the infection outcome switches from a ‘virus-low, ISG-high’ state to a ‘virus-high, ISG-low’ state (Fig 2A) as $V_{I,N}$ surpasses 0.58, suggesting that HCV can overcome immune control above this critical level. Further, a threshold viral load is evident below which ISG levels remain low, regardless of $V_{I,N}$, suggesting that such low viral loads are insufficient to trigger strong immune activation.

We can now evaluate how virus infection is established as a function of the strength of the two key regulatory interactions in our model: the immune-mediated viral suppression (by varying $I_{V,N}$) and the viral antagonism of the immune response (by varying $V_{I,N}$). To delineate the transition between immune control and viral escape, we systematically varied the lumped parameters, $I_{V,N}$ and $V_{I,N}$ while maintaining the reciprocal parameter at a fixed value. By evaluating the total viral output ($V_{T}$), at the end of the simulation window, we identified the critical thresholds, $I_{V,N}^{*}$ and $V_{I,N}^{*}$, which represent the system’s points of maximal sensitivity. Specifically, these thresholds were defined by the peak of the first-order numerical derivative of $\log_{10}(V_{T})$. For instance, $I_{V,N}^{*}$ was determined by locating the sharpest transition in viral titre as a function of immune activity, while an analogous procedure was employed to derive $V_{I,N}^{*}$ from the maximal slope of the viral output curve with respect to viral antagonism. Mapping $V_{T}$ across the $I_{V,N}$ and $V_{I,N}$ parameter space reveals a transition boundary separating bimodal outcomes: (a) a low-virus regime, where high $I_{V,N}$ and low $V_{I,N}$ result in effective immune clearance, and (b) a high-virus regime, where low $I_{V,N}$ and high $V_{I,N}$ leads to immune escape (Fig 2B). The locus of critical points ($I_{V,N}^{*}$, $V_{I,N}^{*}$) defines the Viral-Immune Transition Boundary (*VITB*) that captures the tipping point between innate immune control and virus immune escape and crossing precipitates a rapid switch between virus-low/ISG-high and virus-high/ISG-low states. Near this boundary, the infection outcome is characterized by extreme sensitivity, where minor variations in the virus-host balance fundamentally dictate whether an infection resolves or persists. There is a marked positive correlation between the critical values of $I_{V,N}^{*}$ (immune activity) and $V_{I,N}^{*}$ (viral antagonism), indicating a balancing interaction: as viral antagonism intensifies, enhanced immune activity is necessary to counteract it. Overall, the *VITB* boundary emphasizes the nonlinear dynamics of the virus-immune system, where robust immune activation (high ISG levels) can suppress viral replication, but strong viral antagonism can overwhelm the immune response, leading to persistent infection.

An analogous trend is detected for JEV (S5 Fig). However, with the same level of innate immune activity ($I_{V,N}=1$), JEV requires a lower level of antagonism to evade immune control ($V_{I,N}^{*}$ = 0.16) compared to HCV ($V_{I,N}^{*}$ = 0.58). This is consistent with JEV being more strongly suppressed by the innate immune response than HCV in the absence of antagonism (Fig 1C), once we note that $V_{I,N}$ sets a *per-protein* efficacy while the net antagonistic burden scales with total antagonist abundance. JEV’s higher translation rate ($k_{t,V}$) – identified above as a primary driver of its enhanced dsRNA-mediated immune activation – also accelerates accumulation of viral antagonists, so a smaller $V_{I,N}^{*}$ suffices for immune evasion. HCV’s slower protein synthesis [26] correspondingly demands higher *per-protein* efficacy to reach a comparable burden.

Our initial approach involved broad perturbations across multiple interactions (varying $I_{V,N}$ and $V_{I,N}$) rather than specific molecular-level processes. However, such simultaneous perturbations are unlikely to occur in natural systems. A more plausible scenario is that the fitness of the virus or the immune response is influenced by specific changes in a single or a small subset of parameters. To explore this, we investigated whether the Virus-Immune Boundary (*VITB*) emerges naturally from the virus-immune interaction network under more realistic conditions, where only a limited set of parameters change for different viral strains or cell types. Notably, similar sharp transitions between viral clearance and escape states with distinct *VITB* boundaries were observed for HCV when we varied specific model parameters, such as $k_{t,V}$, $k_{r,V}$, $k_{l,V}$ and $k_{t,\;ISG\;RNA}$ (S4 Fig). This sharp transition at the *VITB* boundary provides a unique opportunity to dissect the relative contributions of viral and host processes to infection outcomes without the need to simulate the full system dynamics under individual parameter variations.

By leveraging the system’s heightened sensitivity near the *VITB* boundary, we can identify key parameters that strongly influence infection outcome. The analysis in Fig 2C illustrates how this approach enables the identification of critical drivers of the infection threshold (at $V_{I,N}^{*}$ = 0.58, $I_{V,N}^{*}$ = 1 for HCV). Specifically, we simulate the infection outcomes using a set of 25,000 parameter combinations, where each parameter is sampled from a log-uniform range spanning two orders of magnitude (0.1× to 10× the nominal value) (see S2 and S4 Tables, and Methods). This approach allows us to recover a bimodal distribution in viral loads (S6 Fig) that highlights the pronounced dichotomy in infection states near the *VITB* boundary. Approximately 60% of the parameter combinations lead to viral clearance, while 40% result in high viral load (>1 nM). Notably, neither outcome strongly dominates the parameter ensemble, placing our model in a regime consistent with the cell-to-cell variability in infection outcome reported in isogenic cell populations infected with (+)RNA viruses [32,45,48,69,70]. Correlation between the sampled parameter values and the resulting viral load helps us in identifying proviral and antiviral factors (Fig 2C). We find that the degradation rate of viral RNA ($\mu_{r,V}$), proteins ($\mu_{p,V}$), internalized virus ($\mu_{V_{I}}$) or extracellular virus particles ($\mu_{V,V}$) is strongly correlated with reduced viral load, highlighting their obvious role as critical parameters controlling viral growth. We also find that time constant for development of replication compartments is also strongly correlated with reduced viral load. Similarly, parameters that enhance viral processes—such as viral translation ($k_{t,V}$), replication ($k_{r,V}$), export of viral (+)RNA from replication compartments ($k_{e,V}$), fusion of viral and endosomal membranes ($k_{f,V}$), or the carrying capacity of compartmentalized replication complexes ($N_{C,V}$) - are positively correlated with higher viral loads. On the other hand, enhancing immune detection, for example, via increasing the leakage of compartmentalized viral dsRNA into the cytoplasm ($k_{l,V}$), leads to a reduction in viral load. Among the host cell properties, potent immune signaling and activation can similarly reduce virus production. For instance, enhanced phosphorylation of TBK1 or IKKϵ ($k_{IKKe-TBK1}$), or increased activation rates of MAVS ($k_{MAVS}$) or upregulation of host protein ($k_{t,\;ISG\;RNA}$) can considerably decrease viral load. Conversely, when immune factors undergo rapid removal due to enhanced degradation or deactivation, we observe increase in viral load suggesting diminished innate immunity. This is exemplified for the case of ISG mRNA ($\mu_{ISG\;RNA}$) degradation, and dephosphorylation of MAVS ($b_{MAVS}$), or kinases ($b_{KINASE}$).

To further dissect the mechanisms governing the balance between immune and viral interplay, we performed an infection threshold sensitivity analysis to quantify how changes in crucial viral and host parameters affect the system’s tipping point (details in Methods). A distinct inverse correlation is evident in how various parameters affect $I_{V,N}^{*}$ and $V_{I,N}^{*}$, underscoring the antagonistic nature of these two sets of interactions (Fig 2D). Interestingly, some molecular-level processes stand out as more proviral or antiviral than the others. Among the viral processes, parameters that directly influence protein production, such as translation rate $k_{t,V}$, or indirectly by boosting RNA synthesis, like replication rate $k_{r,V}$, exhibit the strongest negative influence on $V_{I,N}^{*}$ (S7 Fig). For instance in the case of HCV, a + 10% increase in $k_{r,V}$, reduces the viral antagonism ($V_{I,N}^{*}$) required for virus escape by 9.4% (from 0.58 to 0.525) for a fixed $I_{V,N}^{*}$ (= 1). Thus, enhanced viral replication and translation aid in efficient immune escape. Regarding the immune response, mechanisms that enhance ISG activity ($\mu_{ISG\;RNA}$ and $k_{t,\;ISG\;RNA}$, $k_{MAVS}$) reduce the required strength of the immune response to control the viral proliferation. Conversely, we observe that enhanced leakage of compartmentalized viral dsRNA into the cytoplasm ($k_{l,V}$) can decrease $I_{V,N}^{*}$. For instance, at the *VITB* boundary, when the rate of leakage of compartmentalized dsRNA is increased by +10%, the antiviral activity ($I_{V,N}^{*}$) required for immune control is decreased by 4.5% (from 1 to 0.95) for HCV. This provides additional evidence that elevated $k_{l,V}$ may trigger earlier and more abundant production of immune agonists (dsRNA), which support the immune response (Fig 2C). In case of JEV, swift formation of replication compartments (small τ) result in rapid dsRNA accumulation. This triggers a potent immune activation and requiring only mild immune response to suppress it. Conversely, in HCV infection, slower dsRNA production postpones immune activation, necessitating stronger immune suppression to control infection.

We next extend our infection threshold sensitivity analysis to investigate such virus-immune tipping point dependence on various critical cellular and viral processes (identified in Fig 2D) along the entire *VITB* boundary. Figs 2E, S8, and S9 illustrates how a twofold increase or a 50% decrease in ISG translation efficiency or viral dsRNA leakage influences the *VITB* boundary across the entire range of viral antagonism ($V_{I,N}^{*}$) and immune activity ($I_{V,N}^{*}$) for HCV. Higher ISG RNA translation rate ($k_{t,\;ISG\;RNA}$) requires lower immune activity to counteract viral antagonism (downward shift in *VITB* boundary) and achieving viral clearance, while reducing it leads to weakening of immune control (upward shift in *VITB* boundary) (Fig 2E left). We observe that a two-fold decrease in ISG translation ($k_{t,\;ISG\;RNA}$) leads to a 3–16 fold increase in HCV viral load at different points on the transition boundary (Fig 2F top). Effectively, an increase in $k_{t,\;ISG\;RNA}$ leads to a downward shift of the *VITB* boundary in the $I_{V,N}-V_{I,N}$ space, indicating that change in the host ISG RNA translation rate consistently impact the system’s behavior uniformly along the transition boundary. This trend is also observed for a two-fold higher ISG mRNA degradation rate that results in an ∼ 3–16 fold increase in viral load at different points along the *VITB* boundary.

Unlike host-mediated processes, perturbations in viral processes, such as replication ($k_{r,V}$), or translation ($k_{t,V}$) influence system behavior in a regime-dependent manner across the transition boundary interface (Fig 2E, right; S8 Fig). At high levels of both viral antagonism and immune activity, increasing the rate of translation ($k_{t,V}$) shifts the *VITB* boundary upward, requiring stronger immune activity ($I_{V,N}$) to counteract viral antagonism ($V_{I,N}$) and reduced levels of viral proteins makes it easier for the immune system to suppress viral replication. Similar asymmetric changes in the *VITB* are also observed when other virus-mediated proviral processes are impeded (S9 Fig). Such dependence of these viral processes even influences the viral load levels in a regime-dependent manner along the *VITB* boundary (Fig 2F). When virus–immune interactions are weak (low $I_{V,N}$ and $V_{I,N}$), viral load ($V_{T}$) is largely insensitive to changes in $k_{r,V}$, or $k_{t,V}$ (Fig 2F). However, in the strongly interacting regime of the *VITB* boundary, small changes in the proviral factors have disproportionately large effects. For instance, along the boundary at high $V_{I,N}$ (= 10), a two-fold increase in $k_{r,V}$ or $k_{t,V}$ yields ∼60-fold and ∼50-fold increases in $V_{T}$, respectively (Fig 2F). Comparable trends for JEV (S8, S9, and S10 Figs) suggest this regime-dependent sensitivity is a general feature of (+)RNA virus-host immune interactions.

These regime-dependent effects of viral parameters can be explained by two factors: (a) the dual role of viral dsRNA as both a viral replication intermediate and an immune activator, and (b) the double-negative feedback loop between viral antagonism ($V_{I,N}$) and immune suppression of viral replication ($I_{V,N}$). Given the dual role of viral dsRNA, changes in viral processes, like viral replication, translation, and replication compartmentalization, that alter its levels exert both pro-viral and antiviral influences. These counter-influences tend to balance each other at the *VITB* interface. When the negative feedback interaction strengths are high, strong mutual antagonism amplifies even minor imbalances, producing pronounced shifts in infection dynamics and outcome (Fig 2E and 2F).

### Dynamic phases of immune response during infection progression

Deciphering the temporal dynamics of immune responses during infection is essential for identifying key regulatory mechanisms, critical immune checkpoints, and potential therapeutic targets. In complex biological systems like viral infections, immune factors and viral components interact over distinct temporal phases, each defined by specific molecular patterns. However, due to the vast array of immune components, monitoring the dynamics of all elements is impractical in experimental settings, and identifying the most informative markers poses a complex challenge. We can leverage our virus-immune response model to address this challenge.

**Fig 3:**
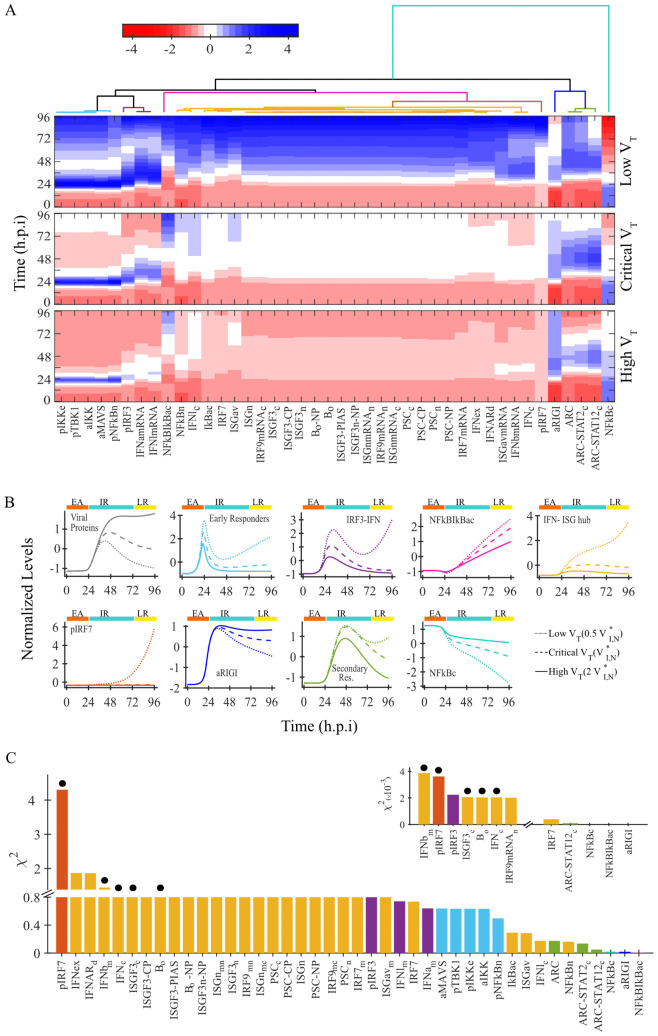
Dynamics of innate immune effectors under different infection outcomes. **A** Clustered z-normalized dynamics of immune pathway components under three viral-immune regimes: low $V_{T}$ ($V_{I,N}=0.5V_{I,N}^{*}$; top), critical $V_{T}$ ($V_{I,N}=V_{I,N}^{*}$; middle) and high $V_{T}$ ($V_{I,N}=2V_{I,N}^{*}$; bottom) are plotted. Distinct clusters are indicated by the color-coded dendrogram. **B** The panels show the dynamics of viral proteins (grey) and the mean temporal profiles of the immune pathway clusters identified in **A**, revealing their characteristic dynamic behavior under each regime. **C** The $\chi^{2}$ metric, quantifying the divergence between dynamics of immune factors across the low $V_{T}$ and the high $V_{T}$ regimes, is plotted (see Methods; bar edge colors reflect the cluster assignment). The inset shows the $\chi^{2}$ metric for the early phase (0–24 h). Dots indicate factors that show high divergence in both time periods.

Here, we investigate the temporal profiles of immune components and examine their temporal correlations to pinpoint effective indicators of cellular infection spread. To capture the immune system’s underlying dynamics, we examine its behavior under three distinct virus-immune regimes (Figs 3A, S11, S12, and S13): (a) at the *VITB* boundary ($V_{I,N}=V_{I,N}^{*}$), (b) viral clearance regime ($V_{I,N}=0.5V_{I,N}^{*}$), and (c) immune escape regime ($V_{I,N}=2V_{I,N}^{*}$). Variables exhibiting substantial change in their dynamics (coefficient of variation ≥0.05) were retained (see Methods), and their trajectories across all regimes were concatenated and z-normalized, enabling a unified comparison of temporal patterns. Hierarchical clustering of the z-normalized trajectories of immune factor dynamics across these regimes revealed eight distinct groups of dynamically co-regulated immune factors (Fig 3A). Each cluster exhibits unique temporal patterns that can be delineated into three major temporal phases of immune response during infection: an ‘early activation (EA)’ phase (0-24h), an ‘intermediate response (IR)’ phase (24–72 h), and a ‘late resolution (LR)’ phase (72–96 h)(Fig 3B).

In the ‘early activation’ phase, the newly formed viral dsRNA is detected by the RIG-I, triggering a sudden but transient increase in the levels of the “Early responders” cluster (Fig 3A and 3B). This group is dominated by the activated forms of antiviral signal transducers (aMAVS, pIKK, pTBK1, pIKKε, and pNF$\kappa {\text{B}}_{n}$). Their levels reach a peak around 12–24 hours, followed by a sharp decay. Overall, “Early responders” display similar initial transient dynamics in all three infection regimes. However, their induction level is inversely proportional to the severity of the infection, i.e., strong activation of the “Early responders” is associated with the viral clearance regime. Additionally, “Early responders” increase further in the resolution phase, likely due to reinforcement of immune signaling when initial immune responses are effective. Concurrently, the “IRF3–IFN” cluster — comprising pIRF3, IFNαmRNA, and IFNλmRNA — undergoes a similarly transient early peak followed by sharp decay. The “RIG-I Sentinel” cluster (aRIGI) is also engaged in this phase, exhibiting rapid sigmoid-like activation within the first 24 h that reflects prompt detection of cytoplasmic dsRNA by the pattern recognition machinery. The late-phase behaviour of both clusters is regime-dependent and is described below.

During the ‘intermediate response’ phase, as the viral replication is established and viral dsRNA increases, RIG-I activation increases. This initiates the activation of the “IFN-ISG transcriptional hub” (Fig 3A and 3B), which encompasses phosphorylated STATs, IRFs, and IFN/ISG mRNAs. These immune components exhibit a progressive increase in their levels as the infection advances, reaching their highest concentration during the ‘intermediate response’ phase. However, the increase was substantially repressed under high $V_{T}$ (immune escape regime), which can be attributed to the suppression of the signaling pathways by the viral proteins (Fig 1A). Conversely, when considering viral clearance characterized by low $V_{T}$, the elevation in levels during the resolution phase is significantly greater than in other regimes. This suggests robust reinforcement of pathways and effective antiviral transcriptional activity. In this phase, activation of the “Secondary responders”, comprising STAT-bound IFN receptor complexes (ARC, ARC-STAT2c, and ARC-STAT12c), plays a pivotal role in directing immune responses. This group exhibits a bell-shaped dynamic, rising to a peak around 36–48 h before gradually declining, potentially due to feedback inhibition and resource depletion. The peak was most pronounced under the viral clearance regime, and its attenuation at high $V_{T}$ is consistent with diminished upstream IFN-driven receptor engagement due to robust viral immune suppression. Activation of this complex thus represents a pivotal relay between IFN production in the IR phase and the transcriptional amplification that follows in late resolution.

In the ‘late resolution’ phase, the regime-dependent fates of the early-activated clusters become apparent. The “RIG-I Sentinel” (aRIGI) declines under low virus ($V_{T}$), consistent with clearance of the viral dsRNA stimulus, but remains persistently elevated under high virus despite impaired downstream signaling. Similarly, the “IRF3-IFN” cluster shows a secondary rise specifically under low infection (low $V_{T}$), reflecting renewed IRF3-driven transcription as infection resolves. Distinct from these is the “IRF7 Amplifier” (pIRF7), which emerges as the dominant factor of this phase: it remains near baseline through most of the infection before rising steeply after 72 h exclusively under low infection (low $V_{T}$), driven by accumulated IFN-mediated induction of IRF7 and its positive feedback activation. This late amplification is entirely absent under the immune escape regime.

NFκB dynamics during virus infection can be understood from the behaviour of the “$NF\kappa B_{c}$,” $pNF\kappa B_{n}$, and “NFκB–IκBα” forms (Fig 3A and 3B). Cytoplasmic $NF\kappa B_{c}$ forms a standalone cluster and is quickly depleted due to phosphorylation, nuclear import, and its capture by IκBα. This rapid drop highlights its role as the input pool for downstream NFκB signaling. As $NF\kappa B_{c}$ translocates and phosphorylates into $pNF\kappa B_{n}$, it induces IFNs and feedback regulator IκBα. The transient peak of “$pNF\kappa B_{n}$” is concomitant with “Early Responders” group dynamics, peaking around 12–24 hours followed by a sharp decay. The “NFκB-IκBα” is a regulatory module that constitutes a distinct cluster exhibiting a delayed but progressive monotone increase from approximately 24 h, consistent with its role as a feedback brake whose accumulation depends on prior NFκB-driven IκBα transcription. Under high $V_{T}$, this increase is attenuated, reflecting a partially active and inefficient NFκB cycle caused by viral suppression of the MAVS–IKK signaling pathway. However, under low viral load, the rise is more pronounced owing to enhanced signaling activity.

Taken together, our analysis reveals a hierarchical temporal cascade in the host antiviral response, wherein identified clusters reflect functionally distinct stages of innate immune signaling and effector activation. The clusters show significant sensitivity to viral load, with their rapid modulation highlighting crucial points where host detection and response pathways may succeed or fail. Notably, the early divergence of the “IFN-ISG transcriptional hub” and “pIRF7” in various infection regimes suggests these molecules can serve as early indicators of infection outcomes, even before clear phenotypic signs appear.

We employ a $\chi^{2}$ metric to compare the difference between high and low viral load scenarios (Fig 3C, see Methods). Phosphorylated IRF7, nascent IFN transcripts, and early ISGs are key factors in viral infection outcomes, particularly in the initial stages of infection, indicating that the immediate-early transcriptional circuit plays a crucial role in initiating effective antiviral states (Fig 3C-inset). Throughout the infection period, the persistent impact of IFN levels, phosphorylated IRF7, highlights the critical roles of strong downstream amplification in immune regulation (Fig 3C). These findings map the dynamics of viral antagonism directly onto distinct regulatory layers of the host response, illustrating how viral interference at both early detection and later transcriptional amplification stages can rewire immune trajectories and shape disease outcomes. Our model facilitates the understanding of the hierarchical and temporal structure of the antiviral response, identifying specific molecular clusters as crucial indicators of the infection progression.

### IFN Desensitization is Mediated by Receptor-Kinase Complex and TYK Dynamics

IFNs are widely used as therapeutic agents for the treatment of chronic viral infections, certain cancers, and autoimmune diseases, due to their potent antiviral and immunomodulatory effects [71,72]. However, their clinical efficacy is often limited by the phenomenon of desensitization, where repeated or prolonged IFN exposure leads to reduced cellular responsiveness [73]. This behavior is thought to arise from feedback inhibition and regulatory dynamics within the JAK-STAT signaling pathway [74–76].

**Fig 4:**
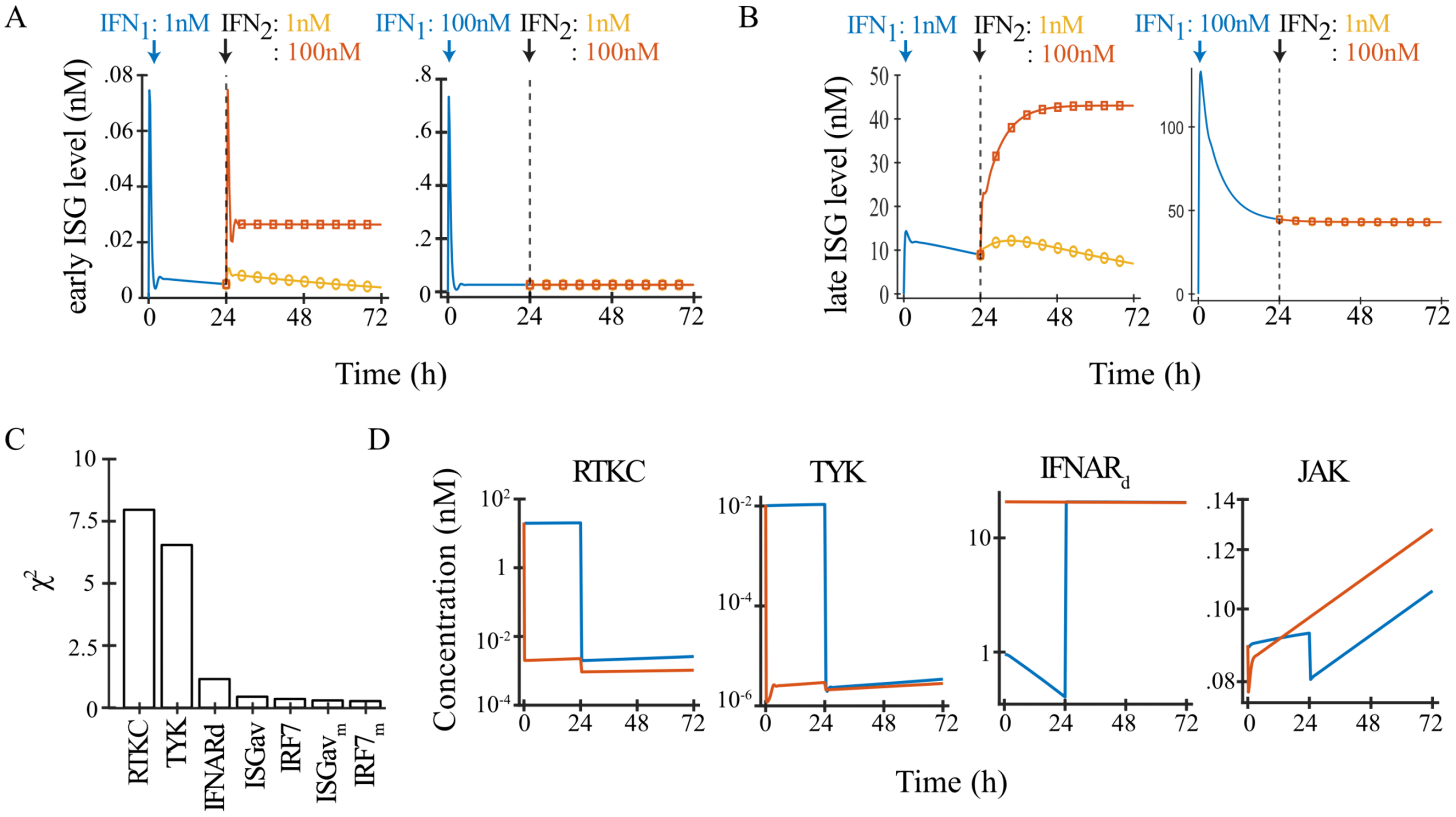
Dose-dependent IFN-desensitization. The kinetics of **A** an ISG transcript involved in immune suppression (early ISG, ISGn) and **B** an ISG transcript involved in viral suppression (late ISG, ISGav) are compared under distinct two-dose interferon (IFN) treatments. The second dose is administered 24 hours after the initial dose. The left panels represent Condition 1, where an initial low dose of 1 nM IFN is followed by either a low (1 nM, yellow line) or high (100 nM, orange line) second dose. The right panels represent Condition 2, where an initial high dose of 100 nM IFN is followed by either a low (1 nM, yellow line) or high (100 nM, orange line) second dose. **C** The $\chi^{2}$ values, representing deviations in the dynamics of JAK-STAT pathway components between the two IFN priming conditions, are plotted. The top-ranking species, which indicate model components most sensitive to the initial IFN dose, are highlighted. **D** Time-course dynamics of select model species with the two highest ($\chi^{2}$: RTKC, TYK) and two moderate ($\chi^{2}$: IFNARd, JAK) values are shown, illustrating IFN dose-dependent differences in system response.

Because our model incorporates key immune pathways that regulate the IFN response, it can capture the dose-dependent desensitization effect for a naive (uninfected) cell (see Methods). A high dose of IFN (100 nM) sharply induces ISGs responses with ‘ISGn mRNA’, an early transient negative regulator [15], and the ‘ISGav mRNA’, a late antiviral transcript [15], quickly rising to 0.75 nM and ≈ 140 nM, respectively (Fig 4A and 4B). This activation is brief, with both mRNA dropping significantly and stabilizing at approximately 0.01 nM and 60 nM within 24 hours. A second high (100 nM) IFN dose 24 hours later elicits no additional increase in ISGn or ISGav mRNA, signifying strong desensitization. Conversely, administering a high subsequent IFN dose (100 nM) following an initial low dose (1 nM) results in an increase in ISGn and ISGav, albeit it is modest compared to the substantial increase observed with a single high-dose (100 nM) of IFN. This signifies limited IFN desensitization consistent with experimental reports of dose- and time-dependent IFN desensitization [15,30].

Comparing these scenarios, most downstream signaling components remain similar at re-exposure, but key upstream regulators, particularly TYK2 and the IFN receptor complex (IFNAR1/2), show marked depletion after high-dose exposure (Fig 4C and 4D). This suggests that desensitization arises from the destabilization or inhibition of receptor-proximal components, as reported in experimental studies [15,74–77].

To further test this hypothesis, we simulated a two-dose IFN regimen (100 nM followed by 100 nM). We selectively restored the RTKC, TYK, and JAK levels before the second dose. Restoring the receptor-TYK2 complex (RTKC) partially recovered both ISGs expression, while restoring TYK2 alone had no effect (S14 Fig), indicating that negative feedback primarily targets the RTKC. Reintroducing JAK, whose levels remained unchanged across conditions, also had no impact on ISG expression. These results confirm that desensitization is driven by the depletion or inhibition of the receptor-kinase complex, which serves as the key regulator of downstream IFN signaling.

### Harnessing interferon and antiviral synergies to enhance viral suppression

**Fig 5:**
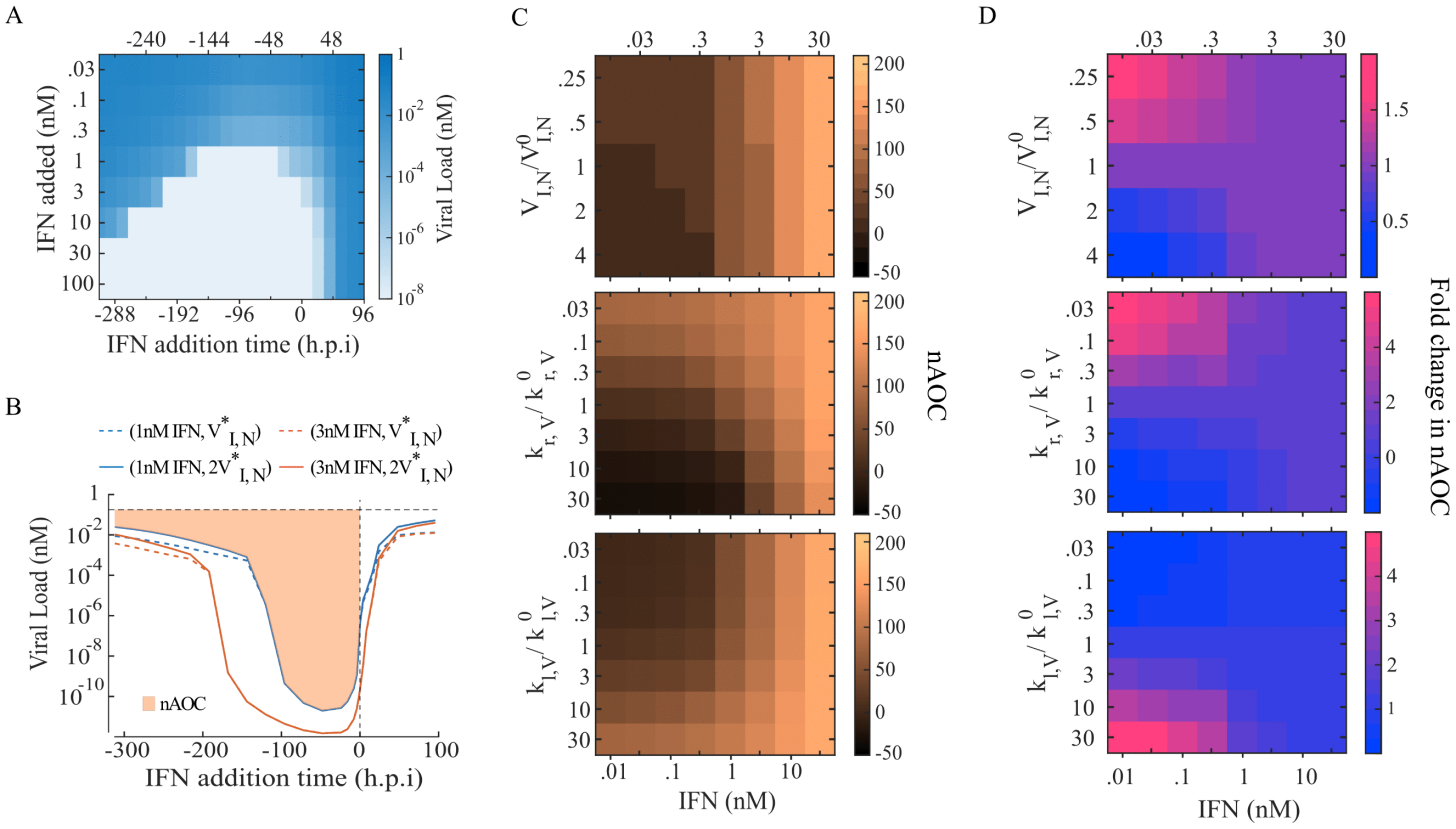
Effect of IFN dosage and timing on viral infection. **A** Heatmap showing the viral load (nM) as a function of the timing of IFN addition (hours post-infection, h.p.i.) and the IFN dosage (nM). Simulations were conducted under a virus-high regime, where the virus exhibits enhanced immune escape capabilities (virus-high regime; $V_{I,N}=2V_{I,N}^{*}$). **B** Viral load dynamics under IFN treatment for two IFN dosages (blue: 1 nM, red: 3 nM) and two levels of viral countermeasure efficiency (dotted lines: $V_{I,N}=V_{I,N}^{*}$; solid lines: $V_{I,N}=2V_{I,N}^{*}$) are shown. The prophylactic efficacy is quantified using the area over the curve (AOC), represented by the light copper shaded region. A higher AOC value corresponds to a stronger prophylactic effect, as it reflects a more substantial reduction in viral load. **C** Heatmaps illustrating the normalized AOC (nAOC) values as a function of IFN dosage (nM) and strength of key viral parameters: (top) viral countermeasure efficiency ($V_{I,N}$), (middle) viral replication rate ($k_{r,V}$) and (bottom) viral dsRNA leakage rate ($k_{l,V}$). Here, $k_{l,V}^{0}$, $k_{r,V}^{0}$, and $V_{I,N}^{0}$ represent the baseline or WT values and the ratios- $k_{l,V}/k_{l,V}^{0}$, $k_{r,V}/k_{r,V}^{0}$, and $V_{I,N}/V_{I,N}^{0}$, represent the change w.r.t to the WT values. **D** Heatmaps illustrating the fold change in normalized AOC (nAOC) as a function of IFN dosage (nM) and key viral parameters: (top) viral countermeasure efficiency ($V_{I,N}$), (middle) viral replication rate ($k_{r,V}$) and (bottom) dsRNA leakage rate $k_{l,V}$. Warmer colors (pink) indicate higher fold changes in nAOC, signifying improved prophylactic efficacy.

Building on our insight about IFN-mediated immune response regulation, we examined how interferon administration can modulate the outcome of viral infection. The timing and dosage of IFN treatment are suggested as critical factors influencing its efficacy [78–80]. Our model shows that IFN treatment markedly decreases viral load in a dose-dependent fashion when given before or shortly after infection (Fig 5A). The timing of administration significantly impacts treatment efficacy. Specifically, a low IFN dose (1 nM) given 1–7 days before infection yields an antiviral effect over 100 times greater than a high dose (>10 nM) applied a few hours post-infection (Fig 5A). We observe that IFN potency declines rapidly once replication is established, with inhibition strongest during the earliest stages of infection. This is consistent with studies showing that early replication and translation events represent a vulnerable bottleneck for viral control [78], that dengue virus is sensitive to IFN only before productive replication [79], and that SARS-CoV-2 becomes largely refractory to IFN following initial replication due to antagonism of the JAK–STAT pathway [80].

Prompted by this result, we examine the effectiveness of IFN as a prophylactic agent and its efficacy in combination with direct-acting antivirals (Fig 5C, 5D, and S15 Fig). To standardize comparisons across infection scenarios, we quantify antiviral efficacy by estimating the fold reduction in viral load integrated over the treatment duration (area-over-curve metric, AOC; Fig 5B). This area-over-curve metric, normalized against no IFN exposure (referred here as nAOC, see Methods), captures the time-of-addition-independent antiviral potency of the strategy, with higher nAOC scores indicating superior viral suppression. As anticipated, the AOC for low-dose IFN (1 nM; ≈60, Fig 5B, dashed blue) was significantly lower than that of a higher dose (3 nM; ≈89, Fig 5B, dashed red). Furthermore, the efficacy of 1 nM prophylactic IFN exposure was inversely correlated with viral antagonism ($V_{I,N}$). Specifically, the AOC decreased from ≈60 at $V_{I,N}$ = $V_{I,N}^{*}$ (Fig 5B, dashed blue) to ≈56 when antagonism was doubled ($V_{I,N}$ = 2$V_{I,N}^{*}$; Fig 5B, solid blue). These trends confirm that IFN-mediated suppression scales positively with dosage but is fundamentally constrained by the strength of viral immune evasion.

We next examined how direct-acting antivirals (DAAs), acting on distinct steps of the viral life cycle, influence the prophylactic efficacy of IFN. Specifically, we focused on viral processes that emerged as key determinants of infection threshold: the efficiency of viral antagonism of innate immune signaling, the viral replication rate, and the rate of formation of compartmentalized replication complexes. Interferon prophylactic efficacy is significantly augmented by all three mechanisms: attenuating viral immune antagonism ($V_{I,N}$), restricting viral replication kinetics ($k_{r,V}$), or promoting dsRNA leakage into the cytosol ($k_{l,V}$). By shifting the viral-host equilibrium toward immune-mediated control, these interventions enhance the potency of IFN. This synergistic effect is particularly pronounced at low to intermediate IFN doses where IFN only partially restrains the infection and viral fitness plays a dominant role in defining disease progression. Within this sensitive regime, IFN potency is significantly augmented by modest attenuation of viral antagonism (e.g., $V_{I,N}$ reduced to ~0.25× baseline), moderate inhibition of replication kinetics ($k_{r,V}$ decreased to ~0.03--0.3× baseline), or accelerated dsRNA leakage ($k_{l,V}$ increased to ~3--30× baseline) substantially increase IFN effectiveness by collectively tipping the viral-host equilibrium toward host-mediated control. To quantify the therapeutic benefit of DAA supplementation, we calculated the fold change in nAOC across varied viral parameters (Figs 5D and S15). These process-specific interventions markedly potentiated IFN-mediated suppression, with the most pronounced synergistic gains observed at low IFN concentrations. Conversely, at higher dosages, IFN monotherapy proved sufficient to achieve robust viral inhibition independently, suggesting a dose-dependent saturation point for the relative benefit conferred by combination therapy.

Together, these findings underscore the crucial role of early IFN intervention in prophylactic settings, particularly when combined with direct-acting antivirals. They further demonstrate how synergy between antiviral therapeutics and innate immune responses can be strategically exploited to enhance antiviral activity while minimizing IFN-associated toxicity [13,14].

## Discussion

By integrating representations of the viral life cycle with innate immune signaling pathways, our approach provides a framework to analyze the coupled dynamics of virus-host interactions. Specifically, the inclusion of the RIG-I-IFN and JAK-STAT signaling pathways, along with dsRNA-induced immune activation and viral immune evasion mechanisms, allows us to examine the feedback loops and molecular interactions that drive these dynamics. Our model suggests infection threshold points that determine infection outcomes, transitioning between “virus-high, ISG-low” and “virus-low, ISG-high” states, governed by thresholds in viral antagonism and immune antiviral activity. These thresholds reveal an inverse relationship between viral load and ISG levels, as observed in single-cell studies. Similar bimodal infection have been observed in other single-cell experiments [32,45,48,69,70]. For example, O’Neal *et al.* [69] demonstrated that in West Nile virus-infected cells, ISG expression declined with increasing viral abundance due to antagonism of the IFN-I signaling. While such “all-or-nothing” ISG induction has been attributed to cell-intrinsic noise previously [32], our model suggests that both viral and host factors shape the infection outcomes. This is consistent with studies on IFN-I dynamics [31,45,48], which link variability in viral replication, IFN-I signaling, and amplification capacity to the all-or-nothing induction of antiviral states.

By incorporating viral dsRNA dynamics and their role in triggering RIG-I signaling, our model demonstrates the balance between viral replication and immune detection and emphasizes the importance of early immune activation. RIG-I-mediated responses, as observed in JEV, are driven by the kinetics of dsRNA compartmentalization and the availability of replication intermediates in the cytoplasm [58,81]. This is in agreement with research showing that interfering with replication compartments or viral mutants that have impaired shielding lead to increased dsRNA leakage, which boosts immune activation and assists in viral clearance [82–84]. This increased dsRNA accessibility serves as a potent immunological trigger that potentiates innate sensing, effectively facilitating viral clearance. Based on our analysis, we propose two strategies employed by (+)RNA viruses to balance replication and immune evasion. *Virulent Replicators*, such as poliovirus and JEV, produce dsRNA rapidly, triggering immune responses but countering this with immune suppression to complete replication before clearance. On the other hand, *Latent Replicators*, such as HCV and dengue virus, replicate more slowly but effectively shield dsRNA within membranous compartments, thereby evading detection and establishing chronic infections. We propose that these strategies reflect a trade-off between replication and immune evasion. Our model provides a framework for differentiating these viral strategies and analyzing how the viral life cycle and immune evasion influence infection outcomes.

We identified three distinct phases in the antiviral immune response: an early sensing and activation phase, an intermediate signaling phase, and a late resolution phase. Early responders like aMAVS, pIKK, and pTBK1 are rapidly induced by viral RNA sensing through RIG-I, consistent with known RLR signaling mechanisms [9]. These activation markers act as proximal hubs facilitating downstream signaling, with their timing being critical for an effective response to RNA viruses. For instance, activated MAVS aggregates form antiviral signalosomes providing a scaffold for sustained signaling that sustains prolonged IRF3/7 and NFκB activation, [85,86]. Likewise, TBK1 and IKKϵ phosphorylation serve as a critical early checkpoint, connecting MAVS activation to IRF3/7 phosphorylation and the induction of interferon genes [87]. In the intermediate phase, IRF and IFN-related factors drive the expression of antiviral ISGs, although this response is suppressed under high viral loads. During the late phase, IFN signaling through JAK-STAT components becomes prominent but is often attenuated in severe infections due to feedback inhibition or impaired upstream signaling [56,81]. These temporally distinct phases help identify key regulators at each stage, providing a framework to prioritize therapeutic targets based on the timing and nature of immune engagement. For instance, we observed that phosphorylated IRF7, nascent IFN transcripts, and early ISGs were consistently different between mild and severe infections early in the response, making them strong predictors of infection outcomes. This is supported by experimental evidence showing that IRF7 is rapidly phosphorylated upon viral sensing, driving the second wave of type I IFN production essential for antiviral defense. IRF7 deficiency severely impairs early IFN responses, underscoring its critical role in immune activation [88–90]. Consistent with our model’s predictions, type I interferons, particularly interferon β (IFN-β), are recognized as markers of effective immune response, where reduced peripheral IFN-β levels during SARS-CoV-2 infection is associated with the severity of the disease [91]. Thus, these temporally resolved immune effector modules elucidate the complexity of immune models and identify mechanistic checkpoints at different phases.

A crucial emergent property of our integrated signaling architecture is its capacity to naturally recapitulate dose-dependent IFN desensitization. Our model systematically identifies the receptor-TYK2 complex (RTKC) as the dominant molecular bottleneck driving this signaling refractoriness. This identification is consistent with extensive experimental evidence demonstrating that TYK2 is essential for type I IFN signaling and the STAT-dependent induction of negative regulators, such as SOCS and USP18 [9,71,75–77,92–94]. Notably, our simulations demonstrate that selectively restoring the RTKC—but not downstream signaling intermediates—reinstates signaling competence, mechanistically validating this receptor-proximal node as the primary determinant of cellular responsiveness. Prolonged or repeated IFN exposure is known to reduce receptor availability and enhance inhibition or destabilization of receptor–kinase complexes by these negative regulators. In agreement with these observations, our model predicts that RTKC depletion is a key determinant of JAK-STAT signaling, providing a mechanistic explanation for IFN desensitization that emerges naturally from the modeled regulatory architecture.

We also demonstrate how low-dose prophylactic administration of IFN can considerably inhibit HCV replication, surpassing the effectiveness of post-infection treatment regimes. This is consistent with previous studies with SARS-CoV-2 and dengue, where delayed IFN treatment was shown to be ineffective and even detrimental in humans and *in vitro* experiments [78–80]. Our findings contribute to a growing body of work suggesting that the timing and dosage of immune interventions can strongly influence disease outcomes [95,96]. Further, we demonstrate how model-informed combination therapies, including those targeting viral processes and inherent antagonistic feedback loops, can significantly enhance IFN prophylaxis. In fact, pairing IFN-α2a with direct-acting antivirals like nucleoside analogues (for example, remdesivir or EIDD-2801) or agents targeting the host (such as camostat or cycloheximide) has demonstrated considerable enhancement of IFN-induced antiviral effects and improved viral inhibition in both *in vitro* and *in vivo* studies [97]. Similar evidence from human trials with intranasal prophylactic administration of IFN-α significantly reduced the incidence and severity of rhinovirus and coronavirus infections, while inconsistent benefits were reported for post-exposure prophylaxis [98]. Thus, interventions employing IFN prophylaxis, especially in high-risk populations or during outbreaks, might be a viable strategy.

By distilling multi-layered host-virus interactions into dominant regulatory motifs, our framework prioritizes computational tractability and analytical clarity while circumventing the parameter redundancy inherent in explicit molecular models. Nevertheless, our framework provides a robust platform for system identification, allowing researchers to translate complex host-virus interactions into actionable insights through the generalized ‘Inverse modelling’ approach. Our aggregated mathematical parameters are quantitatively recoverable from standard virological assays [26]. To parameterize the model for any viral system, experimentalists can establish baseline replication and translation kinetics by generating high-resolution time-course data, quantifying intracellular viral RNA (via strand-specific RT-qPCR) and viral proteins (by quantitative Western blots or immunofluorescence). Further, the model’s lumped parameters can be mapped to measurable signaling bottlenecks within that specific host-virus context. For example, the viral sensing rate ($k_{l,V}$) can be constrained via dsRNA immunofluorescence, while the kinetics of host signaling activation and effector output are quantifiable through IRF3/7 phosphorylation and ISG expression levels. Our framework also predicts a nonlinear Viral-Immune Transition Boundary (VITB) where infection fate is governed by the critical balance between host antiviral potency ($I_{V,N}$) and viral antagonism ($V_{I,N}$). This identifies an experimentally testable hypothesis wherein targeted perturbations (such as the use of viral mutants or signaling knockdowns) will predictably shift outcomes between clearance and escape, manifesting as the bimodal infection states identified by our sensitivity analysis. Further, the predicted synergy between low-dose prophylactic IFN and direct-acting antiviral inhibitors is testable in cell culture using a 2D dose–timing matrix, an experimental layout that maps directly onto the simulations in Fig 5A. Each of these directions, in turn, can be sharpened by our previously developed PARSEC algorithm — a model-based experimental design framework that identifies the most diagnostic measurements and recovers parameters from sparse or noisy kinetic datasets, providing a formal route from model prediction to experimental validation [99].

Phenomenological models of viral kinetics have been invaluable for fitting systemic viral load trajectories and extracting effective rate constants, but their parsimony makes them unable to resolve the intracellular tipping points that emerge within the first 12–24 hours of infection — and unable to ask, mechanistically, which molecular node is responsible. By explicitly mapping 77 molecular nodes, our framework resolves the rapid, sequential activation of the RIG-I/IFN axis and viral interference that simpler models necessarily average out, and identifies high-leverage bottlenecks — such as the RTKC/TYK2 axis — that dictate the fate of infection before systemic symptoms emerge. The complementary limitation should be stated equally plainly: because parameter identifiability scales poorly with model size, a framework of this scale is not the right tool for precise quantitative predictions of clinical viral loads, nor for estimating parameters *de novo* from sparse data — tasks for which smaller, well-identified models remain superior. The value of the present framework lies instead in qualitative and structural predictions: which node is rate-limiting, which perturbations are synergistic, and where the boundary between infection regimes lies as a function of mechanism. These are predictions about the topology of the host-virus response, not about its precise quantitative magnitude, and we view them as complementary to — not competitive with — three decades of productive low-dimensional modelling. Beyond these scope-level limits, the model also reflects a number of tactical assumptions and simplifications adopted to retain tractability while preserving the essential features of host-virus interactions. For example, our model aggregates the sensing by pattern recognition receptors (PRRs) and assumes an averaged cellular activation. Although multiple classes of PRR (TLRs, RLRs, NLRs, CLRs) are involved in shaping innate immune responses, it is well established that viral dsRNA is a major trigger for antiviral responses [49,50]. Consequently, we limit ourselves to dsRNA-sensing pathways to emulate RNA virus immune detection. To simplify the interpretation of innate immune feedback, we also demonstrate system behavior with a uniform level of negative feedback across all signaling nodes and ignore the strength of context-dependent regulators (e.g., SOCS activity on RTKC), which can vary by cell type and stimulus. Nevertheless, the framework remains highly sensitive to molecular-level interactions, where individual processes exert a system-wide influence on the balance between “virus-high, ISG-low” and “virus-low, ISG-high” states. This modularity is increasingly vital as the field’s understanding of canonical innate immune factors evolves. For example, many effectors traditionally deemed strictly antiviral, including TRIM7, STING, and IFITM family proteins, are now recognized to exhibit functional plasticity and context-dependent proviral roles [100,101]. While the molecular mechanisms governing such functional switching remain specific to viral families and physiological conditions, our framework is inherently amenable to refinement as this biology is further elucidated. We also modelled antivirals as reductions in viral parameters, though incorporating drug pharmacodynamics could improve outcome predictions. Integrating the context-dependent behaviours alongside drug pharmacodynamics and spatio-temporal dynamics, could reveal novel emergent properties such as feedback inversion or state switching, positioning our model as a robust foundation for identifying molecular-specific antiviral strategies across diverse cellular and tissue environments.

In summary, we present a generalizable and integrative modeling framework that defines the fundamental regulatory landscape of virus-host dynamics. The non-linear dependence of viral load on parameters like viral antagonism and immune activation (manifesting in the Viral-Immune Transition Boundary) reveals critical points for shifting outcomes toward viral clearance. Ultimately, our approach provides a predictive roadmap for the development of model-informed antiviral strategies while highlighting the importance of early immune activation, ISG dynamics, and IFN signaling in balancing viral persistence and immune control. In the future, integration of high-resolution experimental data can enable this theoretical framework in identifying novel antiviral targets and optimizing synergistic combination therapies that exploit the fundamental kinetic vulnerabilities of the virus-host interface.

## Methods

### Ethics statement

This is a computational study. No animals or humans and/or their tissue samples were used.

### Models integration and extension

To create a detailed, mechanistic model of the virus–innate immune response, we integrated the detailed intracellular viral life cycle model by Chhajer *et al.* [26] with the RIG-I–IFN–ISG signaling model by Burkart *et al.* [43]. To achieve this integration, we introduced additional variables, parameters, and equations to capture extracellular viral dynamics, the leakage and sensing of double-stranded RNA (dsRNA), the regulation of Interferon Regulatory Factor 7 (IRF7), and multilayered feedback mechanisms involving both viral antagonism and host regulatory pathways.

The viral life cycle model was extended to include extracellular dynamics of viral entry and internalization. Viral entry was modeled as a first-order process governed by the rate constant *k*_en,V_, leading to depletion of extracellular virus (*V*_0_). Internalized virus ($V_{I}$) accumulates as a result and can either undergo uncoating at a rate $k_{f,V}$ or be degraded at a rate $\mu_{V_{I}}$. Upon uncoating, viral RNA is released into the cytoplasm and contributes to the cytoplasmic viral RNA pool (*R*_cyt_). To account for this, the original equation describing cytoplasmic viral RNA dynamics was modified to include an additional term reflecting input from uncoated virus. All other terms in this equation, as well as the ODEs describing the dynamics of structural and non-structural viral proteins ($P_{S},P_{NS}$), replication compartment formation ($RC_{form\_rate}$), and replication complexes within compartments ($RC_{CM}$), remain identical to the original framework by Chhajer *et al.* [26].

A critical extension of the viral model was the explicit inclusion of dsRNA leakage from replication compartments. While the original model did not accommodate this, cytosolic dsRNA serves as a key activator of the innate immune response and is central to coupling viral replication with RIG-I sensing. We therefore introduced a leakage term at rate $k_{l,V}$, following an approach similar to Zitzmann *et al.* [35]. Once in the cytoplasm, dsRNA undergoes natural degradation at a rate $\mu_{r,V}$, can be replenished through the deactivation of RIG-I at a rate $b_{RIGI}$, and is cleared through RIG-I–mediated sensing at a rate proportional to the product of RIG-I and dsRNA concentrations ($k_{\text{RIGI}}\cdot \text{RIGI}\cdot {\text{RNA}}_{ds}$). This formulation establishes the mechanistic link between the viral life cycle and the innate immune module.

The innate immune signaling model of Burkart *et al.* [43] was extended by incorporating the dynamics of Interferon Regulatory Factor 7 (IRF7). IRF7 is a transcription factor essential for the induction of type I interferon genes, particularly in plasmacytoid dendritic cells and during late-phase antiviral responses [102]. It is also responsible for its own induction via IFN-dependent transcription [103]. Given its high degree of homology with IRF3, we assumed IRF7 undergoes phosphorylation and nuclear translocation through a mechanism analogous to IRF3. Specifically, cytoplasmic IRF7 is phosphorylated by activated TBK1 and IKKϵ, translocates into the nucleus, and regulates interferon gene expression. Once in the nucleus, phosphorylated IRF7 (pIRF7) can be dephosphorylated or exported back into the cytoplasm. Its synthesis is driven by translation from IFN-induced mRNA, while constitutive degradation occurs with a first-order decay rate.

In addition to coupling viral and immune processes mechanistically, we introduced multilayered feedback to capture the dynamic regulation that occurs at different levels. On the host side, interferon-stimulated genes (ISGs) target distinct stages of the viral life cycle. For example, CH25H and IFITMs inhibit viral entry [9,12], IFIT and TRIM family proteins disrupt RNA and protein synthesis [9,12], and viperin interferes with assembly and egress by disrupting viral envelope formation [9,12]. In contrast, viruses of the *Flaviviridae* family have evolved to counteract host defenses by antagonizing RIG-I signaling and JAK-STAT signaling cascades, thereby suppressing type I IFN production and signaling [54–56]. Finally, the host itself incorporates intrinsic negative regulatory mechanisms to limit excessive immune activation. In particular, molecules such as SOCS proteins and USP18 dampen IFN-induced signaling, ensuring resolution of the antiviral state [15,74,75].

To quantitatively capture these interacting layers of feedback, we introduced three lumped pseudo-variables representing distinct functional modules: $V_{I,N}$ (efficacy of viral antagonism), $I_{V,N}$ (potency of ISG-mediated antiviral activity), and $I_{I,N}$ (efficiency of host negative regulation of immune signaling). These pseudo-variables act by modulating either the intrinsic process rates or the degradation rates of relevant molecular species.

A complete description of the model, along with equations (S1 Text-Model descriptions), detailed variable definitions, initial conditions (S1 Table), parameter values, and sources (S2 and S4 Tables), is provided in the Supplementary Information. Altogether, the integrated model comprises 77 ordinary differential equations and 129 parameters. No parameters were re-estimated in the present work; all values were taken from the source models, where they had been estimated by approximate Bayesian computation (viral life cycle, Chhajer *et al.* [26] and Zitzmann *et al.* [35]), negative log-likelihood minimisation with multi-start initialisation (RIG-I/IFN signalling, Burkart *et al.* [43]; JAK-STAT signalling, Maiwald *et al.* [29] and Kok *et al.* [15]). Direct quantitative measurements for several parameters were included when available (entry/uncoating rates, Zitzmann *et al.* [35]; ISG mRNA kinetics from Maier *et al.* [70]; IRF7 dynamics from Lopacinski *et al.* [38] and Zou *et al.* [104]). Practical identifiability was assessed in those source studies via profile-likelihood analysis and pairwise posterior correlation. A complete listing of parameters with values, units, sources, and the estimation method used in each source is provided in S2 and S4 Tables. The three lumped pseudo-variables - $I_{V,N}$ (ISG-mediated antiviral suppression), $V_{I,N}$ (viral antagonism), and $I_{I,N}$ (auto negative feedback) - were incorporated as systematic tuning parameters varied across their full biological range to explore the model’s behaviour space. The governing equations and parameter values for each module are documented in full in the SI. Key modifications introduced during integration to mechanistically couple the two (viral and immune) subsystems are described below.

#### Viral life cycle and dsRNA detection.

We added the following variables and equations to the viral life cycle model proposed by Chhajer *et al.* [26] to incorporate the extracellular viral dynamics of viral entry and internalization:


$$\begin{array}{c} \frac{dV_{0}}{dt}=-\frac{k_{\text{en, V}}}{1+I_{V,N}\cdot \text{ISGav}}\cdot V_{0} \end{array}$$ (1)

$$\begin{array}{c} \frac{dV_{I}}{dt}=\frac{k_{\text{en, V}}}{1+I_{V,N}\cdot \text{ISGav}}\cdot V_{0}-\frac{k_{f,V}}{1+I_{V,N}\cdot \text{ISGav}}\cdot V_{I}-\mu_{V_{I}}\cdot V_{I} \end{array}$$ (2)

Subsequently, the equation monitoring the levels of cytoplasmic viral RNA is changed:


$$\begin{array}{c} \frac{dR_{\text{cyt}}}{dt}=k_{e,V}\cdot R_{\text{CM}}-\frac{k_{a,V}}{1+I_{V,N}\cdot \text{ISGav}}\cdot P_{S}\cdot R_{\text{cyt}}-\mu_{r,V}\cdot (1+I_{V,N}\cdot \text{ISGav})\cdot R_{\text{cyt}}-{\text{RC}}_{\text{form\_rate}}+\frac{k_{f,V}}{1+I_{V,N}\cdot \text{ISGav}}\cdot V_{I} \end{array}$$ (3)

$$\begin{array}{c} \frac{d{\mathrm{RNA}}_{\mathrm{ds}}}{dt}=k_{\text{l,V}}\cdot {\text{RC}}_{\text{CM}}+b_{\text{RIGI}}\cdot \text{aRIGI}-\mu_{r,V}\cdot (1+I_{V,N}\cdot \text{ISGav})\cdot {\mathrm{RNA}}_{\mathrm{ds}}-k_{\text{RIGI}}\cdot \text{RIGI}\cdot {\mathrm{RNA}}_{\mathrm{ds}} \end{array}$$ (4)

#### Innate immune signaling pathway.

The dynamics of Interferon Regulatory Factor 7 (IRF7) are modelled as shown below:


$$\begin{array}{c} \frac{d\text{IRF7}}{dt}=k_{71}\cdot \text{Vn2c}\cdot \text{pIRF7}-\text{IRF7}\cdot k_{\text{IRF3-IKKe-TBK1}}\cdot (\text{pIKKe}+\text{aTBK1})\cdot \left(\frac{1}{1+V_{I,N}\cdot P_{NS}}\right)+k_{79}\cdot \text{IRF7m}-\mu_{\text{IRF7}}\cdot \text{IRF7} \end{array}$$ (5)

$$\begin{array}{c} \frac{d\text{pIRF7}}{dt}=\text{IRF7}\cdot \text{Vc2n}\cdot k_{\text{IRF3-IKKe-TBK1}}\cdot (\text{pIKKe}+\text{aTBK1})\cdot \left(\frac{1}{1+V_{I,N}\cdot P_{NS}}\right)-k_{71}\cdot \text{pIRF7} \end{array}$$ (6)

#### Incorporation of feedback at multiple levels by both the virus and innate immune system.

Feedback was modeled as a generic modulation of rates:


$$\begin{array}{c} k_{i}^{\prime }=\frac{k_{i}}{1+\rho [A]} \end{array}$$ (7)

and the degradation rates $\mu_{i}$ are changed as


$$\begin{array}{c} \mu_{i}^{\prime }=\mu_{i}(1+\rho [A]) \end{array}$$ (8)

where $k_{i}$ denotes rate constants of viral entry ($k_{en,V}$), fusion ($k_{f,V}$), replication ($k_{r,V}$), translation ($k_{t,V}$), egress ($k_{a,V}$), and $\mu_{i}$ represents degradation rates of viral RNA ($\mu_{r,V}$), and proteins ($\mu_{p,V}$), ρ is $I_{V,N}$ and [*A*] is levels of antiviral ISG (ISGav). For immune signaling processes, $\rho =V_{I,N}$, with viral proteins ($P_{NS}$) attenuating MAVS activation (*k*_MAVS_), IRF3/IRF7 activation ($k_{IRF3-IKKe-TBK1}$), and STAT complex activation (*k*_13_). In addition, host-driven negative feedback is incorporated by setting $\rho =I_{I,N}$ and [*A*]= ISGn, which downregulates activated receptor complex (ARC) formation (*k*_7_).

### Calculation of critical values and infection threshold sensitivity analysis

To identify the critical value of $V_{I,N}^{*}$ (or $I_{V,N}^{*}$), we systematically vary one of $V_{I,N}$ or $I_{V,N}$ keeping the other fixed, and record the extracellular viral concentration at the end of the simulation ($V_{T}$). Next, we evaluate the first-order numerical derivative of the logarithm of viral output ($\log_{10}(V_{T})$) with respect to the variable varied and identify the point of maximum change as the critical value. $V_{I,N}$ and $I_{V,N}$ were varied in the logarithmic scale to span a large dynamic range.

To evaluate how different model parameters influence the critical values of immune activity and viral antagonism, we performed infection threshold Sensitivity Analysis. To evaluate the sensitivity of $V_{I,N}^{*}$ at the infection threshold point, ‘($V_{I,N}^{*,a}$, $I_{V,N}^{*,a}$)’, we fix $I_{V,N}$ as $I_{V,N}^{*,a}$. When we look at the ‘$i^{th}$’ model parameter, we alter the value of the parameter from its nominal value ($\theta_{i}^{0}$) to $\theta_{i}$ and re-evaluate the critical value of viral antagonism ($V_{I,N}^{*}(\theta_{i})$). Finally, we evaluate the sensitivity index as the ratio of the relative change in threshold value with respect to the relative change in the parameter value, using the following equation:


$$\begin{array}{c} SI_{i}(V_{I,N})=\left(\frac{\Delta_{i}V_{I,N}^{*}}{V_{I,N}^{*,a}}\right)/\left(\frac{\Delta \theta_{i}}{\theta_{i}}\right) \end{array}$$ (9)

As sensitivity captures the slope of the threshold function with respect to the model parameter, we use a five-point central difference formula instead of the naive single-point difference formula, to calculate the sensitivity index more accurately. The threshold values were evaluated at $\theta_{i}=[0.9,0.95,1,1.05,1.1]\times \theta_{i}^{0}$.


$$\begin{array}{c} \Delta_{i}V_{I,N}^{*}=\frac{V_{I,N}^{*}(0.9\times \theta_{i}^{0})-8\cdot V_{I,N}^{*}(0.95\times \theta_{i}^{0})+8\cdot V_{I,N}^{*}(1.05\times \theta_{i}^{0})-V_{I,N}^{*}(1.1\times \theta_{i}^{0})}{12} \end{array}$$ (10)

$SI_{i}(V_{I,N})$ determines the influence of the ‘$i^{th}$’ model parameter on the critical viral antagonism required for immune escape. Analogously, we can calculate $SI_{i}(I_{V,N})$; here we fix $V_{I,N}$ as $V_{I,N}^{*,a}$, and assess how changes in the model parameters affect the immune activity threshold ($I_{V,N}^{*}$) necessary for viral suppression. To identify the most important parameters that influence both the thresholds, we rank the parameters based on threshold sensitivity score, calculated as the sum of squares of the corresponding sensitivity indices, i.e., $(SI_{i}(V_{I,N}){)}^{2}+(SI_{i}(I_{V,N}){)}^{2}$

### Latin Hypercube Sampling and correlation with viral load

To evaluate the influence of host and viral parameters on infection outcomes, we performed Latin Hypercube Sampling (LHS). Each model parameter $p_{i}$ was varied within a log-uniform range


$$\begin{array}{c} p_{i}\in \left[0.1\cdot p_{i}^{\left(0\right)},10\cdot p_{i}^{\left(0\right)}\right], \end{array}$$ (11)

where $p_{i}^{\left(0\right)}$ denotes the original value of parameter *i*. A total of *N* = 25000 parameter sets were generated, each comprising the full parameter vector,


$$\begin{array}{c} {𝐩}^{\left(j\right)}=\{p_{1}^{\left(j\right)},p_{2}^{\left(j\right)},\ldots ,p_{M}^{\left(j\right)}\},\;j=1,\ldots ,N, \end{array}$$ (12)

To quantify the relationship between individual parameters and infection outcome, we computed the Pearson correlation coefficient between each parameter $p_{i}$ and the viral load across all LHS-generated parameter sets:


$$\begin{array}{c} C_{i}=\frac{\text{Cov}\left(p_{i},V_{T}\right)}{\sigma_{p_{i}}\cdot \sigma_{V_{T}}},\;i=1,\ldots ,M, \end{array}$$ (13)

where $\sigma_{p_{i}}$ and $\sigma_{V,T}$ denote the standard deviations of parameter $p_{i}$ and viral load $V_{T}$, respectively. The resulting correlation coefficients $C_{i}$ were then ranked to identify the top 10 positively correlated and top 10 negatively correlated parameters. These parameters were visualized in a bar plot to highlight the most influential drivers of viral load variability (Fig 2C).

### Normalized dynamics based clustering

In our analysis, we perform hierarchical clustering on normalized time-series data for each model variable under three conditions - A: low $V_{T}$ (when $V_{I,N}$ = 0.5$V_{I,N}^{*}$), B: critical $V_{T}$ (when $V_{I,N}$ = $V_{I,N}^{*}$), and C: high $V_{T}$ (when $V_{I,N}$ = 2$V_{I,N}^{*}$). Variables with zero standard deviation or with a low coefficient of variation ($\frac{\sigma_{i}}{\mu_{i}}<0.05$) were excluded, ensuring retention of only dynamically informative variables. We then concatenate the dynamics of the selected variables in the three regimes and normalize them using Z-score normalization. The mean and standard deviation used for the normalization is based on the concatenated dynamics.

A pairwise distance matrix was computed across all retained variables, followed by hierarchical clustering based on the average linkage criterion. The resulting dendrogram was cut to yield k = 8 clusters. For each cluster, variable memberships and their average correlation to the cluster mean trajectory were computed. Variable-wise dynamics were additionally annotated with marker sizes inversely proportional to the distance to the centroid.

This analysis was based on our recently published model-based experiment design algorithm, PARSEC [99]. Like the analysis here, the PARSEC groups model variables/observables, but the clustering is based on differences in parameter sensitivity signatures instead of dynamical correlations.

**Quantification of dynamic differences using a $\chi^{2}$-like trajectory score**

To quantify dynamic differences in temporal responses between conditions, we defined a log-transformed mean squared deviation ($\chi^{2}$-like) score for each variable. For a given variable $x_{k}$, the score between two conditions *c*_1_ and *c*_2_ was calculated as:


$$\begin{array}{c} {\chi^{2}}_{k}^{\left(c_{1},c_{2}\right)}=\frac{1}{T}\sum_{t=1}^{T}{\left[\log_{10}\left(x_{k}^{\left(c_{1}\right)}(t)+\epsilon \right)-\log_{10}\left(x_{k}^{\left(c_{2}\right)}(t)+\epsilon \right)\right]}^{2}, \end{array}$$ (14)

where $x_{k}^{\left(c\right)}(t)$ denotes the trajectory of variable *k* at time *t* under condition *c*, *T* is the number of time poin*t*s. This measure captures the average squared deviation between log-scaled trajectories and is mathematically equivalent to a $\chi^{2}$-like score. A small positive number ϵ = 10^−8^ is added to the variables so that the resulting logarithmic value is defined and finite.

We applied this metric in two distinct contexts. First, in the clustering analysis, it was used to compare responses between conditions A and C (Fig 3C). Second, in the interferon (IFN) stimulation experiments, the same metric was employed to compare trajectories between two IFN priming protocols: one in which cells received 100 nM IFN at 24 h following a 1 nM priming dose, and another in which the same 100 nM boost followed a 100 nM priming dose (Fig 4C).

### Normalized area over the curve (nAOC) calculation - estimating prophylactic efficiency of IFN pretreatment

To evaluate the prophylactic action, we simulate the effect of various dosages of IFN (as shown in Fig 5) administered *k* days before the start of infection, where k∈[0,1,2,…13]. We report the resulting viral load at 96 hours post-infection ($V_{T}(k)$). In the logarithmic scale, we compute the area between this curve ($\log_{10}(V_{T}(t))$) and a horizontal line at $y_{th}=-0.75$ (a high viral load threshold reference) between *k* = 0 to *k* = 13 days using the trapezoidal rule. Specifically, the AOC was calculated using:


$$\begin{array}{c} AOC=\Sigma_{k=0}^{13}(y_{th}-\log_{10}(V_{T}(k)) \end{array}$$ (15)

## Supporting information

S1 Text Model Description. (PDF)

S2 Text Evaluation of model predictions against Aunins *et al.* (2018) data. (PDF)

S1 Table Description of model variables and their initial condition values used in simulation. (PDF)

S2 Table HCV-specific viral life-cycle parameters. The estimation method listed reflects the approach employed in the cited reference.
(PDF)

S3 Table JEV-specific viral life-cycle parameters. The estimation method listed reflects the approach employed in the cited reference.
(PDF)

S4 Table Description of model parameters and their values. The estimation method listed reflects the approach employed in the cited reference.
(PDF)

S1 FigComparison of model predictions with intracellular HCV RNA dynamics.The total intracellular (+)RNA predicted by the model ($R_{cyt}+R_{CM}+RC_{CM}$, blue line) is compared against experimentally measured HCV RNA abundances from Aunins *et al.* (2018) (red circles). Initial conditions were set to match the values reported in Aunins *et al.* (2018); no parameter fitting was performed. The model reproduces the characteristic biphasic rise and plateau of intracellular viral RNA over 48 h post-infection.(TIF)

S2 FigEffect of multiplicities of infection (MOIs) on the dynamics.Normalized viral titre trajectories are shown for different MOIs under the four scenarios: $S_{0},(I_{n}=0,\;I_{a}=0,\;VC=0);S_{1a},(I_{n}=0,\;I_{a}=1,\;VC=0);S_{1b},(I_{n}=1,\;I_{a}=1,\;VC=0);\text{and }S_{IV},(I_{n}=1,\;I_{a}=1,\;VC=1)$.(TIF)

S3 FigIntegrated mathematical model of the viral life cycle with interferon pathways.Normalised dynamics (for JEV) of the levels of viral titre (left), non-structural proteins (center), and antiviral ISGs (right) are shown and compared for different combinations of strengths of immune self-regulation ($I_{I,N}),\text{ viral counter measures}(V_{I,N})\text{ and antiviral immune response }(I_{V,N}$). The blue line represents a no-interaction ($S_{0})\text{ scenario where }I_{V,N},\;I_{I,N},\;V_{I,N}\text{ are all set to zero},\text{ whereas the red solid line represents a fully}$$\text{interacting system where all three are set to one }(S_{IV}).\text{ The black dashed and magenta lines correspond}$$\text{to intermediate interactions }(S_{Ib})\text{ where }‘I_{V,N}=I_{I,N}=1\text{ and }V_{I,N}=0$’ and scenario ($S_{Ia})‘I_{V,N}=1I_{I,N}=V_{I,N}=0$’ respectively.(TIF)

S4 FigViral-Immune transition of HCV infection dynamics.(a-b) The heatmap of the viral titre output ($V_{T})\;\text{during}\;\text{HCV}\;\text{infection}\;\text{is}\;\text{shown}\;\text{when}\;k_{t,V}\;\text{and}\;k_{t,ISG\;RNA}\;(\text{left})\;\text{and}\;k_{l,V}\;\text{and}\;k_{r,V}$ (right). Darker shades indicate higher viral load, highlighting the nonlinear response of viral output to changes in host-virus interaction parameters. c) Viral titre ($V_{T})\text{as a function of viral transcription rate }k_{t,V},\text{ showing a sharp transition between low and high viral states}.$ d) Viraltitre (*V*_T_) as a function of viral antagonism strength *V_I,N_*, demonstrating a sigmoidal response characteristic of a threshold-like antiviral effect.(TIF)

S5 FigVirus-immune transition boundary in JEV infection.The heatmap of the viral titre output ($V_{T})\text{ during JEV infection is shown as a function of }V_{I,N}\text{ and immune action strength }(I_{V,N}$). Darker shades indicate higher viral load, highlighting the nonlinear response of viral output to changes in host-virus interaction parameters.(TIF)

S6 FigHCV viral load at the immune–virus critical point under parameter variability.Distribution of Hepatitis C viral load predicted from 25,000 parameter sets generated by Latin Hypercube Sampling (LHS), with all model parameters varied independently over a 100-fold range, from 0.1× to 10× their nominal values. Here we fix $V_{I,N}=0.578\text{ and }I_{V,N}$ = 1 (critical point).(TIF)

S7 FigSensitivity analysis identifies key parameters controlling viral-immune transition boundary.Bar plot showing the parameter sum of sensitivity indices of critical values (corresponding to VITB) of $I_{V,N}^{*}\;\text{and}\;V_{I,N}^{*}.\;\text{Colored}\;\text{bars}\;\text{denote}\;\text{the}\;\text{most}\;\text{influential}\;\text{parameters}:b_{MAVS},b_{KINASE},k_{t,V},k_{MAVS},k_{l,V},k_{r,V},\mu_{ISGRNA},\;\text{and}\;k_{t,ISGRNA}$ (Description in Supplementary S2 and S4 Tables).(TIF)

S8 FigModulation of JEV VITB by ISG translation and leakage rate of viral dsRNA.The Viral-Immune Transition boundary (VITB) for JEV infection systems are shown for different values of $k_{t,ISG\;RNA}\;(\text{translation}\;\text{rate}\;\text{of}\;\text{ISG}\;\text{mRNA},\;\text{left})\;\text{and}\;k_{l,V}$ (leakage rate of viral dsRNA, right). The solid line, the dashed line, and the solid line with square symbols show the VITB when the parameter of interest is unperturbed, reduced by half, and doubled, respectively.(TIF)

S9 FigComparative VITB analysis of HCV and JEV.The VITB fronts for HCV (blue) and JEV (orange) infection systems are shown for different values of $\mu_{ISGRNA}\;(\text{degradation of ISG mRNA},\text{ top left}),\;k_{t,V}(\text{translation rate of viral RNA},\text{ top right}),$$k_{r,V}\;(\text{transcription rate of viral RNA},\text{top center}),\;b_{MAVS}\;(\text{deactivation rate of MAVS},\text{ bottom left}),\;k_{MAVS}\;(\text{activation}$$\text{rate of MAVS},\text{ bottom right}),\text{ and }b_{KINASE}$ (deactivation rate of kinases, bottom center). The solid line, the dashed line, and the solid line with square symbols show the VITB when the parameter of interest is unperturbed, reduced by half, and doubled, respectively.(TIF)

S10 FigViral load sensitivity along the JEV VITB.The line plots show the fold change in the steady state values of JEV $V_{T}$ at points along the front when the corresponding parameter values were **A** reduced by 50% (magenta-$k_{t,ISG\;RNA}$, green-$k_{l,V}$, black-$k_{MAVS}$) and **B** increased by 50% (black-$\mu_{ISG\;RNA}$, orange-$k_{r,V}$), blue-$k_{t,V}$, grey-$b_{MAVS}$, and violet-$b_{KINASE}$).(TIF)

S11 Fig Coordinated dynamics of the IFN–ISG transcriptional regulatory hub. The temporal profiles of components of each cluster IFN-ISG transcriptional hub cluster across the 96-hour time course are plotted. The size of the filled grey circle indicates the correlation between the component and the cluster mean (Fig 3B), with larger markers representing smaller distances.
(TIF)

S12 FigDistinct dynamic phases of early and secondary innate immune activation.The temporal profiles of components of each cluster **A** IRF3-IFN cluster, **B** secondary responders cluster, **C** NFκB−IκBac, **D** aRIGI, and **E** pIRF7 across the 96-hour time course are plotted. The size of the filled grey circle indicates the correlation between the component and the cluster mean (Fig 3B), with larger markers representing smaller distances. As clusters **C**, **D**, and **E** contain only one component, correlation is not applicable.(TIF)

S13 FigTemporal clustering reveals coordinated modules in innate immune signaling dynamics.The temporal profiles of components of each cluster **A** Early responders cluster, and **B** $NF\kappa B_{c}$ cluster across the 96-hour time course are plotted. The size of the filled grey circle indicates the correlation between the component and the cluster mean (Fig 3B), with larger markers representing smaller distances. As clusters **B** contain only one component, correlation is not applicable.(TIF)

S14 Fig IFN Desensitization is Mediated by Receptor-Kinase Complex and TYK Dynamics. Kinetics of **A** an early ISG (ISGn) transcript involved in immune suppression, and **B** a late ISG (ISGav) transcript associated with viral suppression, in response to a two-dose interferon (IFN) treatment. The first IFN dose (100 nM) is administered at 0 h post-exposure (h.p.e.), followed by a second 100 nM dose at 24 h.p.e. The following five conditions are shown: D_2_ Control (orange circles): Two-dose IFN treatment without any restoration of signaling components. D_2_ JAK Restored (yellow triangles): JAK levels restored to baseline before the second IFN dose. D_2_ RTKC Restored (purple diamonds): Receptor–TYK2 complex (RTKC) levels restored before the second IFN dose. D_2_ TYK Restored (green line): TYK2 levels restored before the second IFN dose. D_2_ RTKC and TYK Restored (light blue line): Both RTKC and TYK2 levels restored before the second IFN dose. Dashed vertical lines indicate the timing of the second IFN dose. These results suggest that restoration of upstream signaling components (particularly RTKC) can partially recover ISG responsiveness upon repeated IFN stimulation.
(TIF)

S15 FigEffect of IFN dosage and timing on viral infection.Heatmaps illustrating the fold change in normalized AOC (nAOC) as a function of IFN dosage (nM) and viral parameters: **A** viral egress rate ($k_{a,V}$), **B** viral translation rate ($k_{t,V}$), **C** viral RNA degradation rate ($\mu_{r,V}$), and **D** viral entry rate ($k_{en,V}$). Warmer colors (pink) indicate higher fold changes in nAOC, signifying improved prophylactic efficacy.(TIF)

## References

[1] Chung Y-S, Lam C-Y, Tan P-H, Tsang H-F, Wong S-CC. Comprehensive Review of COVID-19: Epidemiology, Pathogenesis, Advancement in Diagnostic and Detection Techniques, and Post-Pandemic Treatment Strategies. Int J Mol Sci. 2024;25(15):8155. doi: 10.3390/ijms25158155 39125722 PMC11312261

[2] Haider N, Hasan MN, Onyango J, Asaduzzaman M. Global landmark: 2023 marks the worst year for dengue cases with millions infected and thousands of deaths reported. IJID Reg. 2024;13:100459. doi: 10.1016/j.ijregi.2024.100459 39497753 PMC11532885

[3] Sallam M, Khalil R. Contemporary Insights into Hepatitis C Virus: A Comprehensive Review. Microorganisms. 2024;12(6):1035. doi: 10.3390/microorganisms12061035 38930417 PMC11205832

[4] Romero-Brey I, Bartenschlager R. Membranous replication factories induced by plus-strand RNA viruses. Viruses. 2014;6(7):2826–57. doi: 10.3390/v6072826 25054883 PMC4113795

[5] Miller S, Krijnse-Locker J. Modification of intracellular membrane structures for virus replication. Nat Rev Microbiol. 2008;6(5):363–74. doi: 10.1038/nrmicro1890 18414501 PMC7096853

[6] Apte-Sengupta S, Sirohi D, Kuhn RJ. Coupling of replication and assembly in flaviviruses. Curr Opin Virol. 2014;9:134–42. doi: 10.1016/j.coviro.2014.09.020 25462445 PMC4268268

[7] Paul D, Bartenschlager R. Flaviviridae Replication Organelles: Oh, What a Tangled Web We Weave. Annu Rev Virol. 2015;2(1):289–310. doi: 10.1146/annurev-virology-100114-055007 26958917

[8] Takeuchi O, Akira S. Pattern recognition receptors and inflammation. Cell. 2010;140(6):805–20. doi: 10.1016/j.cell.2010.01.022 20303872

[9] Schneider WM, Chevillotte MD, Rice CM. Interferon-stimulated genes: a complex web of host defenses. Annu Rev Immunol. 2014;32:513–45. doi: 10.1146/annurev-immunol-032713-120231 24555472 PMC4313732

[10] Yoneyama M, Kikuchi M, Natsukawa T, Shinobu N, Imaizumi T, Miyagishi M, et al. The RNA helicase RIG-I has an essential function in double-stranded RNA-induced innate antiviral responses. Nat Immunol. 2004;5(7):730–7. doi: 10.1038/ni1087 15208624

[11] Barral PM, Sarkar D, Su Z, Barber GN, DeSalle R, Racaniello VR, et al. Functions of the cytoplasmic RNA sensors RIG-I and MDA-5: key regulators of innate immunity. Pharmacol Ther. 2009;124(2):219–34. doi: 10.1016/j.pharmthera.2009.06.012 19615405 PMC3165056

[12] Schoggins JW, Rice CM. Interferon-stimulated genes and their antiviral effector functions. Curr Opin Virol. 2011;1(6):519–25. doi: 10.1016/j.coviro.2011.10.008 22328912 PMC3274382

[13] Kirkwood JM, Bender C, Agarwala S, Tarhini A, Shipe-Spotloe J, Smelko B, et al. Mechanisms and management of toxicities associated with high-dose interferon alfa-2b therapy. J Clin Oncol. 2002;20(17):3703–18. doi: 10.1200/JCO.2002.03.052 12202672

[14] Sleijfer S, Bannink M, Van Gool AR, Kruit WHJ, Stoter G. Side effects of interferon-alpha therapy. Pharm World Sci. 2005;27(6):423–31. doi: 10.1007/s11096-005-1319-7 16341948

[15] Kok F, Rosenblatt M, Teusel M, Nizharadze T, Gonçalves Magalhães V, Dächert C, et al. Disentangling molecular mechanisms regulating sensitization of interferon alpha signal transduction. Mol Syst Biol. 2020;16(7):e8955. doi: 10.15252/msb.20198955 32696599 PMC7373899

[16] Chan YK, Gack MU. Viral evasion of intracellular dna and rna sensing. Nat Rev Microbiol. 2016;14(6):360–73. 27174148 10.1038/nrmicro.2016.45 PMC5072394

[17] Beachboard DC, Horner SM. Innate immune evasion strategies of DNA and RNA viruses. Curr Opin Microbiol. 2016;32:113–9. doi: 10.1016/j.mib.2016.05.015 27288760 PMC4983539

[18] Chen Y, Shi Y, Wu J, Qi N. MAVS: A Two-Sided CARD Mediating Antiviral Innate Immune Signaling and Regulating Immune Homeostasis. Front Microbiol. 2021;12:744348. doi: 10.3389/fmicb.2021.744348 34566944 PMC8458965

[19] Padmanabhan P, Garaigorta U, Dixit NM. Emergent properties of the interferon-signalling network may underlie the success of hepatitis C treatment. Nat Commun. 2014;5:3872. doi: 10.1038/ncomms4872 24834957

[20] Owens K, Esmaeili S, Schiffer JT. Heterogeneous SARS-CoV-2 kinetics due to variable timing and intensity of immune responses. JCI Insight. 2024;9(9):e176286. doi: 10.1172/jci.insight.176286 38573774 PMC11141931

[21] Van der Hoek L. Human coronaviruses: what do they cause? Antivir Ther. 2007;12(4_part_2):651–8. 17944272

[22] Tay DJW, Lew ZZR, Chu JJH, Tan KS. Uncovering novel viral innate immune evasion strategies: what has sars-cov-2 taught us? Front Microbiol. 2022;13:844447. 35401477 10.3389/fmicb.2022.844447 PMC8984613

[23] Binder M, Sulaimanov N, Clausznitzer D, Schulze M, Hüber CM, Lenz SM, et al. Replication vesicles are load- and choke-points in the hepatitis C virus lifecycle. PLoS Pathog. 2013;9(8):e1003561. doi: 10.1371/journal.ppat.1003561 23990783 PMC3749965

[24] Dahari H, Ribeiro RM, Rice CM, Perelson AS. Mathematical modeling of subgenomic hepatitis C virus replication in Huh-7 cells. J Virol. 2007;81(2):750–60. 17035310 10.1128/JVI.01304-06 PMC1797446

[25] Aunins TR, Marsh KA, Subramanya G, Uprichard SL, Perelson AS, Chatterjee A. Intracellular hepatitis C virus modeling predicts infection dynamics and viral protein mechanisms. J Virol. 2018;92(11). 10.1128/JVI.02098-17 PMC5952170 29563295

[26] Chhajer H, Rizvi VA, Roy R. Life cycle process dependencies of positive-sense RNA viruses suggest strategies for inhibiting productive cellular infection. J R Soc Interface. 2021;18(184):20210401. doi: 10.1098/rsif.2021.0401 34753308 PMC8580453

[27] Larkin CI, Dunn MD, Shoemaker JE, Klimstra WB, Faeder JR. A detailed kinetic model of Eastern equine encephalitis virus replication in a susceptible host cell. PLoS Comput Biol. 2025;21(6):e1013082. doi: 10.1371/journal.pcbi.1013082 40465541 PMC12136344

[28] Smieja J, Jamaluddin M, Brasier AR, Kimmel M. Model-based analysis of interferon-beta induced signaling pathway. Bioinformatics. 2008;24(20):2363–9. doi: 10.1093/bioinformatics/btn400 18713791 PMC2720726

[29] Maiwald T, Schneider A, Busch H, Sahle S, Gretz N, Weiss TS, et al. Combining theoretical analysis and experimental data generation reveals IRF9 as a crucial factor for accelerating interferon α-induced early antiviral signalling. FEBS J. 2010;277(22):4741–54. doi: 10.1111/j.1742-4658.2010.07880.x 20964804

[30] Kalliara E, Kardynska M, Bagnall J, Spiller DG, Müller W, Ruckerl D, et al. Post-transcriptional regulatory feedback encodes jak-stat signal memory of interferon stimulation. Front Immunol. 2022;13:947213. 36238296 10.3389/fimmu.2022.947213 PMC9552616

[31] Tan J, Pan R, Qiao L, Zou X, Pan Z. Modeling and dynamical analysis of virus-triggered innate immune signaling pathways. PLoS One. 2012;7(10):e48114. doi: 10.1371/journal.pone.0048114 23118935 PMC3484162

[32] Rand U, Rinas M, Schwerk J, Nöhren G, Linnes M, Kröger A, et al. Multi-layered stochasticity and paracrine signal propagation shape the type-I interferon response. Mol Syst Biol. 2012;8:584. doi: 10.1038/msb.2012.17 22617958 PMC3377992

[33] Zhang W, Zou X. Systematic analysis of the mechanisms of virus-triggered type I IFN signaling pathways through mathematical modeling. IEEE/ACM Trans Comput Biol Bioinform. 2013;10(3):771–9. doi: 10.1109/TCBB.2013.31 24091409

[34] Zhang W, Tian T, Zou X. Negative feedback contributes to the stochastic expression of the interferon-β gene in virus-triggered type I interferon signaling pathways. Math Biosci. 2015;265:12–27. doi: 10.1016/j.mbs.2015.04.003 25892253

[35] Zitzmann C, Schmid B, Ruggieri A, Perelson AS, Binder M, Bartenschlager R, et al. A Coupled Mathematical Model of the Intracellular Replication of Dengue Virus and the Host Cell Immune Response to Infection. Front Microbiol. 2020;11:725. doi: 10.3389/fmicb.2020.00725 32411105 PMC7200986

[36] Huang Y, Dai H, Ke R. Principles of Effective and Robust Innate Immune Response to Viral Infections: A Multiplex Network Analysis. Front Immunol. 2019;10:1736. doi: 10.3389/fimmu.2019.01736 31396233 PMC6667926

[37] Castaño-Arcila M, Aguilera LU, Rodríguez-González J. Modeling the intracellular dynamics of the dengue viral infection and the innate immune response. J Theor Biol. 2021;509:110529. doi: 10.1016/j.jtbi.2020.110529 33129952

[38] Lopacinski AAB, Sweatt AJ, Smolko CM, Gray-Gaillard E, Borgman CA, Shah M, et al. Modeling the complete kinetics of coxsackievirus b3 reveals human determinants of host-cell feedback. Cell Systems. 2021;12(4):304–23. 33740397 10.1016/j.cels.2021.02.004 PMC8112228

[39] Katze MG, Fornek JL, Palermo RE, Walters K-A, Korth MJ. Innate immune modulation by RNA viruses: emerging insights from functional genomics. Nat Rev Immunol. 2008;8(8):644–54. doi: 10.1038/nri2377 18654572 PMC7097543

[40] Hubel P, Urban C, Bergant V, Schneider WM, Knauer B, Stukalov A, et al. A protein-interaction network of interferon-stimulated genes extends the innate immune system landscape. Nat Immunol. 2019;20(4):493–502. doi: 10.1038/s41590-019-0323-3 30833792

[41] Elzawahry A, Patil A, Kumagai Y, Suzuki Y, Nakai K. Innate immunity interactome dynamics. Gene Regul Syst Bio. 2014;8:1–15. doi: 10.4137/GRSB.S12850 24453478 PMC3885269

[42] Rajsbaum R, García-Sastre A. Viral evasion mechanisms of early antiviral responses involving regulation of ubiquitin pathways. Trends Microbiol. 2013;21(8):421–9. doi: 10.1016/j.tim.2013.06.006 23850008 PMC3740364

[43] Burkart SS, Schweinoch D, Frankish J, Sparn C, Wüst S, Urban C, et al. High-resolution kinetic characterization of the RIG-I-signaling pathway and the antiviral response. Life Sci Alliance. 2023;6(10):e202302059. doi: 10.26508/lsa.202302059 37558422 PMC10412806

[44] Schulte MB, Andino R. Single-cell analysis uncovers extensive biological noise in poliovirus replication. J Virol. 2014;88(11):6205–12. doi: 10.1128/JVI.03539-13 24648454 PMC4093869

[45] Van Eyndhoven LC, Singh A, Tel J. Decoding the dynamics of multilayered stochastic antiviral IFN-I responses. Trends Immunol. 2021;42(9):824–39. doi: 10.1016/j.it.2021.07.004 34364820

[46] Doğanay S, Lee MY, Baum A, Peh J, Hwang S-Y, Yoo J-Y, et al. Single-cell analysis of early antiviral gene expression reveals a determinant of stochastic IFNB1 expression. Integr Biol (Camb). 2017;9(11):857–67. doi: 10.1039/c7ib00146k 29098213 PMC6201300

[47] Zanini F, Pu S-Y, Bekerman E, Einav S, Quake SR. Single-cell transcriptional dynamics of flavivirus infection. Elife. 2018;7:e32942. doi: 10.7554/eLife.32942 29451494 PMC5826272

[48] Zhao M, Zhang J, Phatnani H, Scheu S, Maniatis T. Stochastic expression of the interferon-β gene. PLoS Biol. 2012;10(1):e1001249. doi: 10.1371/journal.pbio.1001249 22291574 PMC3265471

[49] Rehwinkel J, Gack MU. RIG-I-like receptors: their regulation and roles in RNA sensing. Nat Rev Immunol. 2020;20(9):537–51. doi: 10.1038/s41577-020-0288-3 32203325 PMC7094958

[50] Kawai T, Akira S. The role of pattern-recognition receptors in innate immunity: update on Toll-like receptors. Nat Immunol. 2010;11(5):373–84. doi: 10.1038/ni.1863 20404851

[51] Chen YG, Hur S. Cellular origins of dsRNA, their recognition and consequences. Nat Rev Mol Cell Biol. 2022;23(4):286–301. doi: 10.1038/s41580-021-00430-1 34815573 PMC8969093

[52] Chazal M, Beauclair G, Gracias S, Najburg V, Simon-Lorière E, Tangy F, et al. RIG-I Recognizes the 5’ Region of Dengue and Zika Virus Genomes. Cell Rep. 2018;24(2):320–8. doi: 10.1016/j.celrep.2018.06.047 29996094

[53] Zitzmann C, Dächert C, Schmid B, van der Schaar H, van Hemert M, Perelson AS, et al. Mathematical modeling of plus-strand RNA virus replication to identify broad-spectrum antiviral treatment strategies. PLoS Comput Biol. 2023;19(4):e1010423. doi: 10.1371/journal.pcbi.1010423 37014904 PMC10104377

[54] Li X-D, Sun L, Seth RB, Pineda G, Chen ZJ. Hepatitis C virus protease NS3/4A cleaves mitochondrial antiviral signaling protein off the mitochondria to evade innate immunity. Proc Natl Acad Sci U S A. 2005;102(49):17717–22. doi: 10.1073/pnas.0508531102 16301520 PMC1308909

[55] Seong RK, Lee JK, Shin OS. Zika virus-induction of the suppressor of cytokine signaling 1/3 contributes to the modulation of viral replication. Pathogens. 2020;9(3):163. 32120897 10.3390/pathogens9030163 PMC7157194

[56] Lin R-J, Liao C-L, Lin E, Lin Y-L. Blocking of the alpha interferon-induced Jak-Stat signaling pathway by Japanese encephalitis virus infection. J Virol. 2004;78(17):9285–94. doi: 10.1128/JVI.78.17.9285-9294.2004 15308723 PMC506928

[57] Uchil PD, Satchidanandam V. Characterization of RNA synthesis, replication mechanism, and in vitro RNA-dependent RNA polymerase activity of Japanese encephalitis virus. Virology. 2003;307(2):358–71. doi: 10.1016/s0042-6822(02)00130-7 12667804

[58] Uchida L, Espada-Murao LA, Takamatsu Y, Okamoto K, Hayasaka D, Yu F, et al. The dengue virus conceals double-stranded RNA in the intracellular membrane to escape from an interferon response. Sci Rep. 2014;4:7395. doi: 10.1038/srep07395 25491663 PMC4261170

[59] Lindqvist R, Mundt F, Gilthorpe JD, Wölfel S, Gekara NO, Kröger A, et al. Fast type i interferon response protects astrocytes from flavivirus infection and virus-induced cytopathic effects. J Neuroinflammation. 2016;13(1):277. 27776548 10.1186/s12974-016-0748-7 PMC5078952

[60] Dalrymple NA, Mackow ER. Endothelial cells elicit immune-enhancing responses to dengue virus infection. J Virol. 2012;86(12):6408–15. doi: 10.1128/JVI.00213-12 22496214 PMC3393559

[61] Randall RE, Goodbourn S. Interferons and viruses: an interplay between induction, signalling, antiviral responses and virus countermeasures. J Gen Virol. 2008;89(Pt 1):1–47. doi: 10.1099/vir.0.83391-0 18089727

[62] Katze MG, He Y, Gale MJ. Viruses and interferon: a fight for supremacy. Nat Rev Immunol. 2002;2(9):675–87. 12209136 10.1038/nri888

[63] Fredericksen BL, Gale M Jr. West Nile virus evades activation of interferon regulatory factor 3 through RIG-I-dependent and -independent pathways without antagonizing host defense signaling. J Virol. 2006;80(6):2913–23. doi: 10.1128/JVI.80.6.2913-2923.2006 16501100 PMC1395472

[64] Garcia MN, Hasbun R, Murray KO. Persistence of West Nile virus. Microbes Infect. 2015;17(2):163–8. doi: 10.1016/j.micinf.2014.12.003 25499188

[65] Schilling M, Bridgeman A, Gray N, Hertzog J, Hublitz P, Kohl A, et al. RIG-I Plays a Dominant Role in the Induction of Transcriptional Changes in Zika Virus-Infected Cells, which Protect from Virus-Induced Cell Death. Cells. 2020;9(6):1476. doi: 10.3390/cells9061476 32560274 PMC7349056

[66] Pothlichet J, Burtey A, Kubarenko AV, Caignard G, Solhonne B, Tangy F, et al. Study of human RIG-I polymorphisms identifies two variants with an opposite impact on the antiviral immune response. PLoS One. 2009;4(10):e7582. doi: 10.1371/journal.pone.0007582 19859543 PMC2762520

[67] Lindqvist R, Kurhade C, Gilthorpe J o n a t h a n D, Överby A n n a K. Cell-type-and region-specific restriction of neurotropic flavivirus infection by viperin. J Neuroinflammation. 2018;15(1):80. 29544502 10.1186/s12974-018-1119-3 PMC5856362

[68] Li A, Wang W, Wang Y, Chen K, Xiao F, Hu D, et al. NS5 Conservative Site Is Required for Zika Virus to Restrict the RIG-I Signaling. Front Immunol. 2020;11:51. doi: 10.3389/fimmu.2020.00051 32117232 PMC7033454

[69] O’Neal JT, Upadhyay AA, Wolabaugh A, Patel NB, Bosinger SE, Suthar MS. West nile virus-inclusive single-cell rna sequencing reveals heterogeneity in the type i interferon response within single cells. J Virol. 2019;93(6):10–1128. 10.1128/JVI.01778-18 PMC6401468 30626670

[70] Maier BD, Aguilera LU, Sahle S, Mutz P, Kalra P, Dächert C, et al. Stochastic dynamics of Type-I interferon responses. PLoS Comput Biol. 2022;18(10):e1010623. doi: 10.1371/journal.pcbi.1010623 36269758 PMC9629604

[71] Ivashkiv LB, Donlin LT. Regulation of type I interferon responses. Nat Rev Immunol. 2014;14(1):36–49. doi: 10.1038/nri3581 24362405 PMC4084561

[72] Pestka S, Krause CD, Walter MR. Interferons, interferon-like cytokines, and their receptors. Immunol Rev. 2004;202:8–32. doi: 10.1111/j.0105-2896.2004.00204.x 15546383

[73] Sarasin-Filipowicz M, Oakeley EJ, Duong FHT, Christen V, Terracciano L, Filipowicz W, et al. Interferon signaling and treatment outcome in chronic hepatitis C. Proc Natl Acad Sci U S A. 2008;105(19):7034–9. doi: 10.1073/pnas.0707882105 18467494 PMC2383932

[74] Yoshimura A, Naka T, Kubo M. SOCS proteins, cytokine signalling and immune regulation. Nat Rev Immunol. 2007;7(6):454–65. doi: 10.1038/nri2093 17525754

[75] François-Newton V, Magno de Freitas Almeida G, Payelle-Brogard B, Monneron D, Pichard-Garcia L, Piehler J, et al. USP18-based negative feedback control is induced by type I and type III interferons and specifically inactivates interferon α response. PLoS One. 2011;6(7):e22200. doi: 10.1371/journal.pone.0022200 21779393 PMC3136508

[76] Fenner JE, Starr R, Cornish AL, Zhang J-G, Metcalf D, Schreiber RD, et al. Suppressor of cytokine signaling 1 regulates the immune response to infection by a unique inhibition of type I interferon activity. Nat Immunol. 2006;7(1):33–9. doi: 10.1038/ni1287 16311601

[77] Piganis RAR, De Weerd NA, Gould JA, Schindler CW, Mansell A, Nicholson SE, et al. Suppressor of cytokine signaling (SOCS) 1 inhibits type I interferon (IFN) signaling via the interferon alpha receptor (IFNAR1)-associated tyrosine kinase Tyk2. J Biol Chem. 2011;286(39):33811–8. doi: 10.1074/jbc.M111.270207 21757742 PMC3190811

[78] Boersma S, Rabouw HH, Bruurs LJM, Pavlovič T, van Vliet ALW, Beumer J, et al. Translation and Replication Dynamics of Single RNA Viruses. Cell. 2020;183(7):1930-1945.e23. doi: 10.1016/j.cell.2020.10.019 33188777 PMC7664544

[79] Talemi SR, Bartenschlager M, Schmid B, Ruggieri A, Bartenschlager R, Höfer T. Dengue virus is sensitive to inhibition prior to productive replication. Cell Rep. 2021;37(2):109801. doi: 10.1016/j.celrep.2021.109801 34644578

[80] Rebendenne A, Valadão ALC, Tauziet M, Maarifi G, Bonaventure B, McKellar J, et al. SARS-CoV-2 triggers an MDA-5-dependent interferon response which is unable to control replication in lung epithelial cells. J Virol. 2021;95(8):e02415-20. doi: 10.1128/JVI.02415-20 33514628 PMC8103705

[81] Miorin L, Maestre AM, Fernandez-Sesma A, García-Sastre A. Antagonism of type I interferon by flaviviruses. Biochem Biophys Res Commun. 2017;492(4):587–96. doi: 10.1016/j.bbrc.2017.05.146 28576494 PMC5626595

[82] Deng X, Hackbart M, Mettelman RC, O’Brien A, Mielech AM, Yi G, et al. Coronavirus nonstructural protein 15 mediates evasion of dsRNA sensors and limits apoptosis in macrophages. Proc Natl Acad Sci U S A. 2017;114(21):E4251–60. doi: 10.1073/pnas.1618310114 28484023 PMC5448190

[83] Clayton J, Otter N, Bracci N, Parenti NA, Ye C, Asthana A, et al. Sars-cov-2 nsp15 endoribonuclease antagonizes dsrna-induced antiviral signaling. Proc Natl Acad Sci. 2024;121(15):e2320194121. 10.1073/pnas.2320194121 PMC11009620 38568967

[84] Gao Z, Zhang X, Zhang L, Wu S, Ma J, Wang F, et al. A yellow fever virus NS4B inhibitor not only suppresses viral replication, but also enhances the virus activation of RIG-I-like receptor-mediated innate immune response. PLoS Pathog. 2022;18(1):e1010271. doi: 10.1371/journal.ppat.1010271 35061864 PMC8809586

[85] Hou F, Sun L, Zheng H, Skaug B, Jiang Q-X, Chen ZJ. MAVS forms functional prion-like aggregates to activate and propagate antiviral innate immune response. Cell. 2011;146(3):448–61. doi: 10.1016/j.cell.2011.06.041 21782231 PMC3179916

[86] Brubaker SW, Gauthier AE, Mills EW, Ingolia NT, Kagan JC. A bicistronic MAVS transcript highlights a class of truncated variants in antiviral immunity. Cell. 2014;156(4):800–11. doi: 10.1016/j.cell.2014.01.021 24529381 PMC3959641

[87] Liu S, Cai X, Wu J, Cong Q, Chen X, Li T, et al. Phosphorylation of innate immune adaptor proteins MAVS, STING, and TRIF induces IRF3 activation. Science. 2015;347(6227):aaa2630. doi: 10.1126/science.aaa2630 25636800

[88] Daffis S, Suthar MS, Gale M, Diamond MS. Measure and countermeasure: type i ifn (ifn-*α*/*β*) antiviral response against west nile virus. J Innate Immun. 2009;1(5):435–45. 20375601 10.1159/000226248 PMC2920482

[89] Qing F, Liu Z. Interferon regulatory factor 7 in inflammation, cancer and infection. Front Immunol. 2023;14:1190841. doi: 10.3389/fimmu.2023.1190841 37251373 PMC10213216

[90] Honda K, Yanai H, Negishi H, Asagiri M, Sato M, Mizutani T, et al. IRF-7 is the master regulator of type-I interferon-dependent immune responses. Nature. 2005;434(7034):772–7. doi: 10.1038/nature03464 15800576

[91] Berri F, N’Guyen Y, Callon D, Lebreil A-L, Glenet M, Heng L, et al. Early plasma interferon-β levels as a predictive marker of COVID-19 severe clinical events in adult patients. J Med Virol. 2023;95(1):e28361. doi: 10.1002/jmv.28361 36451263 PMC9877952

[92] Velazquez L, Fellous M, Stark GR, Pellegrini S. A protein tyrosine kinase in the interferon alpha/beta signaling pathway. Cell. 1992;70(2):313–22. doi: 10.1016/0092-8674(92)90105-l 1386289

[93] Prchal-Murphy M, Semper C, Lassnig C, Wallner B, Gausterer C, Teppner-Klymiuk I, et al. TYK2 kinase activity is required for functional type I interferon responses in vivo. PLoS One. 2012;7(6):e39141. doi: 10.1371/journal.pone.0039141 22723949 PMC3377589

[94] Minegishi Y, Saito M, Morio T, Watanabe K, Agematsu K, Tsuchiya S, et al. Human tyrosine kinase 2 deficiency reveals its requisite roles in multiple cytokine signals involved in innate and acquired immunity. Immunity. 2006;25(5):745–55. doi: 10.1016/j.immuni.2006.09.009 17088085

[95] Gonçalves A, Bertrand J, Ke R, Comets E, de Lamballerie X, Malvy D, et al. Timing of Antiviral Treatment Initiation is Critical to Reduce SARS-CoV-2 Viral Load. CPT Pharmacometrics Syst Pharmacol. 2020;9(9):509–14. doi: 10.1002/psp4.12543 32558354 PMC7323384

[96] Sa Ribero M, Jouvenet N, Dreux M, Nisole S. Interplay between SARS-CoV-2 and the type I interferon response. PLoS Pathog. 2020;16(7):e1008737. doi: 10.1371/journal.ppat.1008737 32726355 PMC7390284

[97] Ianevski A, Yao R, Zusinaite E, Lello LS, Wang S, Jo E, et al. Synergistic Interferon-Alpha-Based Combinations for Treatment of SARS-CoV-2 and Other Viral Infections. Viruses. 2021;13(12):2489. doi: 10.3390/v13122489 34960758 PMC8705725

[98] Mesic A, Jackson EK, Lalika M, Koelle DM, Patel RC. Interferon-based agents for current and future viral respiratory infections: A scoping literature review of human studies. PLOS Glob Public Health. 2022;2(4):e0000231. doi: 10.1371/journal.pgph.0000231 36962150 PMC10022196

[99] Chhajer H, Roy R. Rationalised experiment design for parameter estimation with sensitivity clustering. Sci Rep. 2024;14(1):25864. doi: 10.1038/s41598-024-75539-2 39468150 PMC11519581

[100] King CR, Mehle A. Retasking of canonical antiviral factors into proviral effectors. Curr Opin Virol. 2022;56:101271. doi: 10.1016/j.coviro.2022.101271 36242894 PMC10090225

[101] Gonzalez-Orozco M, Rodriguez-Salazar CA, Giraldo MI. The dual role of trim7 in viral infections. Viruses. 2024;16(8):1285. 39205259 10.3390/v16081285 PMC11360163

[102] Sato M, Suemori H, Hata N, Asagiri M, Ogasawara K, Nakao K, et al. Distinct and essential roles of transcription factors IRF-3 and IRF-7 in response to viruses for IFN-alpha/beta gene induction. Immunity. 2000;13(4):539–48. doi: 10.1016/s1074-7613(00)00053-4 11070172

[103] Honda K, Takaoka A, Taniguchi T. Type I interferon [corrected] gene induction by the interferon regulatory factor family of transcription factors. Immunity. 2006;25(3):349–60. doi: 10.1016/j.immuni.2006.08.009 16979567

[104] Zou X, Xiang X, Chen Y, Peng T, Luo X, Pan Z. Understanding inhibition of viral proteins on type I IFN signaling pathways with modeling and optimization. J Theor Biol. 2010;265(4):691–703. doi: 10.1016/j.jtbi.2010.05.001 20553733

