# Supplementary material for: Integrative modelling of innate immune response dynamics during virus infection
Source: PLoS Comput Biol. 2026 Jun 22;22(6):e1014395. doi: 10.1371/journal.pcbi.1014395 (PMC13322630; doi:10.1371/journal.pcbi.1014395)
Supplement: S1 Text — (PDF) [file pcbi.1014395.s001.pdf]

1 Model Description

The model comprises a coupled system of ordinary differential equations that describes the intracellular life cycle of a positive-sense RNA virus and the host's innate immune response. It is a mechanistic, ODE-based framework designed to simulate the progression of infection within a single cell, capturing viral replication dynamics, innate immune sensing, interferon production and signaling, and downstream antiviral effector responses. Although several components of the model adopt structures previously introduced in published mechanistic models [1, 2], we restate the full formulation here to ensure clarity and to make the presentation self-contained. The model comprises 75 molecular species (Supplementary Table S1) and explicitly incorporates viral replication processes, immune signaling cascades, transcriptional feedback mechanisms, and viral antagonism of host defenses.

1.1 Viral life cycle module

Viral entry is modeled as a first-order process governed by the rate constant $k_{\text{en},V}$, resulting in depletion of extracellular virus (V_0, Eq. (S3)) and accumulation of internalized virus (V_I, Eq. (S4)). Internalized virus can undergo uncoating at rate $k_{f,V}$, releasing viral genomic RNA into the cytoplasm, or be degraded at rate μ_{V_I} (Eq. (S4)). Upon uncoating, viral RNA contributes to the cytoplasmic positive-sense RNA pool (R_{cyt}, Eq. (S5)), which serves as the central substrate for translation, replication, and virion assembly. Cytoplasmic viral RNA is translated into structural (P_S, Eq. (S6)) and non-structural (P_{NS}, Eq. (S7)) viral proteins at rate $k_{t,V}$, while also being consumed during virion assembly at rate $k_{a,V}$ and degraded at rate $\mu_{r,V}$ (Eq. (S5)).

Viral replication is assumed to occur in specialized compartmentalized replication structures. Formation of replication compartments depends on the availability of cytoplasmic viral RNA (R_{cyt}) and non-structural proteins (P_{NS}) and proceeds at rate $k_{c,V}$, subject to a finite carrying capacity N_C and a time-dependent maturation function $f_{CM}(t)$ (Eqs. (S1)–(S2)) that captures the gradual establishment of functional replication organelles. The normalized growth of vesicular compartments, f_{CM}, is modeled using a Weibull function, where τ parameterizes the time scale of the structural manifestation of CMs, and n defines the steepness of the function [1]. As replication compartments form, viral RNA and non-structural proteins are sequestered into replication complexes (RC_{CM}), whose dynamics are governed by Eq. (S8). Replication complexes generate newly synthesized positive-sense viral RNA within compartments at rate $k_{r,V}$, producing compartmentalized viral RNA (R_{CM}, Eq. (S9)), which can be exported back into the cytoplasm at rate $k_{e,V}$ to replenish R_{cyt} (Eq. (S5)).

Replication complex turnover produces double-stranded viral RNA intermediates (RNA_{ds}), generated at a rate $k_{l,V}$ (Eq. (S10)), which represent a key pathogen-associated molecular pattern. Cytoplasmic viral RNA is also consumed during virion assembly, which proceeds through the interaction of cytoplasmic RNA (R_{cyt}) and structural proteins (P_S), with each virion requiring nSP structural protein units (Eq. (S6)). Newly assembled virions accumulate as extracellular virus (V_T, Eq. (S11)) and are cleared at rate $\mu_{V,V}$.

Antiviral interferon-stimulated genes (ISGav) inhibit multiple stages of the viral life cycle, including viral entry ($k_{\text{en},V}$), uncoating ($k_{f,V}$), RNA replication ($k_{r,V}$), protein translation ($k_{t,V}$), virion assembly ($k_{a,V}$), and RNA stability ($\mu_{r,V}$), with inhibition strength determined by $I_{V,N}$ (Eqs. (S3)–(S11)). These effects are implemented through multiplicative scaling of kinetic rates, allowing the antiviral state of the cell to dynamically suppress viral replication.

$$f_{CM} = 1 - \exp\left(-\left(\frac{t}{\tau}\right)^4\right) \tag{S1}$$

$$RC_{\text{form\_rate}} = k_{c,V} \cdot R_{\text{cyt}} \cdot P_{NS} \cdot \left(f_{CM} - \frac{RC_{CM}}{N_C}\right) \tag{S2}$$

$$\frac{dV_0}{dt} = -\frac{k_{\text{en, V}}}{1 + I_{V,N} \cdot \text{ISGav}} \cdot V_0 \tag{S3}$$

$$\frac{dV_I}{dt} = \frac{k_{\text{en, V}}}{1 + I_{V,N} \cdot \text{ISGav}} \cdot V_0 - \frac{k_{f,V}}{1 + I_{V,N} \cdot \text{ISGav}} \cdot V_I - \mu_{V_I} \cdot V_I \tag{S4}$$

$$\frac{dR_{\text{cyt}}}{dt} = k_{e,V} \cdot R_{\text{CM}} - \frac{k_{a,V}}{1 + I_{V,N} \cdot \text{ISGav}} \cdot P_S \cdot R_{\text{cyt}} - \mu_{r,V} \cdot (1 + I_{V,N} \cdot \text{ISGav}) \cdot R_{\text{cyt}} - \text{RC}_{\text{form\_rate}} + \frac{k_{f,V}}{1 + I_{V,N} \cdot \text{ISGav}} \cdot V_I \tag{S5}$$

$$\frac{dP_S}{dt} = \frac{k_{t,V}}{1 + I_{V,N} \cdot \text{ISGav}} \cdot R_{\text{cyt}} - \frac{k_{a,V}}{1 + I_{V,N} \cdot \text{ISGav}} \cdot \text{nSP} \cdot P_S \cdot R_{\text{cyt}} - \mu_{p,V} \cdot (1 + I_{V,N} \cdot \text{ISGav}) \cdot P_S \tag{S6}$$

$$\frac{dP_{\text{NS}}}{dt} = \frac{k_{t,V}}{1 + I_{V,N} \cdot \text{ISGav}} \cdot R_{\text{cyt}} - \text{RC}_{\text{form\_rate}} - \mu_{p,V} \cdot (1 + I_{V,N} \cdot \text{ISGav}) \cdot P_{\text{NS}} \tag{S7}$$

$$\frac{d\text{RC}_{\text{CM}}}{dt} = \text{RC}_{\text{form\_rate}} - k_{\text{l,V}} \cdot \text{RC}_{\text{CM}} \tag{S8}$$

$$\frac{dR_{\text{CM}}}{dt} = \frac{k_{r,V}}{1 + I_{V,N} \cdot \text{ISGav}} \cdot \text{RC}_{\text{CM}} - k_{e,V} \cdot R_{\text{CM}} \tag{S9}$$

$$\frac{d\text{RNA}_{\text{ds}}}{dt} = k_{\text{l,V}} \cdot \text{RC}_{\text{CM}} + b_{\text{RIGI}} \cdot \text{aRIGI} - \mu_{r,V} \cdot (1 + I_{V,N} \cdot \text{ISGav}) \cdot \text{RNA}_{\text{ds}} - k_{\text{RIGI}} \cdot \text{RIGI} \cdot \text{RNA}_{\text{ds}} \tag{S10}$$

$$\frac{dV_T}{dt} = \frac{k_{a,V}}{1 + I_{V,N} \cdot \text{ISGav}} \cdot P_S \cdot R_{\text{cyt}} - \mu_{V,V} \cdot V_T \tag{S11}$$

1.2 Innate Immune Sensing: RIG-I signaling

Viral double-stranded RNA (RNA_{ds}, Eq. (S10)) is sensed by the cytosolic pattern recognition receptor RIG-I (RIGI, Eq. (S12)) [3]. Binding of RNA_{ds} to RIG-I induces conformational changes and oligomerization, leading to the formation of activated RIG-I signaling complexes (aRIGI, Eq. (S13)) at rate k_{RIGI} [4]. Free RIG-I is maintained through basal synthesis ($k_{\text{RIGI\_synt}}$) and turnover (μ_{RIGI}), while its abundance is further amplified through interferon-dependent transcriptional upregulation, modeled via ISG-driven expression with rate $k_{\text{t,ISG RNA}}$ [5].

Activated RIG-I recruits and activates the mitochondrial antiviral signaling protein MAVS, generating active MAVS signaling platforms (aMAVS, Eq. (S15)) [6, 7]. MAVS activation is modeled as a reversible process, reflecting both signal amplification and regulatory shutoff mechanisms. Viral non-structural proteins antagonize MAVS activation through the factor $V_{I,N} \cdot P_{NS}$, which reduces the effective activation rate k_{MAVS} (Eqs. (S14)–(S15)). This term represents viral protease- or scaffold-mediated disruption of MAVS signaling complexes, a conserved immune evasion strategy employed by many positive-sense RNA viruses [8, 9].

Active MAVS initiates downstream kinase signaling by promoting phosphorylation of TBK1 and IKKε. These kinases are represented as inactive–active pools undergoing MAVS-dependent activation at rate $k_{\text{IKKe-TBK1}}$ and basal deactivation at rate b_{KINASE} (Eqs. (S16)–(S19)) [10, 11]. Activated TBK1 and IKKε phosphorylate the interferon regulatory factors IRF3 and IRF7 (Eqs. (S20)–(S21)), generating phosphorylated IRF3 (pIRF3) and phosphorylated IRF7 (pIRF7), which translocate to the nucleus [11].

IRF7 is explicitly included in the model to capture the temporal amplification and diversification of the interferon response. While IRF3 mediates the initial wave of interferon induction, IRF7 is itself an interferon-stimulated gene whose expression is strongly induced following early interferon signaling [12, 13]. Phosphorylated IRF7 is therefore essential for sustained and amplified type I interferon production as demonstrated experimentally in knockout and viral infection studies [14, 15]. The inclusion of IRF7 allows the model to reproduce the

transition from an early, IRF3-dominated response to a later, IRF7-driven amplification phase, which is critical for robust antiviral immunity.

Viral antagonism reduces IRF3 and IRF7 phosphorylation efficiency through the $V_{I,N} \cdot P_{NS}$-dependent inhibitory term, representing viral interference with TBK1/IKKε activity or transcription factor access [9, 16].

In parallel to IRF activation, MAVS signaling activates the canonical IKK complex (Eqs. (S24)–(S25)), leading to phosphorylation and nuclear translocation of NF-κB following release from IκBα [2, 17]. NF-κB dynamics explicitly include cytoplasmic sequestration, IKK-dependent activation, nuclear import and export, dephosphorylation, and negative feedback through NF-κB–dependent synthesis of IκBα (Eqs. (S26)–(S30)) [2].

Activated transcription factors regulate interferon gene expression in a subtype-specific manner. IFN-β transcription requires cooperative binding of IRF3/IRF7 and NF-κB to the IFN-β enhanceosome (Eq. (S31)) [18]. IFN-α transcription is driven predominantly by IRF7, with contributions from IRF3, consistent with the role of IRF7 as the master regulator of IFN-α subtype expression (Eq. (S32)) [19]. IFN-λ transcription is modeled as primarily IRF3-dependent (Eq. (S33)) [2].

Interferon mRNAs are translated into cytoplasmic interferon pools and subsequently secreted (Eqs. (S34)–(S35)), enabling autocrine signaling. The combined structure of IRF3-, IRF7-, and NF-κB–dependent regulation allows the model to capture both the timing and magnitude of subtype-specific interferon responses. Viral antagonism, represented by $V_{I,N}$, suppresses MAVS activation and IRF phosphorylation, thereby shaping the balance between viral replication and innate immune activation.

$$\frac{d\mathrm{RIGI}}{dt} = k_{\mathrm{RIGI\_synt}} - \mu_{\mathrm{RIGI}} \cdot \mathrm{RIGI} + b_{\mathrm{RIGI}} \cdot \mathrm{aRIGI} - k_{\mathrm{RIGI}} \cdot \mathrm{RIGI} \cdot \mathrm{RNA_{ds}} + k_{\mathrm{t,\ ISG\ RNA}} \cdot \mathrm{ISGav_m} \tag{S12}$$

$$\frac{d\mathrm{aRIGI}}{dt} = k_{\mathrm{RIGI}} \cdot \mathrm{RIGI} \cdot \mathrm{RNA_{ds}} - \mu_{\mathrm{RIGI}} \cdot \mathrm{aRIGI} - b_{\mathrm{RIGI}} \cdot \mathrm{aRIGI} \tag{S13}$$

$$\frac{d\mathrm{MAVS}}{dt} = b_{\mathrm{MAVS}} \cdot \mathrm{aMAVS} - \mathrm{MAVS} \cdot \mathrm{aRIGI} \cdot \left(\frac{k_{\mathrm{MAVS}}}{1 + V_{I,N} \cdot P_{\mathrm{NS}}} \right) \tag{S14}$$

$$\frac{d\mathrm{aMAVS}}{dt} = \mathrm{MAVS} \cdot \mathrm{aRIGI} \cdot \left(\frac{k_{\mathrm{MAVS}}}{1 + V_{I,N} \cdot P_{\mathrm{NS}}} \right) - b_{\mathrm{MAVS}} \cdot \mathrm{aMAVS} \tag{S15}$$

$$\frac{d\mathrm{IKKe}}{dt} = b_{\mathrm{KINASE}} \cdot \mathrm{aIKKe} - \mathrm{IKKe} \cdot \mathrm{aMAVS} \cdot k_{\mathrm{IKKe\text{-}TBK1}} \tag{S16}$$

$$\frac{d\mathrm{aIKKe}}{dt} = \mathrm{IKKe} \cdot \mathrm{aMAVS} \cdot k_{\mathrm{IKKe\text{-}TBK1}} - b_{\mathrm{KINASE}} \cdot \mathrm{aIKKe} \tag{S17}$$

$$\frac{d\mathrm{TBK1}}{dt} = b_{\mathrm{KINASE}} \cdot \mathrm{aTBK1} - \mathrm{TBK1} \cdot \mathrm{aMAVS} \cdot k_{\mathrm{IKKe\text{-}TBK1}} \tag{S18}$$

$$\frac{d\mathrm{aTBK1}}{dt} = \mathrm{TBK1} \cdot \mathrm{aMAVS} \cdot k_{\mathrm{IKKe\text{-}TBK1}} - b_{\mathrm{KINASE}} \cdot \mathrm{aTBK1} \tag{S19}$$

$$\frac{d\mathrm{IRF3}}{dt} = \mathrm{Vn2c} \cdot b_{\mathrm{IRF3}} \cdot \mathrm{pIRF3} - \mathrm{IRF3} \cdot k_{\mathrm{IRF3\text{-}IKKe\text{-}TBK1}} \cdot (\mathrm{pIKKe} + \mathrm{aTBK1}) \cdot \left(\frac{1}{1 + V_{I,N} \cdot P_{\mathrm{NS}}} \right) \tag{S20}$$

$$\frac{d\mathrm{pIRF3}}{dt} = (\mathrm{IRF3} \cdot \mathrm{Vc2n} \cdot k_{\mathrm{IRF3\text{-}IKKe\text{-}TBK1}} \cdot (\mathrm{pIKKe} + \mathrm{aTBK1})) \cdot \left(\frac{1}{1 + V_{I,N} \cdot P_{\mathrm{NS}}} \right) - b_{\mathrm{IRF3}} \cdot \mathrm{pIRF3} \tag{S21}$$

$$\frac{d\text{IRF7}}{dt} = k_{71}\cdot\text{Vn2c}\cdot\text{pIRF7}-\text{IRF7}\cdot k_{\text{IRF3-IKKe-TBK1}}\cdot(\text{pIKKe}+\text{aTBK1})\cdot\left(\frac{1}{1+V_{I,N}\cdot P_{NS}}\right)+k_{79}\cdot\text{IRF7\_m}-\mu_{\text{IRF7}}\cdot\text{IRF7} \tag{S22}$$

$$\frac{d\text{pIRF7}}{dt} = \text{IRF7}\cdot\text{Vc2n}\cdot k_{\text{IRF3-IKKe-TBK1}}\cdot(\text{pIKKe}+\text{aTBK1})\cdot\left(\frac{1}{1+V_{I,N}\cdot P_{NS}}\right)-k_{71}\cdot\text{pIRF7} \tag{S23}$$

$$\frac{d\text{IKK}}{dt} = \text{aIKK}\cdot b_{\text{KINASE}}-\text{IKK}\cdot\text{aMAVS}\cdot k_{\text{IKK}} \tag{S24}$$

$$\frac{d\text{aIKK}}{dt} = \text{IKK}\cdot\text{aMAVS}\cdot k_{\text{IKK}}-\text{aIKK}\cdot b_{\text{KINASE}} \tag{S25}$$

$$\frac{d\text{NFkB\_IkBa}_c}{dt} = \text{IkBac}\cdot\text{NFkBc}\cdot k_{\text{inh\_p65}}-\text{NFkB\_IkBac}\cdot\text{aIKK}\cdot k_{\text{act}} \tag{S26}$$

$$\frac{dp\text{NFkB}_n}{dt} = \text{Vc2n}\cdot\text{aIKK}\cdot k_{\text{act}}\cdot(\text{NFkBc}+\text{NFkB\_IkBac})-k_{\text{deph}}\cdot p\text{NFkBn} \tag{S27}$$

$$\frac{d\text{NFkB}_n}{dt} = k_{\text{deph}}\cdot p\text{NFkBn}-\text{NFkBn}\cdot k_{\text{transp\_NFkB}} \tag{S28}$$

$$\frac{d\text{NFkB}_c}{dt} = \text{NFkBn}\cdot\text{Vn2c}\cdot k_{\text{transp\_NFkB}}-\text{IkBac}\cdot\text{NFkBc}\cdot k_{\text{inh\_p65}}-\text{NFkBc}\cdot\text{aIKK}\cdot k_{\text{act}} \tag{S29}$$

$$\frac{d\text{IkBa}_c}{dt} = k_{\text{expr\_IkBa}}\cdot(\text{NFkBn}+p\text{NFkBn})-\text{IkBac}\cdot\mu_{\text{IkBa}}-\text{IkBac}\cdot\text{NFkBc}\cdot k_{\text{inh\_p65}} \tag{S30}$$

$$\frac{d\text{IFNb\_}m}{dt} = \text{B}_O\cdot\text{Vn2c}\cdot k_{\text{TFBS\_IFNb}}-\text{IFNb}_m\cdot\mu_{\text{m\_IFNb}}+\text{Vn2c}\cdot k_{\text{m\_IFNb}}\cdot(p\text{IRF3}+p\text{IRF7})\cdot(\text{NFkBn}+p\text{NFkBn}) \tag{S31}$$

$$\frac{d\text{IFNa\_}m}{dt} = \text{B}_O\cdot\text{Vn2c}\cdot k_{\text{TFBS\_IFNa}}-\text{IFNa}_m\cdot\mu_{\text{m\_IFNa}}+\text{Vn2c}\cdot k_{\text{m\_IFNa}}\cdot(p\text{IRF3}+p\text{IRF7}) \tag{S32}$$

$$\frac{d\text{IFNl\_}m}{dt} = \text{B}_O\cdot\text{Vn2c}\cdot k_{\text{TFBS\_IFNl}}-\text{IFNl}_m\cdot\mu_{\text{m\_IFNl}}+\text{Vn2c}\cdot k_{\text{m\_IFNl}}\cdot p\text{IRF3} \tag{S33}$$

1.3 JAK-STAT signaling

Cytoplasmic type I interferons (IFN_c, Eq. (S34)) and type III interferon (IFNl_c, Eq. (S35)) are secreted into a shared extracellular interferon pool (IFNex, Eq. (S36)), which drives autocrine signaling in infected cells and paracrine signaling in neighboring cells. IFN-α and IFN-β share the heterodimeric type I interferon receptor, composed of IFNAR1 and IFNAR2, and are therefore treated jointly at the levels of cytoplasmic accumulation and secretion. In contrast, IFN-λ signals through a distinct receptor complex and is modeled as a parallel but independent production and degradation process, reflecting its specialized role at epithelial barriers and its restricted receptor expression pattern [20].

Extracellular interferon binding induces the formation of interferon receptor complexes involving IFNAR1, IFNAR2, and the associated kinases JAK1 and TYK2 (Eqs. (S37)–(S44)). Receptor dynamics explicitly include ligand binding, adaptor and receptor complex assembly (ARC, RJC, RTKC), kinase recruitment, receptor internalization, and recycling. These processes collectively regulate both the magnitude and duration of downstream signaling. In addition to canonical receptor activation, the model incorporates multiple experimentally

described mechanisms of negative regulation at the receptor level, including ISG-dependent inhibition of receptor activation, enhanced receptor degradation, and suppression of adaptor complex formation [21, 22, 23]. These regulatory effects are mediated by a lumped class of regulatory interferon-stimulated genes, denoted ISGn, acting with strength $I_{I,N}$, and represent the action of suppressor of cytokine signaling (SOCS) proteins, ubiquitin-specific peptidases (USP), and other feedback inhibitors that restrain interferon responsiveness.

Activated receptor complexes phosphorylate STAT1 and STAT2, which subsequently form intermediate cytoplasmic complexes with IRF9 and with each other (Eqs. (S47)–(S56)). These interactions result in the formation of the interferon-stimulated gene factor 3 complex (ISGF3; Eqs. (S57)–(S58)), consisting of phosphorylated STAT1, phosphorylated STAT2, and IRF9 [24, 25]. ISGF3 undergoes nucleocytoplasmic shuttling and binds interferon-stimulated response elements at promoter sites, inducing transcription of antiviral interferon-stimulated genes (ISGav), regulatory ISGs (ISGn), and signaling components including RIG-I, IRF7, and IRF9 (Eqs. (S71)–(S77)) [5]. For modeling simplicity, antiviral ISGs are lumped into a single effective variable ISGav, which captures the collective antiviral activity exerted across multiple stages of the viral life cycle.

The JAK–STAT module incorporates multiple layers of signal attenuation and negative feedback. Cytoplasmic and nuclear phosphatases dephosphorylate STAT complexes and ISGF3 (Eqs. (S63)–(S69)), limiting signal persistence. Protein inhibitors of activated STATs (PIAS) reduce ISGF3 transcriptional activity by inhibiting DNA binding and promoting complex dissociation (Eq. (S62)) [26]. Additional attenuation arises from the nuclear sequestration of signaling components and ISGn-mediated receptor-level inhibition, which together ensure tight control of interferon responsiveness and prevent excessive or chronic signaling.

Viral antagonism, represented by $V_{I,N}$, suppresses receptor-associated STAT activation and downstream complex formation, reflecting viral interference with JAK–STAT signaling through degradation or inhibition of STATs, receptor components, or adaptor proteins. This suppression limits ISGF3 accumulation and downstream ISG induction, thereby modulating the balance between antiviral defense and productive viral replication.

$$\frac{d\mathrm{IFN}_c}{dt} = (\mathrm{IFNa}_m + \mathrm{IFNb}_m) \cdot k_{\mathrm{IFN}} - k_s \cdot \mathrm{IFN}_c - \mu_{\mathrm{IFN}} \cdot \mathrm{IFN}_c \tag{S34}$$

$$\frac{d\mathrm{IFNl}_c}{dt} = \mathrm{IFNl}_m \cdot k_{\mathrm{trans\_IFNl}} - \mathrm{IFNl}_c \cdot \mu_{\mathrm{IFNl}} \tag{S35}$$

$$\frac{d\mathrm{IFNex}}{dt} = \mathrm{IFNARd} \cdot k_6 + k_s \cdot \mathrm{IFN}_c - \mathrm{IFNex} \cdot \mathrm{RJC} \cdot \mathrm{RTKC} \cdot k_5 \tag{S36}$$

$$\frac{d\mathrm{JAK}}{dt} = \mathrm{ARC} \cdot k_{32} + \mathrm{RJC} \cdot k_4 + \mathrm{ARC} \cdot I_{I,N} \cdot \mathrm{ISGn} \cdot k_{69} - \mathrm{JAK} \cdot \mathrm{IFNAR2} \cdot k_3 \tag{S37}$$

$$\frac{d\mathrm{RJC}}{dt} = \mathrm{IFNARd} \cdot k_6 - \mathrm{RJC} \cdot k_4 + \mathrm{JAK} \cdot \mathrm{IFNAR2} \cdot k_3 - \mathrm{IFNex} \cdot \mathrm{RJC} \cdot \mathrm{RTKC} \cdot k_5 \tag{S38}$$

$$\frac{d\mathrm{TYK}}{dt} = \mathrm{ARC} \cdot k_{32} + \mathrm{RTKC} \cdot k_2 + \mathrm{ARC} \cdot I_{I,N} \cdot \mathrm{ISGn} \cdot k_{69} - \mathrm{IFNAR1} \cdot \mathrm{TYK} \cdot k_1 \tag{S39}$$

$$\frac{d\mathrm{RTKC}}{dt} = \mathrm{IFNARd} \cdot k_6 - \mathrm{RTKC} \cdot k_2 + \mathrm{IFNAR1} \cdot \mathrm{TYK} \cdot k_1 - \mathrm{IFNex} \cdot \mathrm{RJC} \cdot \mathrm{RTKC} \cdot k_5 \tag{S40}$$

$$\frac{d\mathrm{IFNAR1}}{dt} = \mathrm{ARC} \cdot k_{32} + \mathrm{RTKC} \cdot k_2 + \mathrm{ARC} \cdot I_{I,N} \cdot \mathrm{ISGn} \cdot k_{69} - \mathrm{IFNAR1} \cdot \mathrm{TYK} \cdot k_1 - I_{I,N} \cdot \mathrm{degRecISGn} \cdot \mathrm{IFNAR1} \cdot \mathrm{ISGn} \tag{S41}$$

$$\frac{d\mathrm{IFNAR2}}{dt} = \mathrm{ARC} \cdot k_{32} + \mathrm{RJC} \cdot k_4 + \mathrm{ARC} \cdot I_{I,N} \cdot \mathrm{ISGn} \cdot k_{69} - \mathrm{JAK} \cdot \mathrm{IFNAR2} \cdot k_3 - I_{I,N} \cdot \mathrm{degRecISGn} \cdot \mathrm{IFNAR2} \cdot \mathrm{ISGn} \tag{S42}$$

$$\frac{d\text{IFNARd}}{dt} = \text{ARC} \cdot k_{34} - \text{IFNARd} \cdot (k_6 + \left(\frac{k_7}{1 + I_{I,N} \cdot \text{kinhISGn} \cdot \text{ISGn}}\right)) + \text{IFNex} \cdot \text{RJC} \cdot \text{RTKC} \cdot k_5 \quad \text{(S43)}$$

$$\begin{aligned}\frac{d\text{ARC}}{dt} = \text{ARC\_STAT2c} \cdot k_{10} - \text{ARC} \cdot k_{34} - \text{ARC} \cdot k_{32} + \text{ARC\_STAT12} \cdot k_{13} \cdot \left(\frac{1}{1 + V_{I,N} \cdot P_{NS}}\right) \\ + \text{IFNARd} \cdot \left(\frac{k_7}{1 + I_{I,N} \cdot \text{kinhISGn} \cdot \text{ISGn}}\right) - \text{ARC} \cdot I_{I,N} \cdot \text{ISGn} \cdot k_{69} - \text{ARC} \cdot \text{STAT2c} \cdot k_9 \\ - \text{ARC} \cdot \text{STAT2c\_IRF9} \cdot k_8 - I_{I,N} \cdot \text{degARCISGn} \cdot \text{ISGn} \cdot \text{ARC}\end{aligned} \quad \text{(S44)}$$

$$\begin{aligned}\frac{d\text{ARC-STAT2}_c}{dt} = \text{ARC-STAT12}_c \cdot k_{12} - \text{ARC-STAT2}_c \cdot k_{10} + \text{ARC} \cdot \text{STAT2}_c \cdot k_9 \\ + \text{ARC} \cdot \text{STAT2-IRF9}_c \cdot k_8 - \text{ARC-STAT2}_c \cdot \text{STAT1}_c \cdot k_{11}\end{aligned} \quad \text{(S45)}$$

$$\frac{d\text{ARC-STAT12}_c}{dt} = \text{ARC-STAT2}_c \cdot \text{STAT1}_c \cdot k_{11} - \text{ARC-STAT12}_c \cdot k_{13} \cdot \left(\frac{1}{1 + V_{I,N} \cdot P_{NS}}\right) - \text{ARC-STAT12}_c \cdot k_{12} \quad \text{(S46)}$$

$$\begin{aligned}\frac{d\text{STAT1}_c}{dt} = \text{ARC-STAT12}_c \cdot k_{12} + \text{ISGF3-CP} \cdot k_{40} - \text{STAT1}_c \cdot k_{56} + \text{PSC-CP} \cdot k_{43} \\ - \text{ARC-STAT2}_c \cdot \text{STAT1}_c \cdot k_{11} + \text{STAT1}_n \cdot \text{Vn2c} \cdot k_{57}\end{aligned} \quad \text{(S47)}$$

$$\begin{aligned}\frac{d\text{STAT2}_c}{dt} = \text{ARC-STAT2}_c \cdot k_{10} + \text{ISGF3-CP} \cdot k_{40} - \text{STAT2}_c \cdot k_{58} + \text{STAT2-IRF9}_c \cdot (k_{36} + k_{61}) \\ + \text{PSC-CP} \cdot k_{43} - \text{ARC} \cdot \text{STAT2}_c \cdot k_9 - \text{IRF9}_c \cdot \text{STAT2}_c \cdot k_{60} + \text{STAT2}_n \cdot \text{Vn2c} \cdot k_{59}\end{aligned} \quad \text{(S48)}$$

$$\begin{aligned}\frac{d\text{STAT1}_n}{dt} = \text{ISGF3-NP} \cdot k_{49} + \text{B}_O\text{\_NP} \cdot k_{52} - \text{STAT1}_n \cdot k_{57} + \text{PSC-NP} \cdot k_{46} \\ + \text{STAT1}_c \cdot \text{Vc2n} \cdot k_{56}\end{aligned} \quad \text{(S49)}$$

$$\begin{aligned}\frac{d\text{STAT2}_n}{dt} = \text{ISGF3-NP} \cdot k_{49} + \text{B}_O\text{\_NP} \cdot k_{52} - \text{STAT2}_n \cdot k_{59} + \text{STAT2-IRF9}_n \cdot k_{37} + \text{STAT2-IRF9}_n \cdot k_{63} \\ + \text{PSC-NP} \cdot k_{46} - \text{IRF9}_n \cdot \text{STAT2}_n \cdot k_{62} + \text{STAT2}_c \cdot \text{Vc2n} \cdot k_{58}\end{aligned} \quad \text{(S50)}$$

$$\begin{aligned}\frac{d\text{IRF9}_c}{dt} = k_{27} - \text{IRF9}_c \cdot (k_{29} + k_{66}) + \text{ISGF3}_c \cdot k_{15} + \text{ISGF3-CP} \cdot k_{40} + \text{STAT2-IRF9}_c \cdot k_{61} + k_{70} \cdot \text{IRF9\_m}_c \\ + \text{ARC} \cdot \text{STAT2-IRF9}_c \cdot k_8 - \text{IRF9}_c \cdot \text{STAT2}_c \cdot k_{60} - \text{IRF9}_c \cdot \text{PSC}_c \cdot k_{14} + \text{IRF9}_n \cdot \text{Vn2c} \cdot k_{67}\end{aligned} \quad \text{(S51)}$$

$$\begin{aligned}\frac{d\text{IRF9}_n}{dt} = \text{ISGF3}_n \cdot k_{21} - \text{IRF9}_n \cdot k_{67} - \text{IRF9}_n \cdot k_{35} + \text{ISGF3-NP} \cdot k_{49} + \text{B}_O\text{\_NP} \cdot k_{52} + \text{STAT2-IRF9}_n \cdot k_{63} \\ - \text{IRF9}_n \cdot \text{STAT2}_n \cdot k_{62} - \text{IRF9}_n \cdot \text{PSC}_n \cdot k_{20} + \text{IRF9}_c \cdot \text{Vc2n} \cdot k_{66}\end{aligned} \quad \text{(S52)}$$

$$\frac{d\text{PSC}_c}{dt} = \text{ARC-STAT12}_c \cdot k_{13} \cdot \left(\frac{1}{1+V_{I,N}\cdot P_{\text{NS}}}\right) + \text{ISGF3}_c \cdot k_{15} - \text{PSC}_c \cdot k_{18} + \text{PSC-CP} \cdot k_{42} - \text{CP} \cdot \text{PSC}_c \cdot k_{41} - \text{IRF9}_c \cdot \text{PSC}_c \cdot k_{14} + \text{PSC}_n \cdot \text{Vn2c} \cdot k_{19} \tag{S53}$$

$$\frac{d\text{PSC}_n}{dt} = \text{ISGF3}_n \cdot k_{21} - \text{PSC}_n \cdot k_{19} + \text{PSC-NP} \cdot k_{45} - \text{IRF9}_n \cdot \text{PSC}_n \cdot k_{20} - \text{NP} \cdot \text{PSC}_n \cdot k_{44} + \text{PSC}_c \cdot \text{Vc2n} \cdot k_{18} \tag{S54}$$

$$\frac{d\text{STAT2-IRF9}_n}{dt} = \text{IRF9}_n \cdot \text{STAT2}_n \cdot k_{62} - \text{STAT2-IRF9}_n \cdot k_{63} - \text{STAT2-IRF9}_n \cdot k_{65} - \text{STAT2-IRF9}_n \cdot k_{37} + \text{STAT2-IRF9}_c \cdot \text{Vc2n} \cdot k_{64} \tag{S55}$$

$$\frac{d\text{STAT2-IRF9}_c}{dt} = \text{IRF9}_c \cdot \text{STAT2}_c \cdot k_{60} - \text{STAT2-IRF9}_c \cdot (k_{61} + k_{64}) - \text{ARC} \cdot \text{STAT2-IRF9}_c \cdot k_8 - \text{STAT2-IRF9}_c \cdot k_{36} + \text{STAT2-IRF9}_n \cdot \text{Vn2c} \cdot k_{65} \tag{S56}$$

$$\frac{d\text{ISGF3}_c}{dt} = \text{ISGF3-CP} \cdot k_{39} - \text{ISGF3}_c \cdot (k_{16} + k_{15}) - \text{CP} \cdot \text{ISGF3}_c \cdot k_{38} + \text{IRF9}_c \cdot \text{PSC}_c \cdot k_{14} + \text{ISGF3}_n \cdot \text{Vn2c} \cdot k_{17} \tag{S57}$$

$$\frac{d\text{ISGF3}_n}{dt} = \text{ISGF3-NP} \cdot k_{48} - \text{ISGF3}_n \cdot k_{21} - \text{ISGF3}_n \cdot k_{17} + \text{B}_O \cdot k_{23} + \text{PIAS-ISGF3} \cdot k_{54} - \text{ISGF3}_n \cdot \text{NP} \cdot k_{47} - \text{ISGF3}_n \cdot \text{B}_U \cdot k_{22} - \text{ISGF3}_n \cdot \text{PIAS} \cdot k_{53} + \text{IRF9}_n \cdot \text{PSC}_n \cdot k_{20} + \text{ISGF3}_c \cdot \text{Vc2n} \cdot k_{16} \tag{S58}$$

$$\frac{d\text{B}_U}{dt} = \text{B}_O \cdot k_{23} + \text{B}_O\text{\_NP} \cdot k_{52} - \text{ISGF3}_n \cdot \text{B}_U \cdot k_{22} \tag{S59}$$

$$\frac{d\text{B}_O}{dt} = \text{B}_O\text{\_NP} \cdot k_{51} - \text{B}_O \cdot k_{23} + \text{ISGF3}_n \cdot \text{B}_U \cdot k_{22} - \text{NP} \cdot \text{B}_O \cdot k_{50} \tag{S60}$$

$$\frac{d\text{PIAS}}{dt} = \text{PIAS-ISGF3} \cdot k_{54} - \text{ISGF3}_n \cdot \text{PIAS} \cdot k_{53} \tag{S61}$$

$$\frac{d\text{PIAS-ISGF3}}{dt} = \text{ISGF3}_n \cdot \text{PIAS} \cdot k_{53} - \text{PIAS-ISGF3} \cdot k_{54} \tag{S62}$$

$$\frac{d\text{CP}}{dt} = \text{ISGF3-CP} \cdot k_{39} + \text{ISGF3-CP} \cdot k_{40} + \text{PSC-CP} \cdot k_{42} + \text{PSC-CP} \cdot k_{43} - \text{CP} \cdot \text{ISGF3}_c \cdot k_{38} - \text{CP} \cdot \text{PSC}_c \cdot k_{41} \tag{S63}$$

$$\frac{d\text{NP}}{dt} = \text{ISGF3-NP} \cdot k_{48} + \text{ISGF3-NP} \cdot k_{49} + \text{B}_O\text{\_NP} \cdot k_{51} + \text{B}_O\text{\_NP} \cdot k_{52} + \text{PSC-NP} \cdot k_{45} + \text{PSC-NP} \cdot k_{46} - \text{ISGF3}_n \cdot \text{NP} \cdot k_{47} - \text{NP} \cdot \text{B}_O \cdot k_{50} - \text{NP} \cdot \text{PSC}_n \cdot k_{44} \tag{S64}$$

$$\frac{d\text{ISGF3-CP}}{dt} = \text{CP} \cdot \text{ISGF3}_c \cdot k_{38} - \text{ISGF3-CP} \cdot (k_{40} + k_{39}) \tag{S65}$$

$$\frac{d\text{ISGF3-NP}}{dt} = \text{ISGF3}_n \cdot \text{NP} \cdot k_{47} - \text{ISGF3-NP} \cdot k_{49} - \text{ISGF3-NP} \cdot k_{48} \quad \text{(S66)}$$

$$\frac{d\text{PSC-CP}}{dt} = \text{CP} \cdot \text{PSC}_c \cdot k_{41} - \text{PSC-CP} \cdot k_{43} - \text{PSC-CP} \cdot k_{42} \quad \text{(S67)}$$

$$\frac{d\text{PSC-NP}}{dt} = \text{NP} \cdot \text{PSC}_n \cdot k_{44} - \text{PSC-NP} \cdot k_{46} - \text{PSC-NP} \cdot k_{45} \quad \text{(S68)}$$

$$\frac{d\text{B}_O\text{\_NP}}{dt} = \text{NP} \cdot \text{B}_O \cdot k_{50} - \text{B}_O\text{\_NP} \cdot k_{52} - \text{B}_O\text{\_NP} \cdot k_{51} \quad \text{(S69)}$$

$$\frac{d\text{ISGn}}{dt} = k_{transISGn} \cdot \text{ISGn\_m}_c - \text{ISGn} \cdot k_{31} \quad \text{(S70)}$$

$$\frac{d\text{ISGav\_m}}{dt} = \text{B}_O \cdot \text{Vn2c} \cdot k_{\text{ISGav\_m}} - \text{ISGav\_m} \cdot \mu_{\text{ISG RNA}} + \text{Vn2c} \cdot k_{\text{m\_ISGav}} \cdot \text{pIRF3} \quad \text{(S71)}$$

$$\frac{d\text{ISGav}}{dt} = \text{ISGav\_m} \cdot k_{\text{t, ISG RNA}} - \text{ISGav} \cdot \mu_{\text{ISGav}} \quad \text{(S72)}$$

$$\frac{d\text{ISGn\_m}_n}{dt} = k_{72} \cdot \text{B}_O - k_{73} \cdot \text{ISGn\_m}_n \quad \text{(S73)}$$

$$\frac{d\text{IRF9\_m}_n}{dt} = k_{74} \cdot \text{B}_O - k_{75} \cdot \text{IRF9\_m}_n \quad \text{(S74)}$$

$$\frac{d\text{IRF7\_m}}{dt} = k_{76} \cdot \text{B}_O - \left(\frac{\log_e(2)}{\tau_5}\right) \cdot \text{IRF7\_m} \quad \text{(S75)}$$

$$\frac{d\text{ISGn\_m}_c}{dt} = \text{Vn2c} \cdot \text{ISGn\_m}_n \cdot k_{73} - k_{77} \cdot \text{ISGn\_m}_c \quad \text{(S76)}$$

$$\frac{d\text{IRF9\_m}_c}{dt} = \text{Vn2c} \cdot \text{IRF9\_m}_n \cdot k_{75} - k_{78} \cdot \text{IRF9\_m}_c \quad \text{(S77)}$$

References

[1] Harsh Chhajer, Vaseef A Rizvi, and Rahul Roy. Life cycle process dependencies of positive-sense rna viruses suggest strategies for inhibiting productive cellular infection. *J R Soc Interface*, 2021.

[2] Sandy S Burkart, Darius Schweinoch, Jamie Frankish, Carola Sparn, Sandra Wüst, Christian Urban, Marta Merlo, Vladimir G Magalhães, Antonio Piras, Andreas Pichlmair, et al. High-resolution kinetic characterization of the rig-i-signaling pathway and the antiviral response. *Life Science Alliance*, 6(10), 2023.

[3] Mitsutoshi Yoneyama, Mika Kikuchi, Takashi Natsukawa, Noriaki Shinobu, Tadaatsu Imaizumi, Makoto Miyagishi, Kazunari Taira, Shizuo Akira, and Takashi Fujita. The RNA helicase RIG-I has an essential function in double-stranded RNA-induced innate antiviral responses. *Nature Immunology*, 5(7):730–737, July 2004.

[4] Eva Kowalinski, Thomas Lunardi, Andrew A. McCarthy, Jade Louber, Joanna Brunel, Boyan Grigorov, Denis Gerlier, and Stephen Cusack. Structural Basis for the Activation of Innate Immune Pattern-Recognition Receptor RIG-I by Viral RNA. *Cell*, 147(2):423–435, October 2011.

[5] John W. Schoggins, Sam J. Wilson, Maryline Panis, Mary Y. Murphy, Christopher T. Jones, Paul Bieniasz, and Charles M. Rice. A diverse range of gene products are effectors of the type I interferon antiviral response. *Nature*, 472(7344):481–485, April 2011.

[6] Rashu B. Seth, Lijun Sun, Chee-Kwee Ea, and Zhijian J. Chen. Identification and characterization of MAVS, a mitochondrial antiviral signaling protein that activates NF-kappaB and IRF 3. *Cell*, 122(5):669–682, September 2005.

[7] Taro Kawai, Ken Takahashi, Shintaro Sato, Cevayir Coban, Himanshu Kumar, Hiroki Kato, Ken J. Ishii, Osamu Takeuchi, and Shizuo Akira. IPS-1, an adaptor triggering RIG-I- and Mda5-mediated type I interferon induction. *Nature Immunology*, 6(10):981–988, October 2005.

[8] Xiao-Dong Li, Lijun Sun, Rashu B. Seth, Gabriel Pineda, and Zhijian J. Chen. Hepatitis C virus protease NS3/4A cleaves mitochondrial antiviral signaling protein off the mitochondria to evade innate immunity. *Proceedings of the National Academy of Sciences of the United States of America*, 102(49):17717–17722, December 2005.

[9] Lisa Miorin, Ana M. Maestre, Ana Fernandez-Sesma, and Adolfo García-Sastre. ANTAGONISM OF TYPE I INTERFERON BY FLAVIVIRUSES. *Biochemical and biophysical research communications*, 492(4):587–596, October 2017.

[10] Katherine A. Fitzgerald, Sarah M. McWhirter, Kerrie L. Faia, Daniel C. Rowe, Eicke Latz, Douglas T. Golenbock, Anthony J. Coyle, Sha-Mei Liao, and Tom Maniatis. IKK and TBK1 are essential components of the IRF3 signaling pathway. *Nature Immunology*, 4(5):491–496, May 2003. Number: 5.

[11] Sonia Sharma, Benjamin R. tenOever, Nathalie Grandvaux, Guo-Ping Zhou, Rongtuan Lin, and John Hiscott. Triggering the interferon antiviral response through an IKK-related pathway. *Science (New York, N.Y.)*, 300(5622):1148–1151, May 2003.

[12] M. Sato, H. Suemori, N. Hata, M. Asagiri, K. Ogasawara, K. Nakao, T. Nakaya, M. Katsuki, S. Noguchi, N. Tanaka, and T. Taniguchi. Distinct and essential roles of transcription factors IRF-3 and IRF-7 in response to viruses for IFN-alpha/beta gene induction. *Immunity*, 13(4):539–548, October 2000.

[13] Isabelle Marie, Joan E Durbin, and David E Levy. Differential viral induction of distinct interferon- genes by positive feedback through interferon regulatory factor-7.

[14] Kenya Honda, Hideyuki Yanai, Hideo Negishi, Masataka Asagiri, Mitsuharu Sato, Tatsuaki Mizutani, Naoya Shimada, Yusuke Ohba, Akinori Takaoka, Nobuaki Yoshida, and Tadatsugu Taniguchi. IRF-7 is the master regulator of type-I interferon-dependent immune responses. *Nature*, 434(7034):772–777, April 2005.

[15] Arun Prakash, Eric Smith, Chien-Kuo Lee, and David E. Levy. Tissue-specific positive feedback requirements for production of type I interferon following virus infection. *The Journal of Biological Chemistry*, 280(19):18651–18657, May 2005.

[16] Shun Chen, Zhen Wu, Mingshu Wang, and Anchun Cheng. Innate Immune Evasion Mediated by Flaviviridae Non-Structural Proteins. *Viruses*, 9(10):291, October 2017.

[17] Siddharth Balachandran and Amer A. Beg. Defining Emerging Roles for NF-B in Antivirus Responses: Revisiting the Interferon- Enhanceosome Paradigm. *PLOS Pathogens*, 7(10):e1002165, October 2011.

[18] Daniel Panne, Tom Maniatis, and Stephen C. Harrison. An atomic model of the interferon-beta enhanceosome. *Cell*, 129(6):1111–1123, June 2007.

[19] Kenya Honda, Akinori Takaoka, and Tadatsugu Taniguchi. Type I Inteferon Gene Induction by the Interferon Regulatory Factor Family of Transcription Factors. *Immunity*, 25(3):349–360, September 2006.

[20] Sergei V. Kotenko, Grant Gallagher, Vitaliy V. Baurin, Anita Lewis-Antes, Meiling Shen, Nital K. Shah, Jerome A. Langer, Faruk Sheikh, Harold Dickensheets, and Raymond P. Donnelly. IFN-lambdas mediate antiviral protection through a distinct class II cytokine receptor complex. *Nature Immunology*, 4(1):69–77, January 2003.

[21] D. L. Krebs and D. J. Hilton. SOCS proteins: negative regulators of cytokine signaling. *Stem Cells (Dayton, Ohio)*, 19(5):378–387, 2001.

[22] Véronique François-Newton, Gabriel Magno de Freitas Almeida, Béatrice Payelle-Brogard, Danièle Monneron, Lydiane Pichard-Garcia, Jacob Piehler, Sandra Pellegrini, and Gilles Uzé. Usp18-based negative feedback control is induced by type i and type iii interferons and specifically inactivates interferon α response. *PloS one*, 6(7):e22200, 2011.

[23] Benjamin D. Maier, Luis U. Aguilera, Sven Sahle, Pascal Mutz, Priyata Kalra, Christopher Dächert, Ralf Bartenschlager, Marco Binder, and Ursula Kummer. Stochastic dynamics of Type-I interferon responses. *PLOS Computational Biology*, 18(10):e1010623, October 2022.

[24] X. Y. Fu, D. S. Kessler, S. A. Veals, D. E. Levy, and J. E. Darnell. ISGF3, the transcriptional activator induced by interferon alpha, consists of multiple interacting polypeptide chains. *Proceedings of the National Academy of Sciences of the United States of America*, 87(21):8555–8559, November 1990.

[25] William M. Schneider, Meike Dittmann Chevillotte, and Charles M. Rice. Interferon-Stimulated Genes: A Complex Web of Host Defenses. *Annual Review of Immunology*, 32(Volume 32, 2014):513–545, March 2014.

[26] Ke Shuai and Bin Liu. Regulation of JAK–STAT signalling in the immune system. *Nature Reviews Immunology*, 3(11):900–911, November 2003.
