# Supplementary material for: Integrative modelling of innate immune response dynamics during virus infection
Source: PLoS Comput Biol. 2026 Jun 22;22(6):e1014395. doi: 10.1371/journal.pcbi.1014395 (PMC13322630; doi:10.1371/journal.pcbi.1014395)
Supplement: S2 Text — (PDF) [file pcbi.1014395.s002.pdf]

1 Evaluation of model predictions against Aunins et al. (2018) data

Time-course measurements of intracellular HCV RNA, extracellular virus, NS3, and core proteins were digitized from [1] using WebPlotDigitizer. Where reported, mean values and associated standard deviations were extracted separately to retain information on experimental variability.

To enable direct quantitative comparison between model predictions and experimental measurements, all quantities were expressed in molecule-number units. A cell volume of 4 pL was assumed, corresponding to a conversion factor of 2400 molecules cell^{-1} per nM. Model parameters were transformed consistently under this scaling, ensuring that all state variables and observables are represented in the same physical units as the digitized data.

In Aunins et al.[1], which combines experimental measurements with mathematical modeling, the initial conditions are not specified as independently measured quantities but are effectively determined within a joint fitting framework. Intracellular species are assumed to be negligible at the time of infection, and both kinetic parameters and initial states are inferred simultaneously by fitting to time-course RNA and protein data. Consequently, the effective initial condition represents a latent, data-constrained state rather than a directly imposed experimental value.

In the present model, viral entry is represented explicitly, such that the initial extracellular virus V_0 is not directly comparable to the inoculum used in [1]. To obtain a comparable initialization, we adopt an analogous data-driven calibration strategy for V_0. Specifically, V_0 was determined such that the simulated total intracellular RNA ($R_{\text{cyt}} + R_{\text{CM}} + RC_{\text{CM}}$), expressed in molecules cell^{-1}, matches the experimental value at $t \approx 8.97$ h (8.83 molecules cell^{-1}). All other state variables were initialized at their no-virus steady states. The resulting model trajectory over 48 h was then compared, as shown in S1 Fig.

References

[1] T. R. Aunins, K. A. Marsh, G. Subramanya, S. L. Uprichard, A. S. Perelson, and A. Chatterjee. Intracellular Hepatitis C Virus Modeling Predicts Infection Dynamics and Viral Protein Mechanisms. *J. Virol.*, 92(11), 06 2018.
