# Supplementary material for: Integrative modelling of innate immune response dynamics during virus infection
Source: PLoS Comput Biol. 2026 Jun 22;22(6):e1014395. doi: 10.1371/journal.pcbi.1014395 (PMC13322630; doi:10.1371/journal.pcbi.1014395)
Supplement: S1 Table — (PDF) [file pcbi.1014395.s003.pdf]

S1 Table. Description of model variables and their initial condition values used in simulation

Eqn no	Species	Description	Value	Ref.
S3	V_0	Virus Input	0.0298 nM	(calibrated to match early intracellular RNA levels using Aunins et al. (2018) data [1])
S4	V_I	Internalized virus	0	
S5	R_{cyt}	Cytoplasmic viral RNA	0	
S6	P_S	Viral Structural Proteins	0	
S7	P_{NS}	Viral Non-Structural Proteins	0	
S8	RC_{CM}	Compartmentalized replication complexes	0	
S9	R_{CM}	(+) RNA in CMs	0	
S10	RNA_{ds}	dsRNA in cytoplasm	0	
S11	V_T	Viral Titre	0	
S12	RIGI	PRR sensor	5.34 nM	[2]
S13	aRIGI	Activated RIGI	0	
S14	MAVS	Mitochondrial antiviral-signaling protein	277.76 nM	[2]
S15	aMAVS	Activated MAVS	0	
S16	IKKe	IκB kinase ε	3.08 nM	[2]
S17	aIKKe	Activated IKKe	0	
S18	TBK1	TANK-binding kinase 1	97.17 nM	[2]
S19	aTBK1	Activated TBK1	0	
S20	IRF3	Interferon regulatory factor 3	37.86 nM	[2]
S21	pIRF3	Phosphorylated IRF3	0	
S22	IRF7	Interferon regulatory factor 7	24 nM	[3]
S23	pIRF7	Phosphorylated IRF7	0	
S24	IKK	Inhibitor of kappa B kinase	37.97 nM	[2]
S25	aIKK	Activated IKK	0	
S26	$NFkB\text{-}IkBa_c$	Inactive NFκB in cytoplasm	11.36 nM	[2]
S27	$pNFkB_n$	Activated NFκB in nucleus	0	
S28	$NFkB_n$	NFκB in nucleus	0	
S29	$NFkB_c$	NFκB in cytoplasm	101.73 nM	[2]

Continued on next page

Eqn no	Species	Description	Value	Ref.
S30	$IkBa_c$	inhibitor of kappa B in cytoplasm	0	
S31	IFNb_m	Interferon β mRNA	0	
S32	IFNa_m	Interferon α mRNA	0	
S33	IFNl_m	Interferon λ mRNA	0	
S34	IFN_c	Interferon in cytoplasm		
S35	$IFNl_c$	Interferon λ in cytoplasm	0	
S36	IFNex	Extracellular interferon type-1	0	
S37	JAK	Janus Kinase 1	151.86 nM	[2]
S38	RJC	Receptor JAK complex	0	
S39	TYK	Tyrosine Kinase 2	20.70 nM	[2]
S40	RTKC	Receptor TYK Complex	0	
S41	IFNAR1	Interferon alpha receptor subunit 1	1000 nM	[4]
S42	IFNAR2	Interferon alpha receptor subunit 2	1000 nM	[4]
S43	IFNARd	Interferon receptor dimer	0	
S44	ARC	Activated Receptor Complex	0	
S45	$ARC\text{-}STAT2_c$	ARC and STAT2 complex in cytoplasm	0	
S46	$ARC\text{-}STAT12_c$	ARC, STAT2 and STAT1 complex in cytoplasm	0	
S47	$STAT1_c$	STAT 1 in cytoplasm	1114.68 nM	[2]
S48	$STAT2_c$	STAT2 in cytoplasm	6.50 nM	[2]
S49	$STAT1_n$	STAT1 in nucleus	0	
S50	$STAT2_n$	STAT2 in nucleus	0	
S51	$IRF9_c$	IRF9 in cytoplasm	45 nM	[4]
S52	$IRF9_n$	IRF9 in nucleus	0	
S53	PSC_c	Phosphorylated STATs complex in cytoplasm	0	
S54	PSC_n	Phosphorylated STATs complex in nucleus	0	
S55	$STAT2\text{-}IRF9_n$	STAT2 and IRF9 complex in nucleus	0	
S56	$STAT2\text{-}IRF9_c$	STAT2 and IRF9 complex in cytoplasm	0	
S57	$ISGF3_c$	ISGF3 in cytoplasm	0	
S58	$ISGF3_n$	ISGF3 in nucleus	0	
S59	B_U	Open ISGF3 binding sites	500 nM	[4]
S60	B_O	Occupied ISGF3 binding sites	0	

Continued on next page

Eqn no	Species	Description	Value	Ref.
S61	PIAS	Protein inhibitor of activated STAT	41.96 nM	[2]
S62	PIAS-ISGF3	PIAS and ISGF3 complex	0	
S63	CP	Cytoplasmic Phosphatase	20 nM	[4]
S64	NP	Nuclear Phosphatase	40 nM	[4]
S65	ISGF3-CP	ISGF3 and CP complex	0	
S66	ISGF3-NP	ISGF3 and NP complex	0	
S67	PSC-CP	PSC and CP complex	0	
S68	PSC-NP	PSC and NP complex	0	
S69	B_O-NP	Occupied ISGF3 binding sites—NP	0	
S70	ISGn	Negative regulator ISG	0	
S71	ISGav_m	Antiviral ISG mRNA	0	
S72	ISGav	Antiviral ISG	0	
S73	ISGn_m_n	Negative regulator ISG mRNA in nucleus	0	
S74	IRF9_m_n	IRF9 mRNA in nucleus	0	
S75	IRF7_m	IRF7 mRNA	0	
S76	ISGn_m_c	Negative regulator ISG mRNA in cytoplasm	0	
S77	IRF9_m_c	IRF9 mRNA in cytoplasm	0	

References

[1] T. R. Aunins, K. A. Marsh, G. Subramanya, S. L. Uprichard, A. S. Perelson, and A. Chatterjee. Intracellular Hepatitis C Virus Modeling Predicts Infection Dynamics and Viral Protein Mechanisms. *J. Virol.*, 92(11), 06 2018.

[2] Sandy S Burkart, Darius Schweinoch, Jamie Frankish, Carola Sparn, Sandra Wüst, Christian Urban, Marta Merlo, Vladimir G Magalhães, Antonio Piras, Andreas Pichlmair, et al. High-resolution kinetic characterization of the rig-i-signaling pathway and the antiviral response. *Life Science Alliance*, 6(10), 2023.

[3] Xiufen Zou, Xueshuang Xiang, Yan Chen, Tao Peng, Xuelian Luo, and Zishu Pan. Understanding inhibition of viral proteins on type i ifn signaling pathways with modeling and optimization. *Journal of theoretical biology*, 265(4):691–703, 2010.

[4] Tim Maiwald, Annette Schneider, Hauke Busch, Sven Sahle, Norbert Gretz, Thomas S Weiss, Ursula Kummer, and Ursula Klingmüller. Combining theoretical analysis and experimental data generation reveals irf9 as a crucial factor for accelerating interferon α-induced early antiviral signalling. *The FEBS journal*, 277(22):4741–4754, 2010.
