# Supplementary material for: Integrative modelling of innate immune response dynamics during virus infection
Source: PLoS Comput Biol. 2026 Jun 22;22(6):e1014395. doi: 10.1371/journal.pcbi.1014395 (PMC13322630; doi:10.1371/journal.pcbi.1014395)
Supplement: S3 Table — The estimation method listed reflects the approach employed in the cited reference. (PDF) [file pcbi.1014395.s005.pdf]

S3 Table. JEV-specific viral life-cycle parameters.

The estimation method listed reflects the approach employed in the cited reference.

Parameter	Description	Value	Reference	Method
$k_{a,V}$	Effective virus generation rate	3.2×10^{-7} nM^{-1} min^{-1}	[1]	iterative approximate Bayesian computation (iABC) with χ^2 convergence against experimental viral RNA and protein time-course data.
$k_{e,V}$	Export rate of R_{CM} into the cytoplasm	0.12×10^{-2} min^{-1}		
$k_{r,V}$	(+)RNA synthesis rate per RC_{CM}	0.062 min^{-1}		
$k_{c,V}$	Formation rate of RC_{CM}	0.640 nM^{-1} min^{-1}		
$k_{t,V}$	Protein production rate per R_{cyt}	0.027×10^{2} min^{-1}		
τ	Time constant for functional development of CMs	156 min		
$N_{\text{C, V}}$	RC_{CM} carrying capacity of the host cell	0.504		
$\mu_{r,V}$	Degradation rate of R_{cyt}	0.0042 min^{-1}		
$\mu_{p,V}$	Degradation rate of PS and PNS	0.0018 min^{-1}		
$\mu_{V,V}$	Degradation rate of extracellular virus, V_T	0.1×10^{-3} min^{-1}		
nSP	Number of PS per virus particle	180		

References

[1] Harsh Chhajer, Vaseef A Rizvi, and Rahul Roy. Life cycle process dependencies of positive-sense rna viruses suggest strategies for inhibiting productive cellular infection. *J R Soc Interface*, 2021.
