# Supplementary material for: Integrative modelling of innate immune response dynamics during virus infection
Source: PLoS Comput Biol. 2026 Jun 22;22(6):e1014395. doi: 10.1371/journal.pcbi.1014395 (PMC13322630; doi:10.1371/journal.pcbi.1014395)
Supplement: S4 Table — The estimation method listed reflects the approach employed in the cited reference. (PDF) [file pcbi.1014395.s006.pdf]

S4 Table. Description of model parameters and their values

The estimation method listed reflects the approach employed in the cited reference.

Parameter	Description	Value	Reference	Method
$k_{\mathrm{en,V}}$	Rate of virus entry	0.0020 min^{-1}	[1]	Log-likelihood minimisation with trust-region algorithm and Latin hypercube multi-start.
$k_{f,V}$	Rate of fusion of the viral and endosomal membranes	5.2×10^{-4} min^{-1}	[1]	
μ_{V_I}	Decay rate of internalised virus	0.33×10^{-2} min^{-1}	[1]	
$k_{l,V}$	Rate of transfer of dsRNA into cytoplasm	4.67×10^{-4} min^{-1}	[1]	
k_s	Rate of IFN secretion	1.65×10^{-2} min^{-1}	[1]	
μ_{IFN}	degradation rate of IFN	2.5×10^{-3} min^{-1}	[1]	
k_{69}	Dissociation rate of ARC by ISGn (negative regulator ISG)	1.32 nM^{-1} min^{-1}	[2]	Genetic algorithm minimizing an objective function fitted to population and single-cell flow-cytometry data.
k_{70}	Translation rate of IRF9 mRNA	0.864 min^{-1}	[2]	
k_{72}	Rate of production of ISGn mRNA	0.147 min^{-1}	[2]	
k_{73}	Translocation of ISGn mRNA from nucleus to cytoplasm	4.89 min^{-1}	[2]	
k_{74}	Rate of production of IRF9 mRNA	0.33 min^{-1}	[2]	
k_{75}	Translocation rate of IRF9 mRNA from cytoplasm to nucleus	0.876 min^{-1}	[2]	
k_{77}	Degradation rate of ISGn mRNA	4.36×10^{-2} min^{-1}	[2]	
k_{78}	Degradation rate of IRF9 mRNA	1.39×10^{-2} min^{-1}	[2]	
$k_{transISGn}$	Translation rate of ISGn mRNA	0.63 min^{-1}	[2]	
k_{79}	Translation rate of IRF7 mRNA	43.87 min^{-1}	[3]	Global optimisation (random search/genetic algorithm) fitted to single-cell IRF7 expression data.
μ_{IRF7}	Degradation rate of IRF7	1.67×10^{-3} min^{-1}	[4]	Adopted from experimentally constrained literature values for CVB3 infection kinetics.
τ_5	Half-life of IRF7 mRNA	120 min	[5]	Manual fitting of ODE model to IFN-β-stimulated human dendritic cell time-course data.

Continued on next page

Parameter	Description	Value	Reference	Method
k_{76}	Rate of production of IRF7 mRNA	6×10^{-5} min^{-1}	[5]	
degARCISGn	Degradation rate of ARC by ISGn (negative regulator ISG)	0.0147 nM^{-1} min^{-1}	[6]	Maximum-likelihood fitting to immunoblot and qRT-PCR data with profile-likelihood identifiability analysis.
degRecISGn	Degradation rate of Receptor by ISGn (negative regulator ISG)	1.7×10^{-4} nM^{-1} min^{-1}	[6]	
kinhISGn	Inhibition of formation of ARC by ISGn (negative regulator ISG)	889.4 nM^{-1}	[6]	
k_{71}	Rate of dephosphorylation of pIRF7	10^{-3} min^{-1}	[7]	Negative log-likelihood minimisation with Latin hypercube multi-start and profile-likelihood identifiability analysis.
k_{m_IFNa}	IFN α mRNA production rate	1.67×10^{-4} nM^{-1} min^{-1}	[7]	
k_{m_IFNb}	IFN β mRNA production rate	1.99×10^{-4} nM^{-1} min^{-1}	[7]	
k_{m_IFNl}	IFN λ mRNA production rate	0.154 min^{-1}	[7]	
k_{m_ISGav}	IFN-independent antiviral ISG mRNA production rate	7×10^{-3} min^{-1}	[7]	
k_{ISGav_m}	IFN-dependent antiviral ISG mRNA production rate	15.41 min^{-1}	[7]	
μ_{m_IFNa}	Degradation rate of IFN α mRNA	2.3×10^{-3} min^{-1}	[7]	
μ_{m_IFNb}	Degradation rate of IFN β mRNA	2.8×10^{-3} min^{-1}	[7]	
μ_{m_IFNl}	Degradation rate of IFN λ mRNA	3.6×10^{-3} min^{-1}	[7]	
μ_{ISGRNA}	Degradation rate of antiviral ISG mRNA	2.8×10^{-3} min^{-1}	[7]	
μ_{ISGav}	Degradation rate of antiviral ISG	2.1×10^{-4} min^{-1}	[7]	
V_{cyt}	Cytoplasmic volume	1.2×10^{-12} L	[7]	
V_n	Nuclear volume	4.66×10^{-13} L	[7]	
Vc2n	Volume ratio: cytoplasm to nucleus	2.585	[7]	
Vn2c	Volume ratio: nucleus to cytoplasm	0.387	[7]	
b_{IRF3}	Dephosphorylation rate of IRF3	10^{-3} min^{-1}	[7]	
b_{RIGI}	Deactivation rate of RIGI	9.3×10^{-2} min^{-1}	[7]	

Continued on next page

Parameter	Description	Value	Reference	Method
b_{KINASE}	Deactivation rate of TBK1, IKKϵ and IKK	8×10^{-2} min^{-1}	[7]	Negative log-likelihood minimisation with Latin hypercube multi-start and profile-likelihood identifiability analysis.
k_{trans_IFNl}	Translation rate of IFN λ	6.2×10^{-4} min^{-1}	[7]	
$k_{transp\_NF\kappa B}$	Translocation rate of NFκB from nucleus to cytoplasm	2.3×10^{-4} min^{-1}	[7]	
μ_{IFNl}	degradation rate of IFNλ	$1.6\ \times10^{-4}$ min^{-1}		
$\mu_{I\kappa B\alpha}$	degradation rate of IκBα	$6.8\ \times10^{-3}$ min^{-1}	[7]	
μ_{RIGI}	degradation rate of RIGI	$1.4\ \times10^{-3}$ min^{-1}	[7]	
$k_{t,\ ISG\ RNA}$	translation rate of antiviral ISG (ISGav)	1.2×10^{-4} min^{-1}	[7]	
k_{IFN}	Translation rate of IFN mRNA	$5.98\ \times10^{-5}$ min^{-1}	[7]	
k_{IKK}	Activation of IKK	4.82×10^{-2} nM^{-1} min^{-1}	[7]	
$k_{IKKe\text{-}TBK1}$	Activation of IKKϵ and TBK1	10^{-3} nM^{-1} min^{-1}	[7]	
$k_{IRF3\text{-}IKKe\text{-}TBK1}$	IRF3 phosphorylation rate by IKKϵ and TBK1	0.0170 nM^{-1} min^{-1}	[7]	
$k_{TFBS\_IFN\alpha}$	IFNα induction by JAK-STAT pathway	0 min^{-1}	[7]	
$k_{TFBS\_IFN\beta}$	IFN β induction by JAK-STAT pathway	0 min^{-1}	[7]	
$k_{TFBS\_IFN\lambda}$	IFNλ induction by JAK-STAT pathway	0 min^{-1}	[7]	
k_{act}	Activation of inactive NFκB by IKK	0.00211 nM^{-1} min^{-1}	[7]	
k_{deph}	Dephosphorylation rate constant of nuclear pNFκB	0.0519 min^{-1}	[7]	
$k_{expr,\ I\kappa B\alpha}$	IκBα production rate	9.2×10^{-5} min^{-1}	[7]	
k_{inh_p65}	Sequestering of NFκB by IκBα	0.03 nM^{-1} min^{-1}	[7]	
k_{RIGI_synt}	Basal level RIGI synthesis	$7.5\ \times10^{-3}$ nM min^{-1}	[7]	
k_{MAVS}	Activation of MAVS	9×10^{-3} nM^{-1} min^{-1}	[8]	Genetic algorithm with multi-parent crossover fitted to viral infection time-course data.
k_{RIGI}	Activation of RIGI	0.01 nM^{-1} min^{-1}	[8]	
b_{MAVS}	Deactivation rate of MAVS	0.219 min^{-1}	[8]	
k_1	TYK and IFNAR1 association rate	6 nM^{-1} min^{-1}	[9]	Random search in COPASI, selecting ≈1,000 acceptable sets from 10,000 evaluations within a ±20% threshold of immunoblot data.

Continued on next page

Parameter	Description	Value	Reference	Method
k_{10}	Dissociation rate of ARC and $STAT2_c$ from ARC-STAT2$_c$	240 min^{-1}	[9]	
k_{11}	Association rate of $STAT1_c$ with $ARC - STAT2_c$	0.12 nM^{-1} min^{-1}	[9]	
k_{12}	Dissociation rate of $STAT1_c$ from $ARC - STAT2_c$	240 min^{-1}	[9]	
k_{13}	Formation of PSC_c	480 min^{-1}	[9]	
k_{14}	Association rate of PSC_c with $IRF9_c$ resulting in $ISGF3_c$	6 nM^{-1} min^{-1}	[9]	
k_{15}	Disassociation rate of $ISGF3_c$ into PSC_c and $IRF9_c$	6 min^{-1}		
k_{16}	Translocation rate of $ISGF3_c$ to nucleus	0.9 L min^{-1}	[9]	
k_{17}	Translocation rate of $ISGF3_n$ to cytoplasm	0 L min^{-1}	[9]	
k_{18}	Translocation rate of PSC_c to nucleus	0.9 L min^{-1}	[9]	
k_{19}	Translocation rate of PSC_n to cytoplasm	0 L min^{-1}	[9]	
k_2	TYK and IFNAR1 disassociation rate	3 min^{-1}	[9]	
k_{20}	Association rate of PSC_n with $IRF9_n$ resulting in $ISGF3_n$	0.6 nM^{-1} min^{-1}	[9]	
k_{21}	Dissociation rate of $ISGF3_n$ into $IRF9_n$ and PSC_n	0.6 min^{-1}	[9]	
k_{22}	Association rate of $ISGF3_n$ to the B_U	6 nM^{-1} min^{-1}	[9]	Random search in COPASI, selecting ≈1,000 acceptable sets from 10,000 evaluations within a ±20% threshold of immunoblot data.
k_{23}	Dissociation rate of B_O into $ISGF3_n$ and B_U	6 min^{-1}	[9]	
k_{27}	Basal production of IRF9$_c$	0.3 nM min^{-1}	[9]	
k_{29}	Degradation rate of IRF9$_c$	0.006 min^{-1}	[9]	
k_3	JAK and IFNAR2 association rate	6 nM^{-1} min^{-1}	[9]	
k_{31}	Degradation rate of ISGn	0.03 min^{-1}	[9]	
k_{32}	Dissociation rate of ARC	0.018 min^{-1}	[9]	
k_{34}	Deactivation rate of ARC into IFNARd	0.6 min^{-1}	[9]	
k_{35}	Degradation rate of $IRF9_n$	0.006 min^{-1}	[9]	
k_{36}	Dissociation of $STAT2$-$IRF9_c$ into $STAT2_c$	0.006 min^{-1}	[9]	

Continued on next page

Parameter	Description	Value	Reference	Method
k_{37}	Dissociation of $STAT2\text{-}IRF9_n$ into $STAT2_n$	0.006 min^{-1}	[9]	
k_{38}	Association of $ISGF3_c$ with CP	0.06 nM^{-1} min^{-1}	[9]	
k_{39}	Dissociation of ISGF3-CP into $ISGF3_c$ and CP	12 min^{-1}	[9]	
k_4	JAK and IFNAR2 disassociation rate	3 min^{-1}	[9]	
k_{40}	Dephosphorylation of $ISGF3_c$ complex by CP	0.18 min^{-1}	[9]	
k_{41}	Association of PSC_c with CP	0.06 nM^{-1} min^{-1}	[9]	
k_{42}	Dissociation of PSC-CP into PSC_c and CP	12 min^{-1}	[9]	
k_{43}	Dephosphorylation of PSC_c complex by CP	0.18 min^{-1}	[9]	
k_{44}	Association of PSC_n with NP	0.6 nM^{-1} min^{-1}		
k_{45}	Dissociation of PSC-NP into PSC_n and NP	6 min^{-1}	[9]	
k_{46}	Dephosphorylation of PSC_n complex by NP	6 min^{-1}	[9]	
k_{47}	Association of $ISGF3_n$ with NP	0.6 nM^{-1} min^{-1}	[9]	
k_{48}	Dissociation of ISGF3-NP into $ISGF3_n$ and NP	6 min^{-1}	[9]	Random search in COPASI, selecting ≈1,000 acceptable sets from 10,000 evaluations within a ±20% threshold of immunoblot data.
k_{49}	Dephosphorylation of $ISGF3_n$ complex by NP	0.12 min^{-1}	[9]	
k_5	Association rate of free IFN with RJC and RTKC resulting in IFNARd	0.6 nM^{-2} min^{-1}	[9]	
k_{50}	Association of B_O with NP	0.006 nM^{-1} min^{-1}	[9]	
k_{51}	Dissociation of $B_O\text{-}NP$ into B_O and NP	6 min^{-1}	[9]	
k_{52}	Dissociation of B_O into subunits	6 min^{-1}	[9]	
k_{53}	Association of $ISGF3_n$ with PIAS	6 nM^{-1} min^{-1}	[9]	
k_{54}	Dissociation of ISGF3-PIAS into $ISGF3_n$ and PIAS	6 min^{-1}	[9]	
k_{56}	Translocation rate of $STAT1_c$ to nucleus	0.075 nM^{-1} min^{-1}	[9]	
k_{57}	Translocation rate of $STAT1_n$ to cytoplasm	0.6 L min^{-1}	[9]	

Continued on next page

Parameter	Description	Value	Reference	Method
k_{58}	Translocation rate of $STAT2_c$ to nucleus	0.0049 L min^{-1}	[9]	
k_{59}	Translocation rate of $STAT2_n$ to cytoplasm	0.084 L min^{-1}	[9]	
k_6	Disassociation rate of free IFN from RJC and RTKC	0.6 min^{-1}	[9]	
k_{60}	Association of $STAT2_c$ and $IRF9_c$	0.6 nM^{-1} min^{-1}	[9]	
k_{61}	Dissociation of $STAT2{-}IRF9_c$ into $STAT2_c$ $IRF9_c$	0.6 min^{-1}	[9]	Random search in COPASI, selecting ≈1,000 acceptable sets from 10,000 evaluations within a ±20% threshold of immunoblot data.
k_{62}	Association of $STAT2_n$ and $IRF9_n$	0.6 nM^{-1} min^{-1}	[9]	
k_{63}	Dissociation of $STAT2{-}IRF9_n$ into $STAT2_n$ $IRF9_n$	0.6 min^{-1}	[9]	
k_{64}	Translocation rate of $STAT2 - IRF9_c$ to nucleus	0.075 L min^{-1}	[9]	
k_{65}	Translocation rate of $STAT2 - IRF9_n$ to cytoplasm	0.084 L min^{-1}	[9]	
k_{66}	Translocation rate of $IRF9_c$ to nucleus	1.2 L min^{-1}	[9]	
k_{67}	Translocation rate of $IRF9_n$ to cytoplasm	0.3 L min^{-1}	[9]	
k_7	Formation of ARC from IFNARd	0.3 min^{-1}	[9]	
k_8	Association of STAT2c with ARC from $STAT2c_IRF9$	0.12 nM^{-1} min^{-1}	[9]	
k_9	Association of free STAT2c with ARC	0.12 nM^{-1} min^{-1}	[9]	

References

[1] Carolin Zitzmann, Bianca Schmid, Alessia Ruggieri, Alan S Perelson, Marco Binder, Ralf Bartenschlager, and Lars Kaderali. A coupled mathematical model of the intracellular replication of dengue virus and the host cell immune response to infection. *Frontiers in microbiology*, 11:725, 2020.

[2] Benjamin D Maier, Luis U Aguilera, Sven Sahle, Pascal Mutz, Priyata Kalra, Christopher Dächert, Ralf Bartenschlager, Marco Binder, and Ursula Kummer. Stochastic dynamics of type-i interferon responses. *PLOS Computational Biology*, 18(10):e1010623, 2022.

[3] Luis U Aguilera, Christoph Zimmer, and Ursula Kummer. A new efficient approach to fit stochastic models on the basis of high-throughput experimental data using a model of irf7 gene expression as case study. *BMC systems biology*, 11:1–14, 2017.

[4] Aaron B Lopacinski, Andrew J Sweatt, Christian M Smolko, Elise Gray-Gaillard, Cheryl A Borgman, Millie Shah, and Kevin A Janes. Modeling the complete kinetics of coxsackievirus b3 reveals human determinants of host-cell feedback. *Cell systems*, 12(4):304–323, 2021.

[5] Liang Qiao, Hannah Phipps-Yonas, Boris Hartmann, Thomas M Moran, Stuart C Sealfon, and Fernand Hayot. Immune response modeling of interferon β-pretreated influenza virus-infected human dendritic cells. *Biophysical journal*, 98(4):505–514, 2010.

[6] Frédérique Kok, Marcus Rosenblatt, Melissa Teusel, Tamar Nizharadze, Vladimir Gonçalves Magalhães, Christopher Dächert, Tim Maiwald, Artyom Vlasov, Marvin Wäsch, Silvana Tyufekchieva, et al. Disentangling molecular mechanisms regulating sensitization of interferon alpha signal transduction. *Molecular systems biology*, 16(7):e8955, 2020.

[7] Sandy S Burkart, Darius Schweinoch, Jamie Frankish, Carola Sparn, Sandra Wüst, Christian Urban, Marta Merlo, Vladimir G Magalhães, Antonio Piras, Andreas Pichlmair, et al. High-resolution kinetic characterization of the rig-i-signaling pathway and the antiviral response. *Life Science Alliance*, 6(10), 2023.

[8] Xiufen Zou, Xueshuang Xiang, Yan Chen, Tao Peng, Xuelian Luo, and Zishu Pan. Understanding inhibition of viral proteins on type i ifn signaling pathways with modeling and optimization. *Journal of theoretical biology*, 265(4):691–703, 2010.

[9] Tim Maiwald, Annette Schneider, Hauke Busch, Sven Sahle, Norbert Gretz, Thomas S Weiss, Ursula Kummer, and Ursula Klingmüller. Combining theoretical analysis and experimental data generation reveals irf9 as a crucial factor for accelerating interferon α-induced early antiviral signalling. *The FEBS journal*, 277(22):4741–4754, 2010.
